# Quantification of lipid sorting during clathrin-mediated endocytosis

**DOI:** 10.1038/s41556-026-02068-7

**Published:** 2026-09-29

**Authors:** H. Mathilda Lennartz, Susana da Costa Nunes, Kristin Böhlig, Aditya Chhatre, Radek Šachl, Sascha M. Kuhn, Lior Moneta, Pavel Barahtjan, Wan Yee Yau, Juan M. Iglesias-Artola, Martin Hof, Lukáš Opálka, Carl D. Modes, Alf Honigmann, André Nadler

**Affiliations:** 1 Max Planck Institute of Molecular Cell Biology and Genetics, Dresden, Germany; 2 Biotechnologisches Zentrum, Center for Molecular and Cellular Bioengineering (CMCB), Technische Universität Dresden, Dresden, Germany; 3 J. Heyrovský Institute of Physical Chemistry, Academy of Sciences of the Czech Republic v.v.i., Prague, Czech Republic; 4 Center for Systems Biology Dresden, Dresden, Germany; 5 École polytechnique fédérale de Lausanne, Lausanne, Switzerland; 6 Department of Organic and Bioorganic Chemistry, Faculty of Pharmacy in Hradec Králové, Charles University, Hradec Králové, Czech Republic; 7 Cluster of Excellence Physics of Life, TU Dresden, Dresden, Germany

**Keywords:** Endocytosis, Membrane lipids, Membrane lipids

## Abstract

Clathrin-mediated endocytosis is a major transport route for proteins from the plasma membrane to the interior of the cell. While the recruitment of cargo proteins to clathrin-coated pits is well understood, it remains an open question whether lipids are also sorted by this process. Here, to address this question, we combined super-resolution stimulated emission depletion imaging of bifunctional lipid probes with mathematical modelling. Quantification of ten different lipid species revealed significant differences in pit partitioning, ranging from slight enrichment to moderate exclusion. We find that the lipid asymmetry in the plasma membrane is sufficient to explain the observed trend. Taken together, our findings imply that clathrin-mediated endocytosis has a minor selectivity for cytoplasmic leaflet lipids, but overall does not substantially contribute to lipid sorting compared with non-vesicular trafficking.

## Abstract (web summary)

Lennartz et al. evaluate lipid enrichment in clathrin-coated pits and observe that the differential lipid partitioning into clathrin-coated pits is largely driven by the lipid asymmetry of the plasma membrane.

## Main

Clathrin-mediated endocytosis (CME) is the primary route of bulk cellular ingestion and lipid uptake during retrograde vesicular transport. More than 50 proteins are involved in the formation of clathrin-coated vesicles having a diameter of 60–120 nm. They are recruited to the endocytic site in a highly controlled temporal order during three stages: initiation, maturation and fission^1–4^.

Cargo proteins are enriched in clathrin-coated pits (CCPs) by specific interactions with adaptors via short peptide motifs and/or posttranslational modifications^5,6^. Much less is known about the extent and the mechanisms of lipid sorting during CME. Previous studies have shown that PI(4,5)P_2_ localization to CCPs is a necessary precondition for pit initiation and maturation. However, there is no compelling evidence for PI(4,5)P_2_ enrichment in the pit^7–9^. Further reports suggest that lipids associated with ordered membrane domains are depleted in CCPs, such as cholesterol, gangliosides and glycosylphosphatidylinositol, whereas lipids associated with disordered membrane domains should accumulate^10–14^. These studies are based on indirect evidence for lipid sorting into CCPs by monitoring the endosomal levels of fluorescent tracers such as glycophosphatidylinositol-anchored proteins, fluorescent lipid analogues and lipid-binding toxins. Notably, reporters such as cholera toxin cluster lipids, which affect sorting and uptake mechanisms^15^. Fluorescent lipid analogues do not reliably reflect the behaviour of native lipids, as the introduction of a bulky chromophore alters the chemical properties of the parent molecule^16–18^. More generally, quantification of lipid uptake on the level of endosomal structures convolutes plasma membrane and endosomal sorting mechanisms. A systematic investigation of lipid sorting during CME based on direct evidence at the pit has yet to be carried out for a broad selection of structurally diverse lipid species.

Here we use our recently introduced approach for imaging cellular lipid localization^19,20^ based on bifunctional lipid probes^21–23^ to directly quantify lipid partitioning into CCPs. We adapted our methodology for super-resolution stimulated emission depletion (STED) microscopy and captured temporally resolved CCP maturation for a library of ten different lipid species to investigate lipid enrichment in early- and late-stage CCPs. Combination with theoretical modelling allowed us to quantify true lipid enrichment in late-stage CCPs from the apparent enrichment caused by microscope convolution of the membrane geometry during pit maturation. Our analysis revealed significant differences between lipid species, suggesting differential partitioning into CCPs. By comparing different sorting mechanisms, we conclude that differential lipid partitioning into CCPs is primarily driven by the lipid asymmetry of the plasma membrane. Lipid partitioning into CCPs is thus fully determined by plasma membrane lipid composition and transbilayer lipid distribution, implying that CME combines selective protein and non-selective lipid transport.

## Results

### Super-resolution STED resolves lipid species localization in CCPs

**Extended Data Fig. 1:**
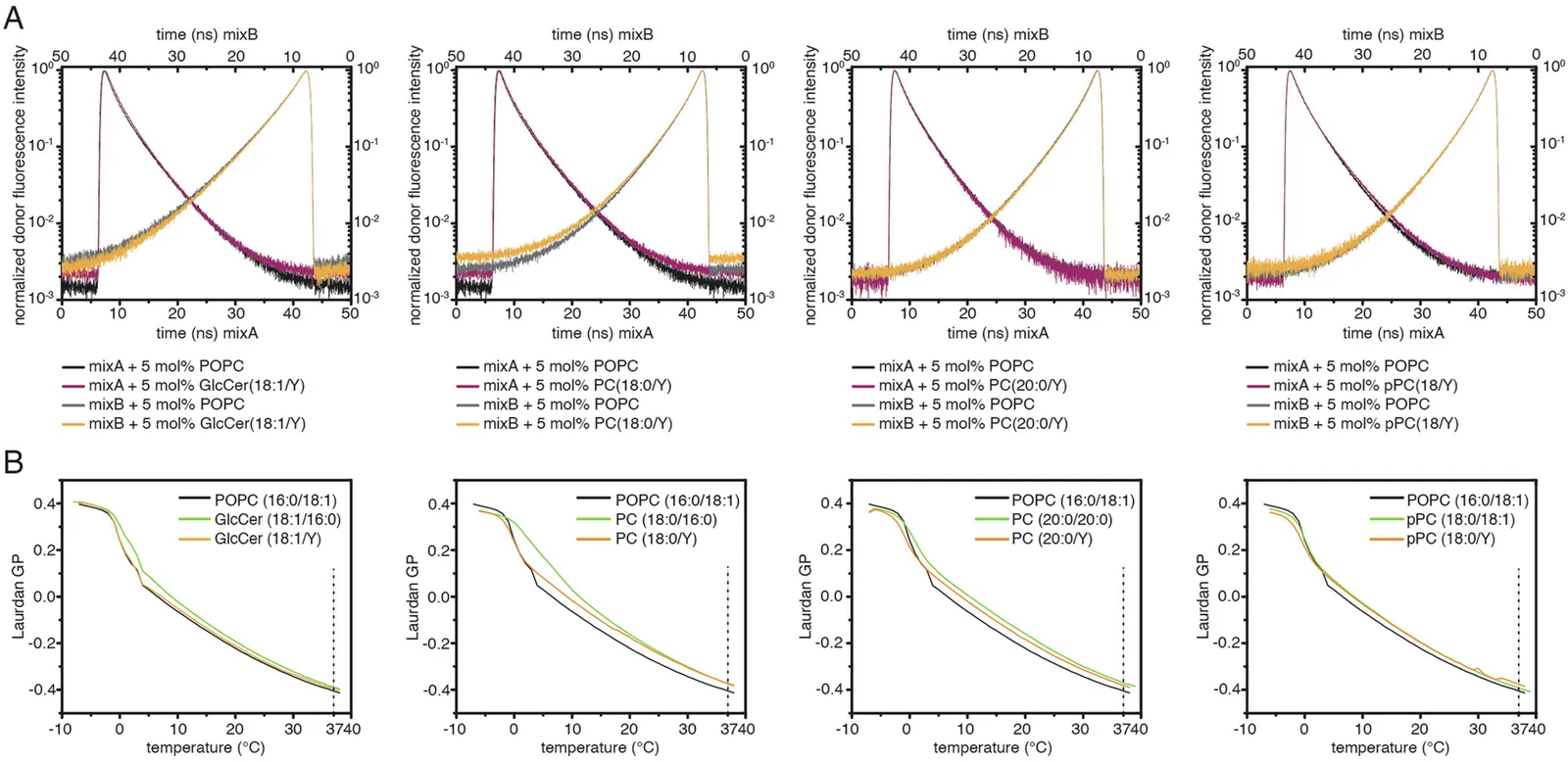
Biophysical characterization of bifunctional lipid probes by comparison to native counterparts. **a**: Formation of ganglioside nanodomains leading to faster deexcitation of Bodipy-FL-GM_1_ donors via FRET is unaffected by replacing the closest structurally related native lipid with the bifunctional variant for all probes. Lipids tested included GlcCer(18:1/Y), PC(18:0/Y), PC(20:0/Y), and pPC(18/Y). MixA: DOPC/Cholesterol/SM (65/25/10 in mol%) containing lipid nanodomains with the average radius of 9 nm and occupying 45%^56^ of the membrane surface, mixB: DOPC/Cholesterol/SM (75/25/10 in mol%) + 4% GM1 with the nanodomains of the radius 99 nm and occupying 50%^57^ of the membrane surface. Data was acquired for 1 replicate, n(vesicles per condition): at least 5. **b**: Laurdan temperature scans indicate that biophysical implications of introducing a bifunctional fatty acid are small. Equivalent FRET and Laurdan experiments for the additional lipids used in this study have been previously reported by Iglesias-Artola et al.^19^. Data was acquired for 1 replicate. Numbers of measured vesicles per replicate are provided in the Source Data. Source data

To quantify the role of CME for sorting lipids at the plasma membrane, we optimized our recently developed lipid imaging workflow^19^ for super-resolution STED microscopy. The lipid imaging approach relies on near-native bifunctional lipids, which are modified with a diazirine and alkyne moiety. The diazirine can be photochemically cross-linked to neighbouring biomolecules, while the alkyne is used for labelling by click chemistry. These probes closely resemble native lipids with regard to their metabolism and biophysical properties. The combined effect of the modifications is approximately equivalent to the introduction of one additional double bond^19,24,25^ (Extended Data Fig. 1).

**Fig. 1:**
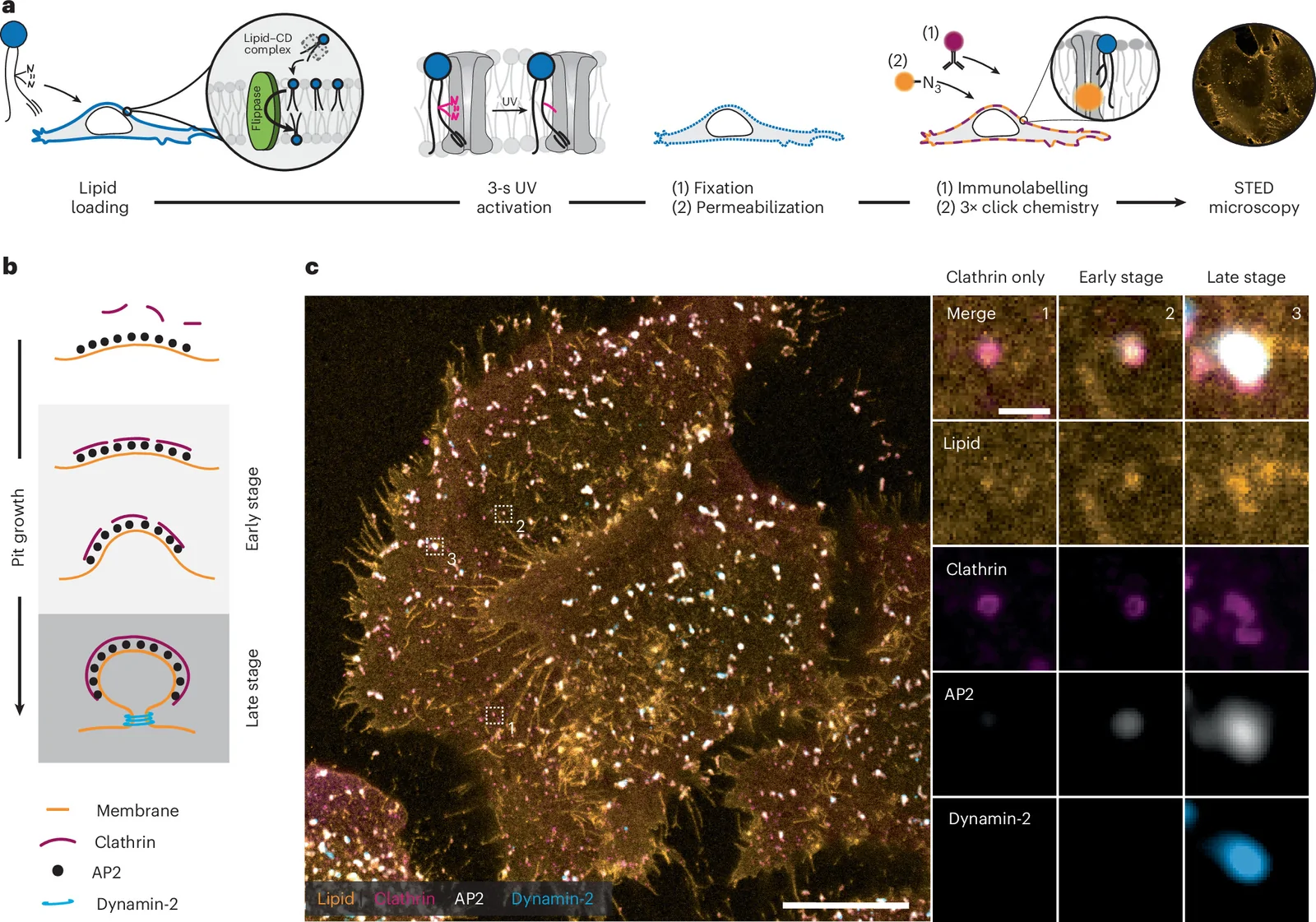
Super-resolution lipid imaging of lipid localization in CCPs. **a**, Super-resolution lipid imaging workflow. Lipid probes were loaded into the plasma membrane of living cells using α-methylcyclodextrin (CD). Photo-cross-linking to proteins with a 3-s UV light pulse was followed immediately by chemical fixation. Samples were permeabilized, stained by immunolabelling and click chemistry, and imaged using four-colour confocal microscopy with the lipid and clathrin-channel super-resolved by STED. **b**, Stages of the growth of a CCP were classified using protein markers into clathrin-only structures, early-stage CCPs positive for AP2 and clathrin, and late-stage CCPs positive for AP2, clathrin and dynamin-2. **c**, A representative STED image from four biological replicates of the basal membrane of an SK-MEL2 cell loaded with bifunctional PC(16:0|Y) (orange) is shown, stably expressing dynamin-2–GFP (blue). Clathrin (purple) and AP2 (grey) were immunolabelled. Scale bars, 10 µm (full image), 200 nm (magnified areas). Numbers of acquired STED images per biological replicate are provided in the source data. Source data

**Extended Data Fig. 2:**
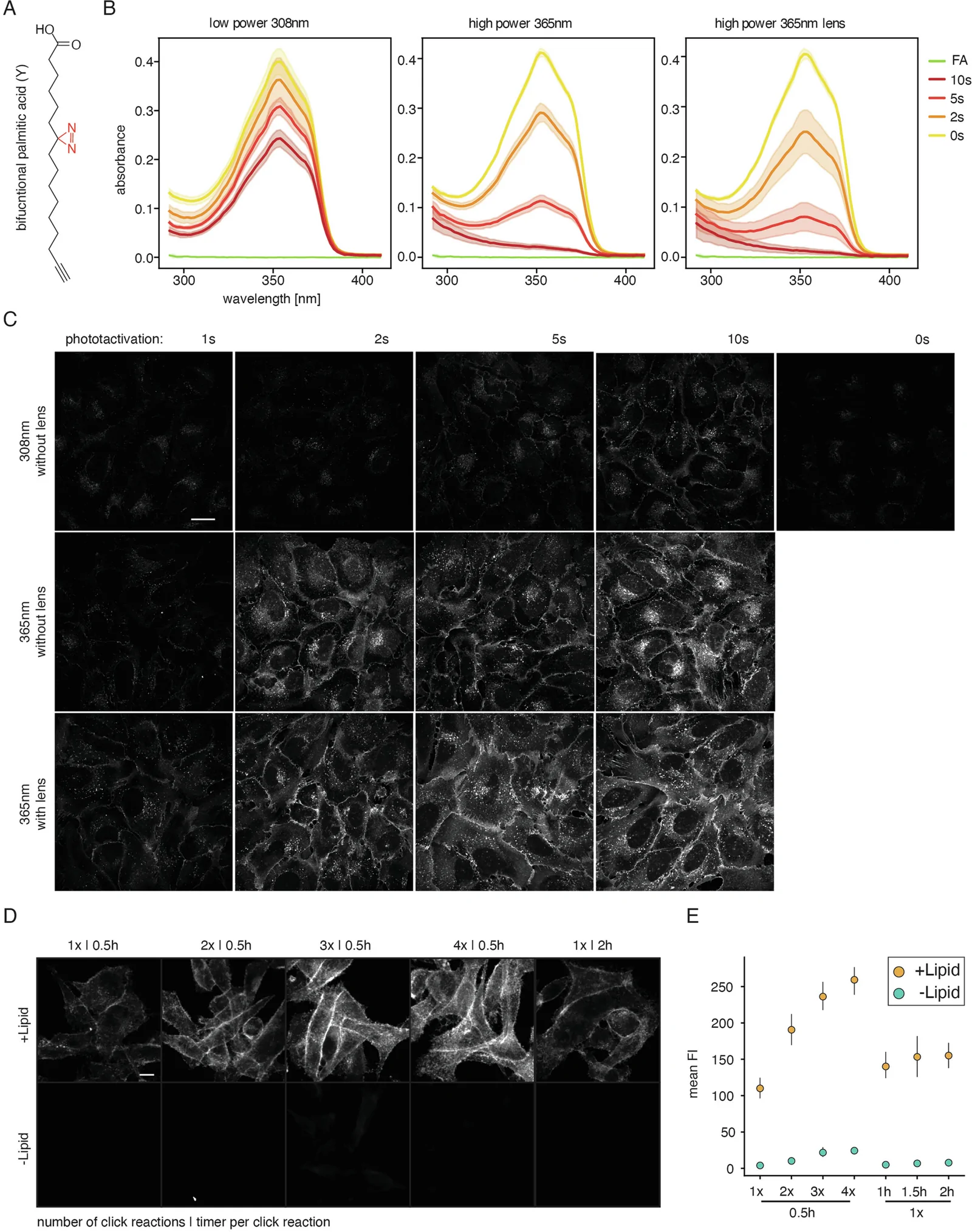
Characterization of the diazirine photo-crosslinking and click chemistry efficiency. **a**: The chemical structure of the previously reported^19^ bifunctional palmitic fatty acid (Y) is shown. **b**: The diazirine spectrum of Y is measured at 100 mM concentration in DMSO. Spectra were measured after different exposure times of 0 s (yellow), 2 s (orange), 5 s (red), and 10 s (deep red) with a recently reported 308 nm LED^19^ compared to 2 high power LEDs at 365 nm with and without a collimating lens. The spectra of the native fatty acid (FA, green) lack a diazirine peak. Photoactivation experiments were performed in technical triplicates, 1 well per replicate. Mean values (line) and standard deviation (shaded area) are depicted. **c**: U2OS wildtype cells were loaded for 4 min with bifunctional sphingomyelin, UV activated for different time intervals and with different LEDs. Signal intensities are scaled equally for all images. Scale bar is in 20 µm. Representative images from 1 biological replicate are shown, 6 images per replicate condition were acquired. **d**: SKMEL wildtype cells were loaded with PC(16:0/Y) for 4 min. Samples were stained by CuAAC for 1, 2, 3, or 4 times for a duration of 30 min per stain or 1 time for the respective prolonged duration of 1 h, 1.5 h, or 2 h. Scale bar: 10 µm. Representative images of 3 technical replicates are shown, that yielded similar results. **e**: The mean intensity values of fluorescent images (from D) were determined. Error bars represent the standard deviation. The experiment was performed in 3 technical replicates, n(ROI): 18. Fields of view per replicate are provided in the Source Data. Source data

**Extended Data Fig. 3:**
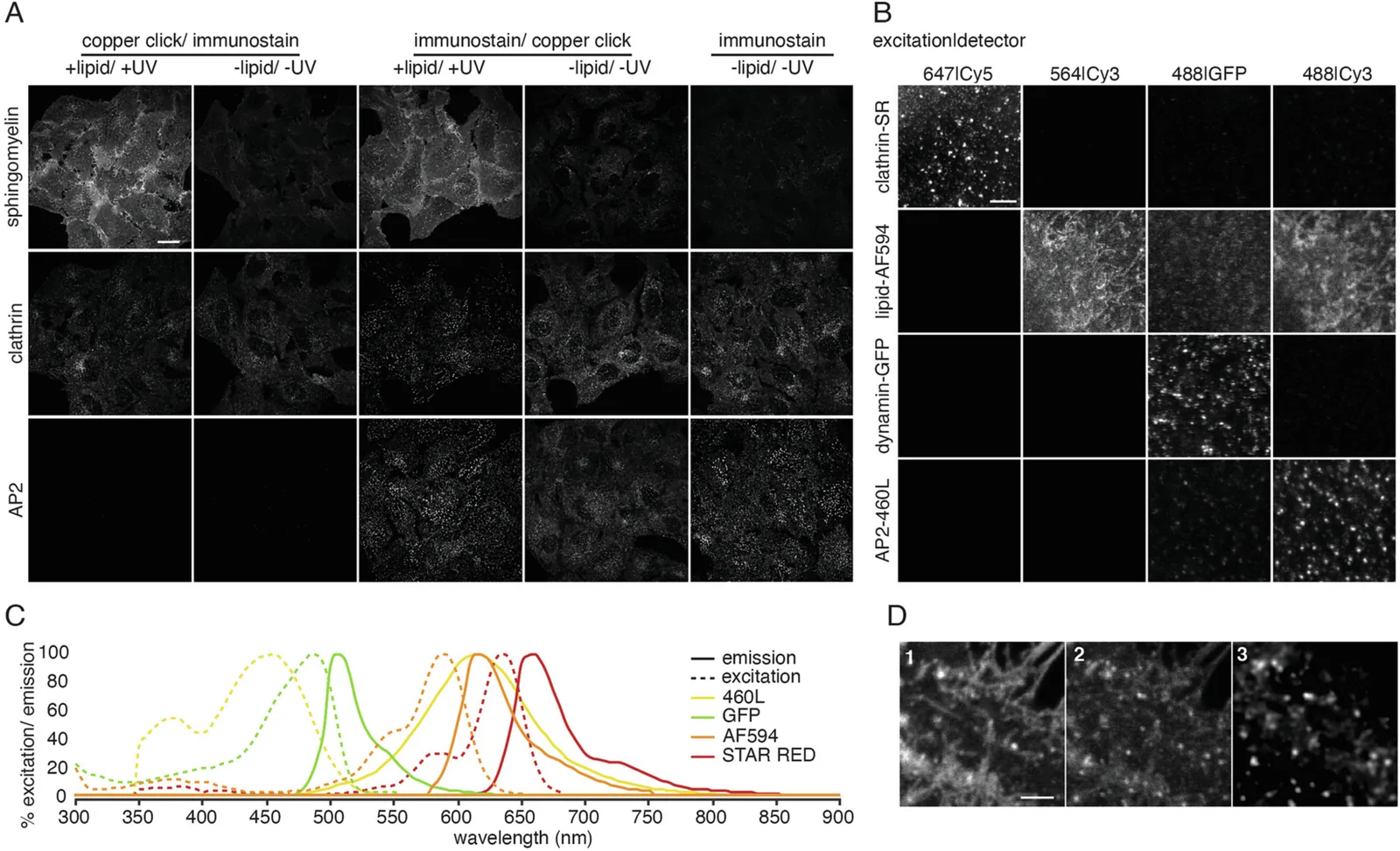
Optimization of the sample preparation and imaging setup. **a**: U2OS wildtype cells were fed for 4 min with bifunctional sphingomyelin, photoactivated, chemically fixed, and permeabilized. Samples were then either first stained by CuAAC followed by immunolabelling (copper click/ immunostain) of clathrin and AP2 or first immunolabelled and then stained by CuAAC (immunostain/ copper click). As a control for the immunolabelling efficiency, a sample was only immune-labelled but not stained by CuAAC (immunostain). The experiment was performed in one replicate, representative images are shown. Scale bar: 20 µm. **b**: SK-MEL cells with a single label, either clathrin – STAR RED (row 1), lipid – AlexaFluor594 (row 2), dynamin-2-GFP (row 3), or AP2 – 460 L were imaged with all four imaging channels to determine bleed-through. Excitation lasers used were 647 nm (clathrin), 564 nm (lipid), and 488 nm (dynamin-2, AP2). Emission was collected in the Cy5 (clathrin), Cy3 (lipid. AP2), or GFP (dynamin-2) channel. All images of one excitation/ emission class are scaled to the same brightness and contrast. Representative images of 1 replicate are shown, 5 images per condition were acquired. Scale bar: 2 µm. **c**: Spectra of the used fluorophores abberior 460 L, GFP, AlexaFluor594, and abberior STAR RED generated with the FluoroFinder Spectral Viewer^58^. **d**: The confocal image of the AF594 (1), the confocal image of the 460 L (2), and the linear de-mixed image of 460 L (3) are shown. Scale bar: 2 µm. The dataset includes 3 biological replicates per lipid species, that yielded similar results. N(image of single cells): 26 - 31. Fields of view per replicate are provided in the Source Data. Source data

Briefly, bifunctional lipids were loaded into the outer leaflet of the plasma membrane and fixed via subsequent ultraviolet (UV) light and chemical cross-linking (Fig. 1a). To restrict lipid localization to the plasma membrane, lipids were UV cross-linked shortly after lipid loading, and cells were fixed chemically using a protocol adapted from a previous study^26^ to ensure conservation of CCP ultrastructure. UV cross-linking time was minimized using high-powered light-emitting diodes (LEDs) at 365 nm with a collimation lens to maximize photon flux (Extended Data Fig. 2a–c and Supplementary Data 1). Subsequently, samples were permeabilized to remove non-cross-linked probes and to allow for immunolabelling of CCP reporter proteins. Lipid–protein conjugates were fluorescently labelled by repeated copper-catalysed click chemistry (in the following referred to as click chemistry) (Extended Data Figs. 2d,e and 3a). UV cross-linking and fixative addition were completed within <10 s, which allowed snapshots of CCPs to be captured at different maturation stages^27^, under conditions that do not affect major membrane trafficking pathways^24^.

We used the well-characterized Sloan-Kettering melanoma 2 (SK-MEL2) dynamin-2 green fluorescent protein (GFP) reporter cell line^28^ in combination with immunostaining of clathrin and adaptor protein 2 (AP2) to classify CCPs according to their maturation stages. Structures positive for AP2 and clathrin were classified as early-stage pits^8,27^, whereas structures additionally positive for dynamin-2 were classified as late-stage pits^27,29^. Structures positive only for clathrin were classified as clathrin-only structures (Fig. 1b,c). Lipids and clathrin were imaged at super-resolution (STED), while dynamin-2 and AP2 were acquired in diffraction-limited confocal mode (Extended Data Fig. 3b–d). Taken together, this workflow allowed us to resolve lipid species in CCPs in different maturation stages.

### Quantification of lipid partitioning into CCPs

**Fig. 2:**
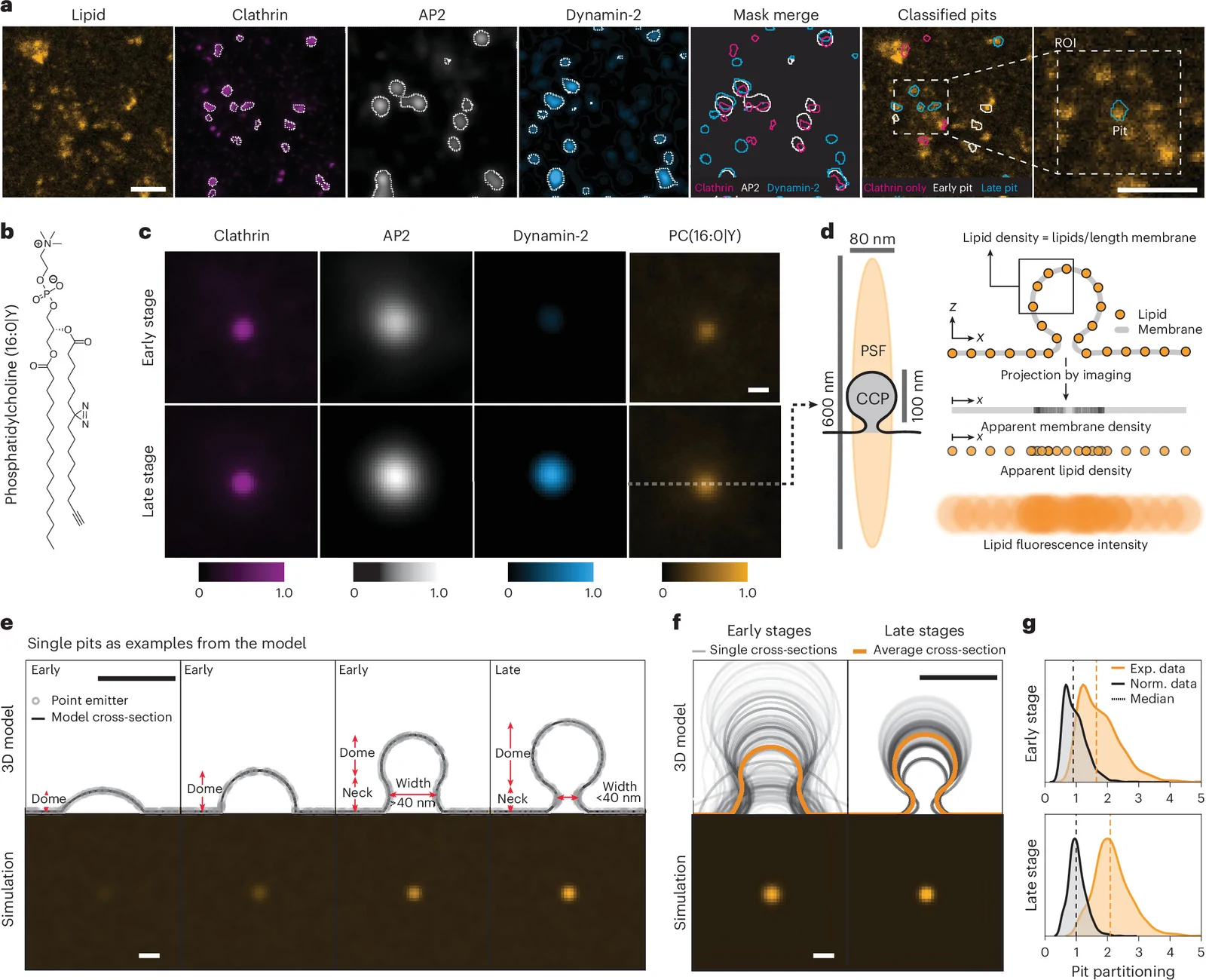
Quantification of lipid partitioning in CCPs. **a**, Image analysis pipeline. The super-resolved clathrin signal (purple) was used to generate a segmentation mask (white dotted overlay). Pits were classified as clathrin only (purple) and early-stage (white) or late-stage (blue) pits, depending on their overlap with the AP2 and dynamin-2 masks generated from the respective channels. Apparent lipid partitioning was calculated by measuring the mean lipid intensities of individual pits and division by the mean lipid intensity of the surrounding plasma membrane (ROI). Scale bars, 1 µm. A total of 38 STED images across 4 biological replicates were acquired. **b**, Chemical structure of PC(16:0|Y). **c**, Average four-colour fluorescence images for early- and late-stage pits. Fluorescent images of CCPs were centred on the centroid of the clathrin mask before averaging. Colour bars indicate the averaged fluorescence intensity in arbitrary units. Scale bar, 100 nm. Averages were acquired from 4 biological replicates and 38 individual STED images; *N* (early CCPs), 3,990; *n* (late CCPs), 2,801. **d**, A scheme is shown on the left for the dimensions of the experimentally determined point spread function of the microscope in relation to a late-stage CCP. On the right, the process of convolution during imaging is depicted. A pit cross-section with even point emitter distribution is projected into one dimension, resulting in higher apparent membrane densities and lipid densities at the region of the pit invagination. After blurring by the PSF of the microscope, the pit appears brighter than the surrounding flat membrane. Lipid density as a readout for lipid partitioning must be determined as the ratio of lipids over surface area. **e**, Top panels: cross-sections of modelled CCPs. Bottom panels: simulated STED for the pit geometries shown. Scale bars, 100 nm. Representative simulations are shown from a pool of 2,000 simulations. **f**, Average cross-sections (black) of all simulated pits (grey) and averaged simulated images are shown. Scale bars, 100 nm. Averages contain 1,000 cross-sections for early and late stages each. **g**, The pit partitioning distributions of early- and late-stage pits are shown for PC(16:0|Y) before (orange, exp. data) and after (grey, norm. data) normalizing to the median of the model. The respective medians of the data are shown as dashed lines. Numbers of acquired STED images per biological replicate are provided in the source data. Source data

To quantify lipid species partitioning into CCPs, we next segmented the highly resolved clathrin signal. We classified each pit based on the co-localization of the clathrin mask with the respective AP2 and dynamin-2 signal into clathrin-only structures and early (AP2 and clathrin positive) and late (AP2, dynamin-2, and clathrin positive) CCPs (Fig. 2a). Mean lipid signal intensities were determined for all pits over the segmented clathrin mask and the surrounding bulk plasma membrane in a square of 1.5 µm × 1.5 µm (region of interest (ROI)), excluding other pits and unidentified bright structures. Apparent lipid partitioning (*P*) was defined as the ratio of the mean lipid signal intensity within each clathrin mask and the surrounding plasma membrane.

**Extended Data Fig. 4:**
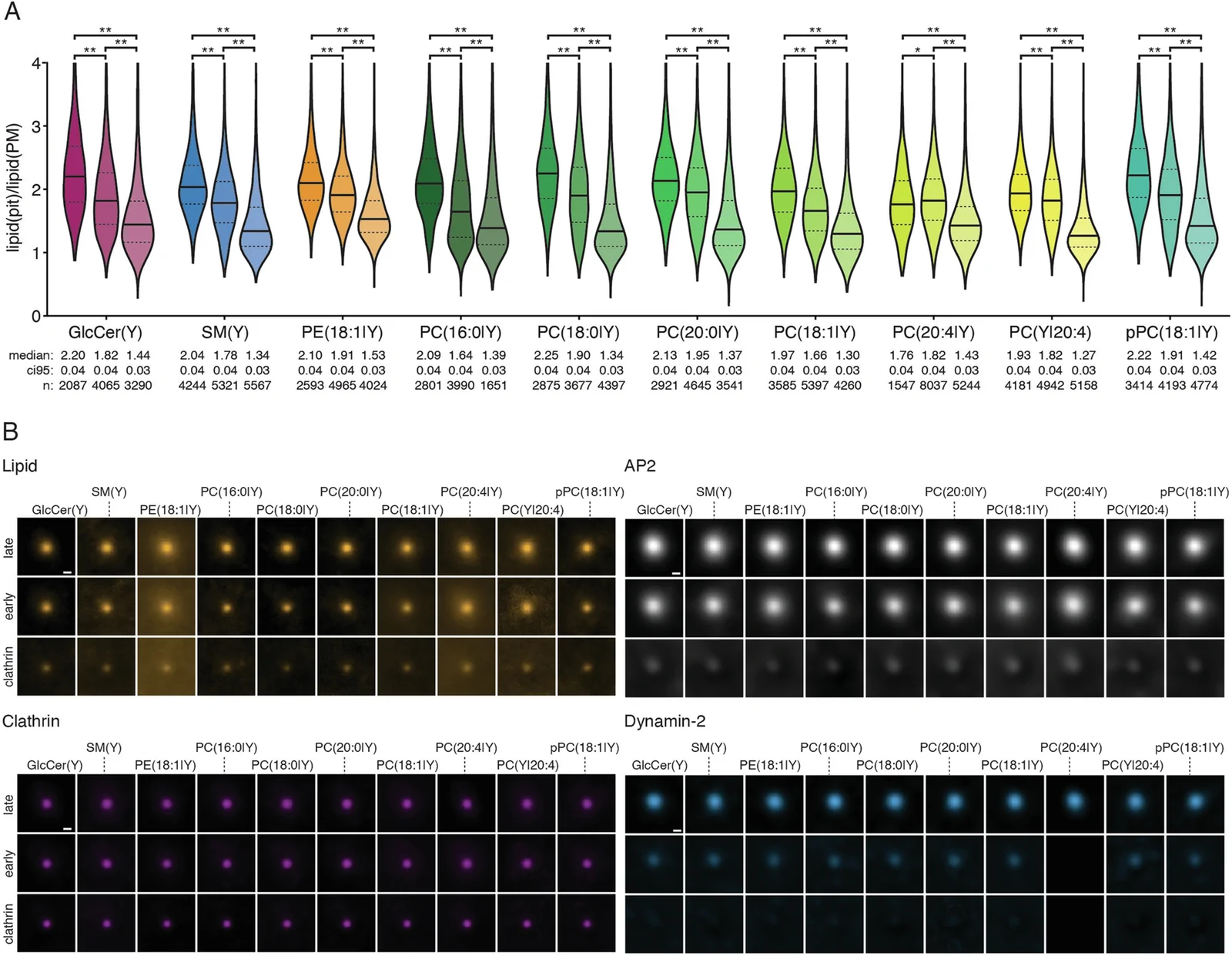
Lipid enrichments of clathrin-only, early-stage, and late-stage CCPs. **a**: Violin plots of the distributions of lipid pit enrichments over all measured individual pits are plotted for the different lipids and structure classes. The median (solid line) and quartiles (dotted line) are shown. For each lipid the structure class distributions differ significantly as assessed by a two-sided randomized permutation analysis (**: 0.000999; *: 0.001998). The experiment was performed for 3 biological replicates. Each datapoint represent a single pit (CCP). N(CCPs) are indicated in the figure. **b**: The averaged images of CCPs of all lipids and CCP stages are shown. All fluorescent images for all lipids species of CCPs (square of 1.5 µm × 1.5 µm) were centered to the centroid of the clathrin mask and averaged for clathrin only, early- and late-stage pits. The lipid (orange), clathrin (purple), AP2 (grey), and dynamin-2 (blue) channels are displayed separately. Scale bar: 100 nm. The experiment was performed for 3 biological replicates. N(CCPs) are indicated in A. Within one channel all images are scaled to the same brightness and contrast. The average images for early- and late-stages for PC(16:0|Y) are also displayed in main text Fig. 2c and all late-stage lipid images are also displayed in the main text Fig. 3b. Numbers of acquired STED images per biological replicate are provided in the Source Data. Source data

We found a general trend of increasing apparent lipid partitioning from early- to late-stage pits (Extended Data Fig. 4). This is shown using the bifunctional phosphatidylcholine PC(16:0|Y) (Y: bifunctional fatty acyl tail) as an example, a probe closely mimicking a common plasma membrane PC species, with an apparent partitioning from *P* = 1.64 ± 0.45 (median ± quartile) to *P* = 2.09 ± 0.36 for early- and late-stage pits, respectively (Fig. 2b,c,g). Next, to delineate to which extent this effect was indicative of true lipid accumulation in CCPs or whether it was a reflection of the changing membrane geometry during pit maturation, we modelled the convolution of the membrane geometry by the point spread function (PSF) of our microscope (Fig. 2d–f).

**Extended Data Fig. 5:**
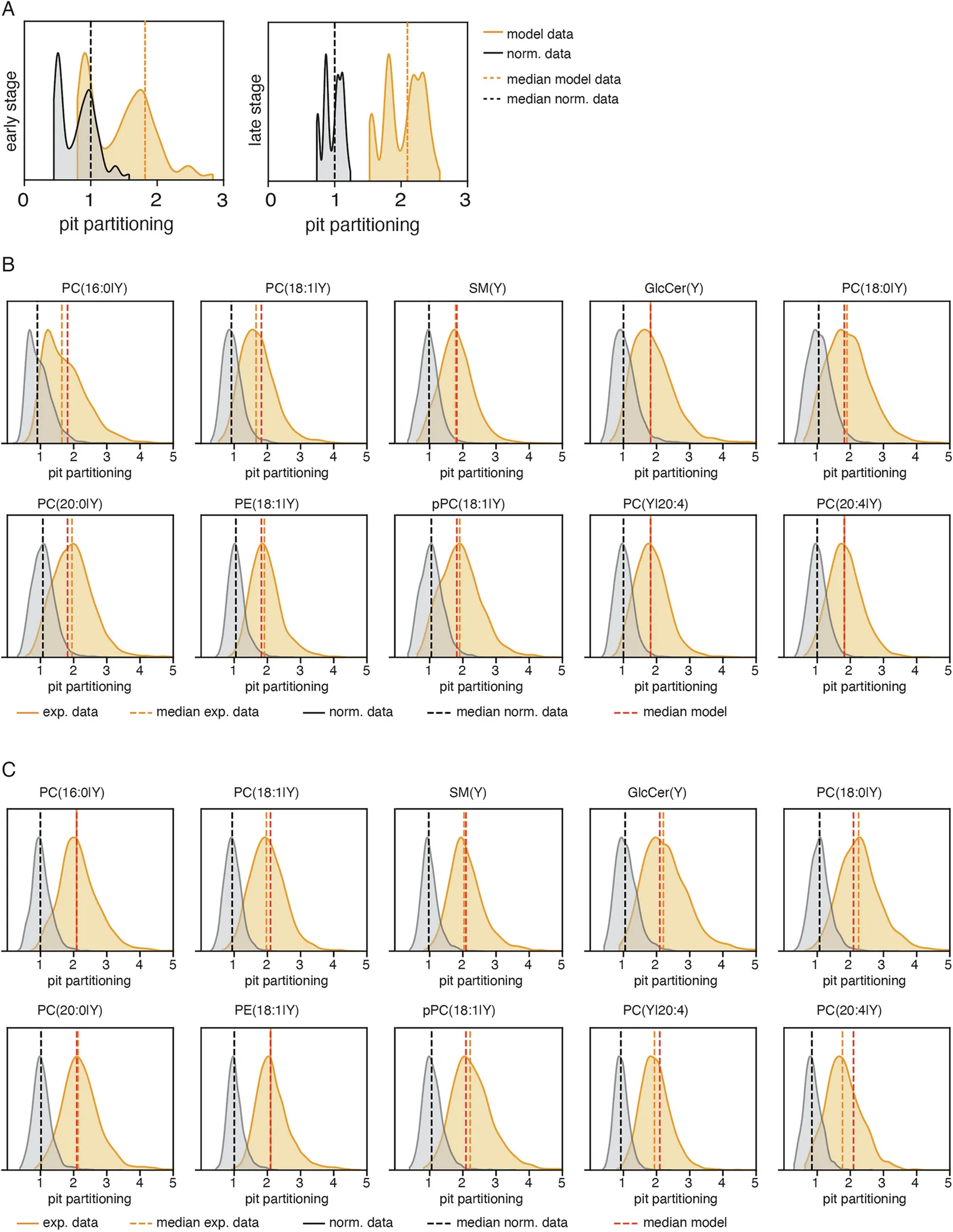
Assessment of the geometry model to normalize experimental data. **a**: Pit partitioning distributions of the model (orange) of early and late pits are overlayed with the respective distribution normalized to the median of the model (grey). The black and orange dotted lines indicate the median of the model before and after normalization respectively. N(pits late): 1000, n(pits early): 1000. B: The experimentally determined pit partitioning distributions (orange) of early (**b**) and late pits (**c**) are overlayed with the respective distribution normalized to the median of the model (grey). The lipid species is indicated on top of each plot. The black, red, and orange dotted lines indicate the median of the model, experimental data, and normalized experimental data respectively. The dataset includes 3 independent biological replicates. The number per pits for the data shown in **b** and **c** are indicated in Extended Data Fig. 3a. Numbers of acquired STED images per biological replicate are provided in the Source Data. Source data

Geometries of early- and late-stage pits were modelled by combining the shapes of partial orbs (pit dome) and catenoids (pit neck if applicable) using previously reported electron microscopy (EM) data of CCP membrane ultrastructures obtained from the SK-MEL2 cell line^30^ (Fig. 2f,g and Methods). Modelled pits were classified as late-stage if their neck diameter measured less than 40 nm, a curvature that has been reported to facilitate dynamin membrane association in vitro^31^. Next, an even distribution of point emitters per surface area was assumed across the 3D models, representing an even lipid distribution. From these distributions, STED images were simulated according to the PSF of our microscope. This model predicted a median (±quartile) of the apparent partitioning of 1.82 ± 0.51 for early stages and 2.10 ± 0.25 for late stages (Extended Data Fig. 5a), similar to the experimentally acquired values of PC(16:0|Y). To convert the convoluted apparent experimental partitioning values into absolute partitioning values, we normalized the apparent partitioning distribution to the median of the modelled distribution (Fig. 2g and Extended Data Fig. 5b). For PC(16:0|Y), this normalization provided a median partitioning value close to one for early- and late-stage pits (Fig. 2g), indicating close to even partitioning of the lipid between the plasma membrane and the CCP.

A closer inspection of the modelled pit structures showed that late-stage pits are much more consistent in their geometry than the early-stage pits (Fig. 2f). The early-stage pits contain a mixture of two geometries (domes without necks and domes with necks), but do not capture any transition geometries. A similar effect was not observed for late-stage CCPs, which include pits of similar geometries, indicating a more faithful recapitulation of the late-stage pit structures by the model. Respectively, early-stage pits show a much broader distribution of apparent partitioning values than late-stage pits, resulting in a larger expected error for the geometry correction (Extended Data Fig. 5). In addition, lipid partitioning into late-stage pits is much more meaningful in the context of lipid sorting during CME, as they represent the step immediately before the membrane fission event and thus more closely resemble the lipid composition of the clathrin-coated vesicles being released into the cytosol. Therefore, while we here provide measurements for both early- and late-stage pits, we base our conclusions regarding the lipid selectivity of CME primarily on the late-stage CCP lipid partitioning data.

Taken together, modelling pit geometry from known reference data allowed us to obtain absolute lipid partitioning values from convoluted experimental measurements of late-stage CCPs, a key prerequisite for quantitatively studying the role of lipids in membrane compartmentalization.

### Structurally diverse lipids partition differentially into CCPs

**Fig. 3:**
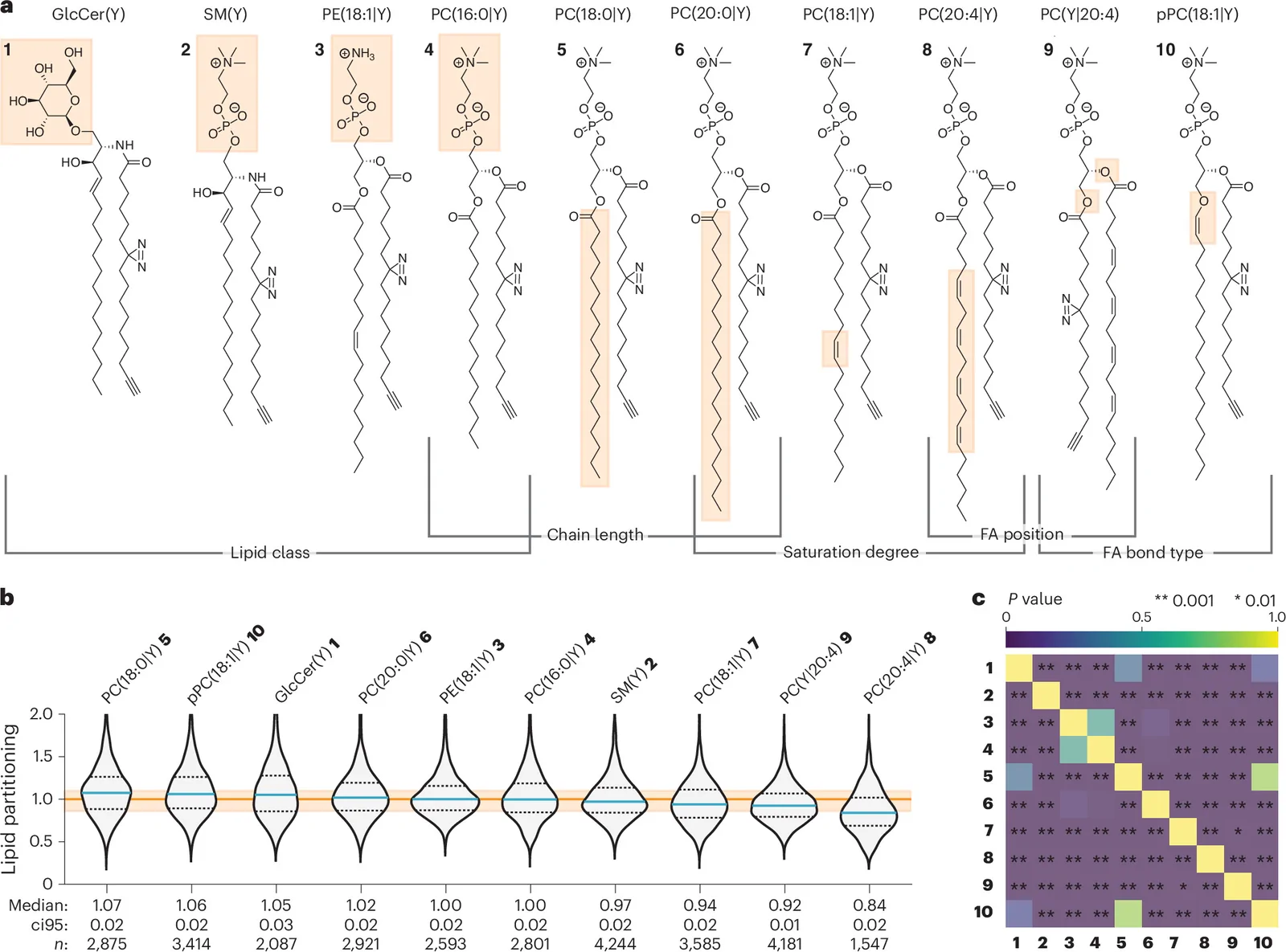
Comparison of ten different lipid species reveals differential partitioning in CCPs. **a**, Lipid library used to screen lipid enrichments in CCPs for structurally different lipids. Structural differences are highlighted in orange. Y, bifunctional fatty acid; GlcCer, glucosylceramide; SM, sphingomyelin; PE, phosphatidylethanolamine; PC, phosphatidylcholine; pPC, plasmalogen phosphatidylcholine; FA, fatty acid. **b**, Violin plots of normalized late-stage CCP lipid partitioning distributions for all lipid probes. Distributions are ordered from highest to lowest median partitioning. Median values (blue line) with the respective quartiles (dashed line) are shown. The ci95 and sample sizes (*n* (CCP)) are indicated below. The normalized median of the modelled distribution for a generic lipid evenly distributed between the plasma membrane and CCP is indicated by the orange line, with its respective quartiles (shaded area). The data were acquired from three biological replicates. **c**, Statistical significance between all lipids was determined by a two-sided randomized permutation test^55^. *P* values are colour coded. **P < 0.001 (all values: 0.000999); *P < 0.01 (ranging from 0.001998 to 0.008991); not marked (values ranging from 1 to 0.011988). Lipid species are indicated by numbers, as defined in **a**. Numbers of acquired STED images per biological replicate are provided in the source data. Source data

To compare the partitioning of different lipid species into CCPs, we next studied a library of ten bifunctional lipids that cover a large chemical space. The library included glucosylceramide (GlcCer(Y)), sphingomyelin (SM(Y)), phosphatidylethanolamine (PE(18:1|Y)) and a plasmalogen PC (pPC(18:1|Y)), as well as PC species of differential chain length, saturation degree and fatty acid positioning (Fig. 3a and Methods)^19,20^.

**Extended Data Fig. 6:**
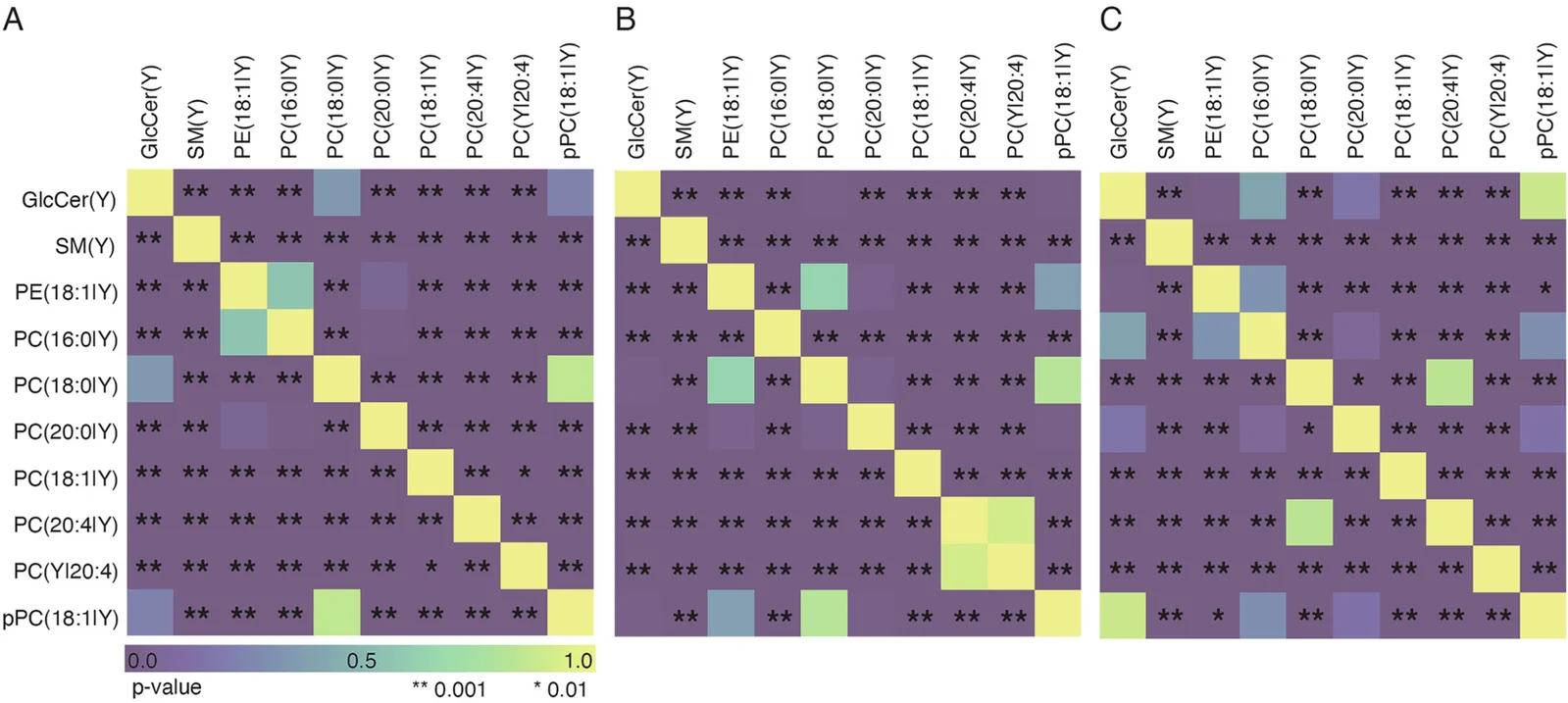
Two-sided randomized permutation test for the lipid enrichment distributions. Significance between lipid pit enrichments of late (**a**) and early pits (**b**) and clathrin-only (**c**) structures are shown. P-values per lipid pair were determined by a two-sided randomized permutation analysis^55^. P-values are color-coded with * marking values below 0.01 (ranging from 0.001998 – 0.008991), ** marking values below 0.001 (all values: 0.000999) or not marked (values ranging from 1 – 0.011988). The significance between lipid pit enrichments of late-stages (**a**) is also shown in the main Fig. 3c. Source data

Focusing on the quantification of absolute lipid partitioning for late-stage pits, we found that median partitioning values (±quantiles) ranged from 1.07 ± 0.16-fold to 0.84 ± 0.17-fold (Fig. 3b). While the differences in pit partitioning are subtle, statistical analysis showed that most differences are highly significant (Fig. 3c and Extended Data Fig. 6). Sorting lipid species by CCP partitioning provided the following order: PC(18:0|Y) > pPC(18:1|Y) > GlcCer(Y) > PC(20:0|Y) > PE(18:1|Y), PC(16:0|Y) > SM(Y) > PC(18:1|Y) > PC(Y|20:4) > PC(20:4|Y).

Overall, it appears that polyunsaturated lipids are slightly excluded from CCPs while saturated lipids tend to be slightly enriched in CCPs. However, this observation alone does not allow for the assignment of mechanistic roles of structural features such as headgroup, fatty acid saturation degree or chain length for CCP partitioning.

Taken together, quantification of lipid partitioning in late CCPs for 10 lipid species revealed significant differences of up to 21% between species, suggesting that lipids partition into CCPs in a species-specific manner.

### Lipid partitioning correlates with lipid melting temperature and lipid asymmetry

**Extended Data Fig. 7:**
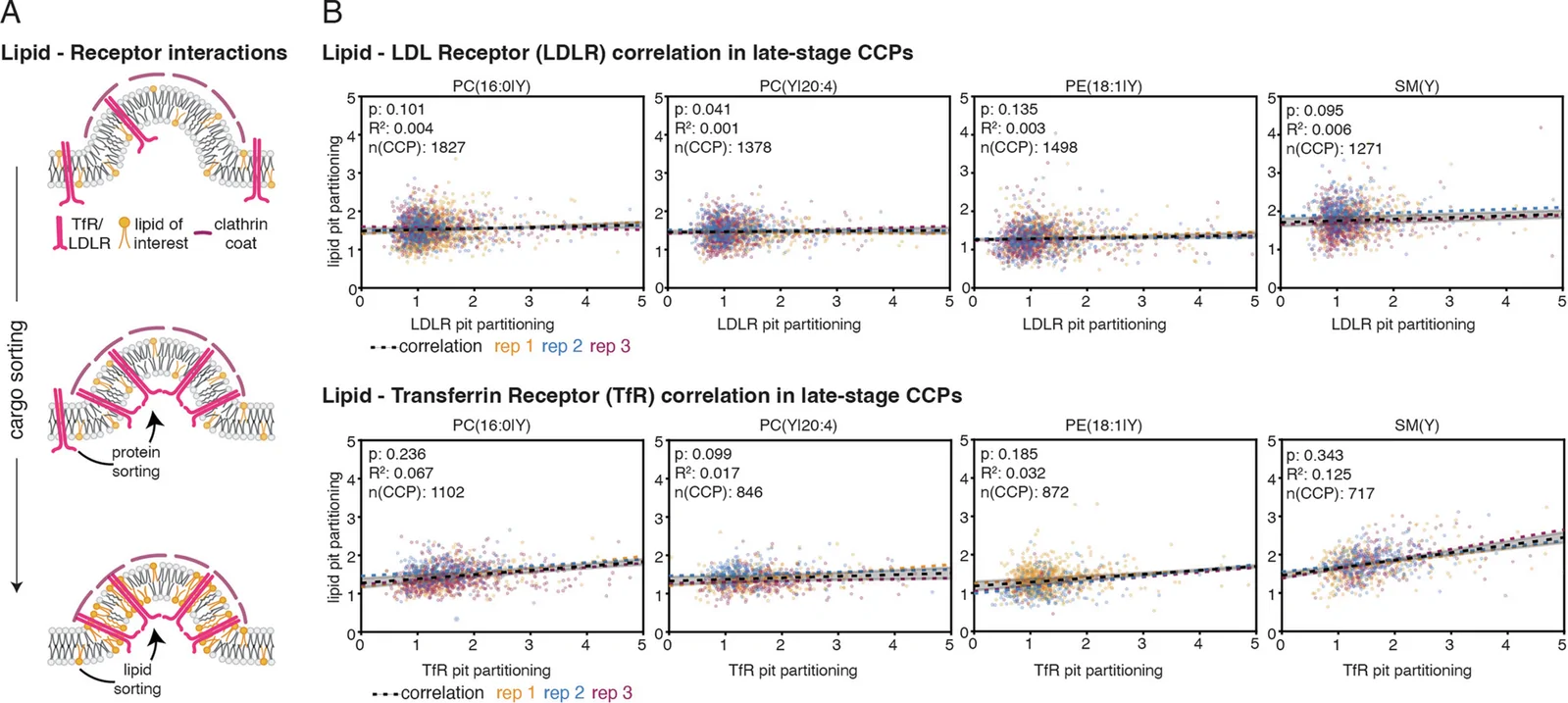
The receptor density in late-stage CCPs does not affect lipid pit partitioning. **a**: Cartoon of possible lipid sorting into CCPs by association with CCP-localized receptors. **b**: The pit partitioning of lipids (PC(16:0|Y), PC(Y|20:4), PE(18:1|Y), or SM(Y)) and receptors (Transferrin receptor (TfR) or LDL receptor (LDLR)) were measured by 3-color STED imaging. Pit partitioning values for lipids and receptors are plotted per single late-stage CCP. Datapoints of each replicate are color-coded as indicated. The correlation was performed using the Spearman’s rank correlation. The correlation coefficients (ρ), the coefficients of determination (R^2^) and number of datapoints (n(CCP)) are indicated per lipid-receptor pair. The mean correlation (black dotted line) and standard deviation (shaded grey area) were determined from the correlations obtained per biological replicate. The experiment was performed in 3 biological replicates. Fields of view per replicate and total clathrin coated pit numbers are provided in the Source Data. Source data

To understand which features could be responsible for the observed moderate yet significant differences in CCP partitioning, we first investigated whether the density of specific transmembrane CCP cargoes (receptors) could affect lipid pit partitioning. Using three-colour STED imaging, we simultaneously measured the pit partitioning of a lipid (PC(16:0|Y), PC(Y|20:4), PE(18:1|Y) or SM(Y)) and a trans-membrane receptor (transferrin receptor (TfR) or LDL receptor (LDLR)). Pit partitioning values of each lipid–receptor pair for late-stage CCPs were correlated using Spearman’s rank correlation (Extended Data Fig. 7). Correlation analysis revealed negligible to moderate correlations for all pairs, with correlation coefficients ranging from 0.041 to 0.343. These data show that moderate increases of lipid pit partitioning due to protein content can be expected for specific protein–lipid species pairs, as shown for SM(Y) and TfR with a moderate positive correlation (*ρ*: 0.343). However, overall, our findings indicate that the protein content of CCPs largely does not affect lipid partitioning for the receptors tested here, and does not explain the differences in lipid CCP partitioning.

**Fig. 4:**
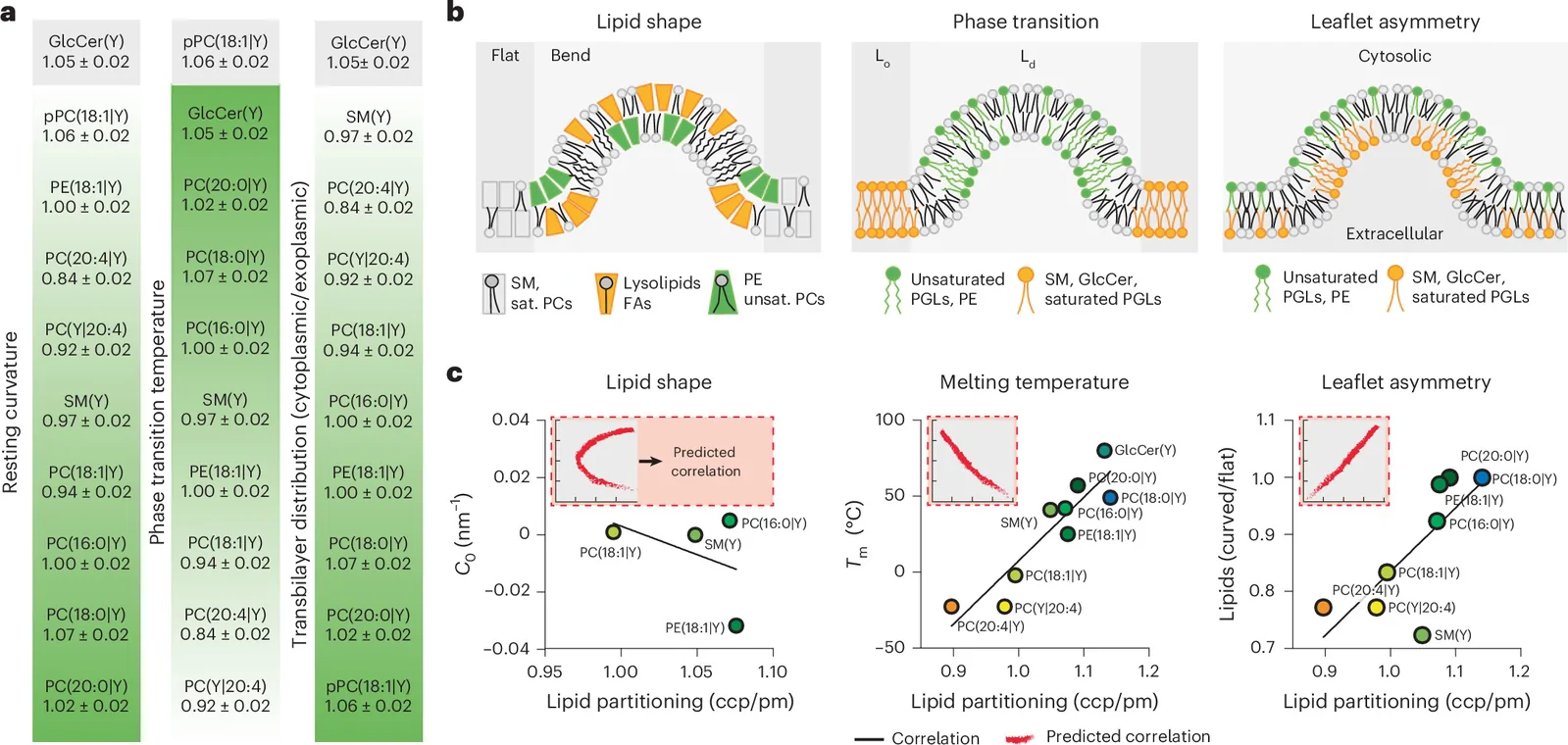
Lipid partitioning into CCPs is consistent with a model of leaflet asymmetry. **a**, Lipids were ordered according to their resting curvature, melting temperature and transbilayer distribution (cytoplasmic/exoplasmic) based on reported values from the literature or estimates based on close structural analogues. Numbers indicate measured pit partitioning with ci95 shown also in Fig. 3b. **b**, Schemes of possible mechanisms of lipid sorting into CCPs are shown. L_d_, liquid disordered phase; L_o_, liquid-ordered phase; sat., saturated; unsat., unsaturated. **c**, The median of absolute lipid densities of late-stage pits is plotted against the respective resting curvature (*C*_0_), melting temperature (*T*_m_) and the ratio of estimated lipids in a vesicle over a flat membrane of the same surface area. The values plotted can be found in Supplementary Data 2–4. Only the lipids are plotted for which the corresponding values of *C*_0_, *T*_m_ and lipid asymmetry were reported in the literature. The red line is a sketch and indicates the expected trend of the correlation according to the model. Numbers of acquired STED images per biological replicate are provided in the source data. Source data

Next, we assessed whether the biophysical properties of our analysed lipids could have an effect on lipid pit partitioning, by applying a similar correlation approach. We considered three possible mechanisms of sorting: partitioning of lipids into curved membranes driven by lipid shape (lipid resting curvature), sorting due to phase separation in curved membranes (lipid melting temperature) and sorting due to the asymmetric trans-bilayer distribution of lipids in the plasma membrane (lipid asymmetry) (Fig. 4a,b). Values of lipid resting curvature, lipid melting temperature and trans-bilayer lipid distribution were obtained from the literature^32–37^ (Supplementary Data 2–4). In cases of missing reference data, values were approximated based on the structurally closest lipid counterparts. These values correspond to the (saturated) native counterparts of the lipid probes used here. The actual values of the bifunctional probes are probably shifted to slightly lower melting temperatures, because the diazirine group acts as one additional double bond (Extended Data Fig. 1)^19^.

First, we considered sorting by lipid shape. According to this hypothesis, cone-shaped (for example, lyso-phosphatidic acid) and inverse cone-shaped lipids (for example, 1,2-dioleoyl-*sn*-glycero-3-phosphoethanolamine) would be sorted into highly curved membranes^38^. Therefore, for the lipid species studied here, we would expect higher partitioning of PE(18:1|Y), GlcCer(Y) and polyunsaturated PCs into CCPs, whereas cylindrical lipids such as SM(Y) and saturated PCs would be excluded. In contrast to this hypothesis, we found that the resting curvatures (*C*_0_) did not correlate with CCP partitioning, suggesting that sorting based on lipid shape is not a plausible mechanism (Fig. 4c). This finding is well in line with several studies showing that lipid shape alone is not sufficient for effective lipid sorting (see the discussion in Supplementary Information)^38,39^.

Next, we assessed local phase separation in curved membranes as a possible mechanism^40–42^. We used the melting temperature (*T*_m_) of the lipid species as a proxy for phase partitioning. According to this hypothesis, highly curved membrane areas should accumulate liquid-disordered (L_d_) lipids owing to the lower bending rigidity of the L_d_ phase^43,44^. This mechanism would result in a negative correlation between lipid *T*_m_ and CCP partitioning. By contrast, we found a strong positive correlation between *T*_m_ and partitioning into CCPs (Fig. 4c). The long-chain saturated PCs, SM and GlcCer with high *T*_m_ are enriched while the (poly)unsaturated lipids with low *T*_m_ are depleted from CCPs. This correlation is surprising as several studies have suggested that liquid-ordered (L_o_) lipids with high *T*_m_ are not trafficked by CME^10,11,45,46^.

Finally, we asked whether the asymmetric distribution of lipids in the plasma membrane impacts partitioning into CCPs. Due to the differences in leaflet surface areas in the curved membrane of the pit and flat surfaces^34^, we expect that lipids enriched in the exoplasmic leaflet should be partially excluded from the CCP, while lipids enriched in the cytoplasmic leaflet should be partially enriched (Fig. 4b). Therefore, we expect a positive correlation between the lipid asymmetry (cytoplasmic/exoplasmic) and CCP partitioning. Indeed, we found a positive correlation for the asymmetry of all PCs and PE, with the exception of SM (Fig. 4c), suggesting that lipid asymmetry is sufficient to explain CCP partitioning for almost all investigated lipids. Taken together, our correlation analysis suggests that lipid asymmetry of the plasma membrane is the major mechanism underlying the limited sorting of lipids into CCPs.

### Lipid asymmetry is required for CME

**Extended Data Fig. 8:**
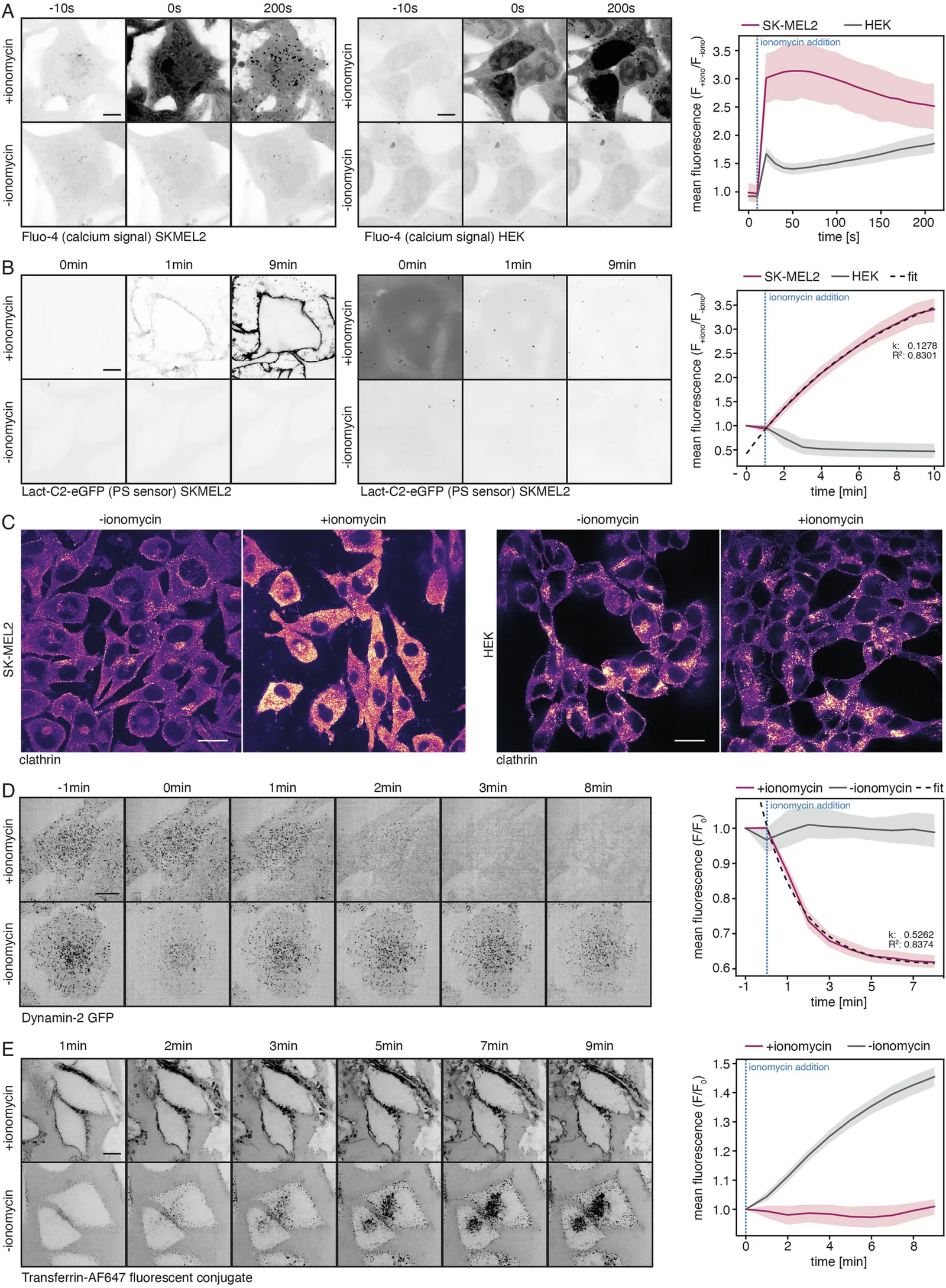
Clathrin-coated pits disassemble upon plasma membrane scrambling. SK-MEL2 or HEK293 cells were treated with 5 µM of the ionophore ionomycin in buffer containing 2.5 mM Ca^2+^. **a**: Relative cytosolic calcium was monitored during life cell imaging for 200 s at 10 s intervals using the Fluo-4 Calcium Imaging Kit (Invitrogen), signal shown in grey. The average Fluo-4 signal of SK-MEL2 (purple) or HEK293 (grey) cells is plotted against time. The signal is normalized to the mean signal of the control (no ionomycin) across time. The error of the normalized average fluorescent signal is shaded and given as the confidence interval (CI) of 95%. Three independent biological samples were analyzed for each condition, n(total fields of view): 18. **b**: Phosphatidylserine (PS) exposure to the extracellular leaflet of the plasma membrane is monitored as a proxy for plasma membrane scrambling. PS exposure is measured using the PS sensor Lact-C2-eGFP^19^ over 10 min at 1 min intervals of SK-MEL2 (purple) or HEK293 (grey) cells. The GFP signal is normalized to the mean signal of the control (no ionomycin) across time. The error of the normalized average fluorescent signal is shaded and given as CI: 95%. Three independent samples were analyzed for each condition, n(total fields of view): 18. The SKMEL trace was fitted (black dotted line) using a simple exponential one phase association model. The rate constant k and R^2^ are indicated. **c**: SK-MEL2 (left panel) or HEK293 (right panel) cells were treated with ionomycin for 5 min in buffer containing 2.5 mM Ca^2+^ and fixed. Clathrin localization was assessed using immunostaining. Representative images of 3 independent biological replicates are shown, that yielded similar results. **d**: Dynamin-2 of a SK-MEL2 dynamin-2-GFP cell line localization was monitored over 10 min at 1 min intervals of cells treated with (purple) or without (grey) ionomycin at the basal plasma membrane. The mean GFP signal is normalized to the mean signal at time −1 min (relative to ionomycin addition). The error of the normalized average fluorescent signal is shaded and given as CI: 95%. Three independent samples were analyzed for each condition, n(total fields of view per condition): 18. The SKMEL trace was fitted (black dotted line) using a simple exponential one phase dissociation model. The rate constant k and R^2^ are indicated. **e**: The uptake of a fluorescently tagged transferrin conjugate was monitored over 10 min at 1 min intervals of cells treated with (purple) or without (grey) ionomycin. The mean fluorescent signal is normalized to the mean signal at time -1 min (relative to ionomycin addition). The error of the normalized average fluorescent signal is shaded and given as CI: 95%. Three independent samples were analyzed for each condition, n(total fields of view): 30. Scale bars: 10 µm. Scale bars of ROIs: 1 µm. Fields of view per replicate are provided in the Source Data. Source data

Despite the experimental challenges associated with perturbing lipid transbilayer composition in living cells, we attempted to investigate the proposed connection between lipid asymmetry and lipid partitioning into CCPs. As genetic perturbations of the flippase machinery that establishes lipid asymmetry (TMEM30A) result in complex remodelling of the lipidome, proteome and metabolism that go far beyond a simple loss of lipid asymmetry^47,48^, we opted for an acute perturbation. To this end, we induced lipid scrambling mediated by the Ca^2+^-dependent scramblase TMEM16F by treating both SK-MEL2 cells, which express TMEM16F and HEK293 cells, which have low endogenous scramblase activity^49,50^, with ionomycin in a calcium-containing buffer. Both cell types responded with robust calcium influx (Extended Data Fig. 8a), but phosphatidylserine (PS) externalization was observed only in SK-MEL2 cells (Extended Data Fig. 8b). Thus, by comparing both cell lines, we wanted to separate the effects of elevated cytosolic Ca^2+^ levels from the scrambling of plasma membrane lipids. We found that following ionomycin treatment, clathrin was rapidly released from the plasma membrane in SK-MEL2 but not in HEK293 cells (Extended Data Fig. 8c). Similarly, the plasma membrane-bound pool of dynamin-2–GFP was fully released 3 min after ionomycin addition. Indeed, late-stage CCPs disassembled with approximately four times faster kinetic rates (rate constant *k*_(dynamin disassembly)_ ± ci95: 0.5262 ± 0.0691) compared with PS externalization (*k*_(PS assembly)_ ± ci95: 0.1278 ± 0.0564) (Extended Data Fig. 8d). In line with these data, we found that ionomycin also fully blocks transferrin uptake in SK-MEL-2 cells (Extended Data Fig. 8e). Thus, experimentally, it was not possible to monitor lipid partitioning into CCPs after plasma membrane scrambling.

Together, our data indicate that the asymmetric lipid composition of the plasma membrane is a prerequisite for CME and that lipid asymmetry and CME are too closely linked to be functionally separated. This finding is in line with previous studies^51^ and the reported role of lipids, such as PI(4,5,)P_2_ and PS, to facilitate the binding of multiple proteins of the CME machinery^51,52^.

### Uptake kinetics of lipid species into endosomes are similar

**Fig. 5:**
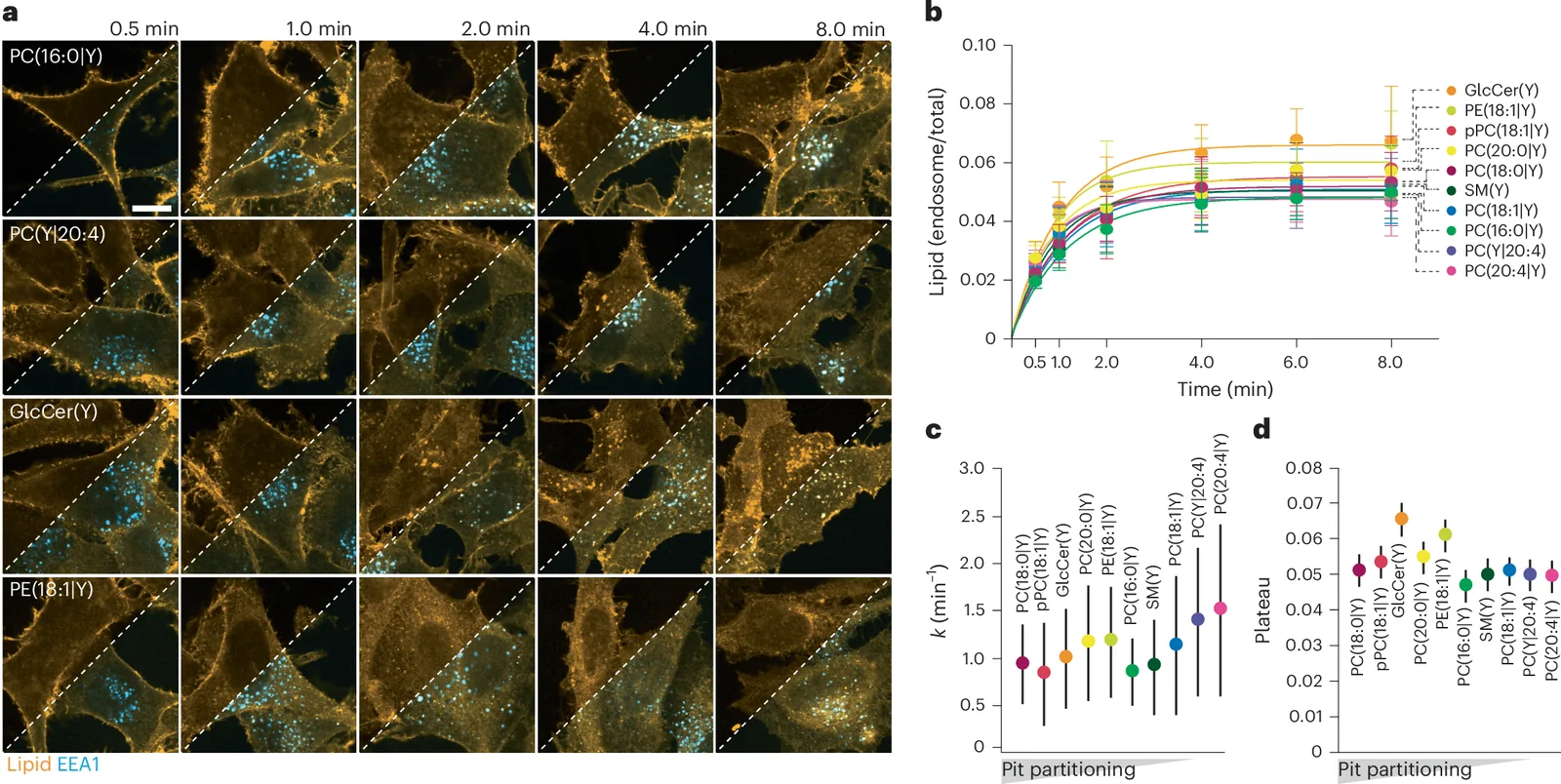
Lipid uptake into early endosomes reveals moderate sorting during endocytosis. **a**, Exemplary fluorescent images of SK-MEL2 wild-type cells loaded with lipid for 0.5 min and chased for 0 min, 0.5 min, 1.5 min, 3.5 min and 7.5 min are shown. The combined pulse and chase times are indicated in the figure. The left half of each image shows the lipid channel only (orange), and the right half the overlay with the EEA1 co-stain (blue). Scale bar, 10 µm. **b**, Relative fluorescent lipid signal in early endosomes over total lipid signal was quantified for 6 different time points and 10 lipids for 3 biological replicates. *N* (fluorescence image per timepoint per lipid), 36. The mean and standard deviation are shown. Uptake traces were fitted to a one-phase association model. The order of lipids from highest to lowest early endosomal amount at 8 min is shown to the right. **c**, The rate constants determined from the fit are plotted with the 95% confidence interval error. Rate constants are ordered from highest to lowest pit partitioning. **d**, The amplitudes of each fit are shown with the 95% confidence interval error indicated. Amplitude values are ordered from highest to lowest pit partitioning. Numbers of acquired fields of view per biological replicate are provided in the source data. Source data

Finally, we assessed whether the magnitude of the observed partitioning differences of lipids into CCPs is sufficient to affect bulk endocytic lipid uptake (Fig. 5).

**Extended Data Fig. 9:**
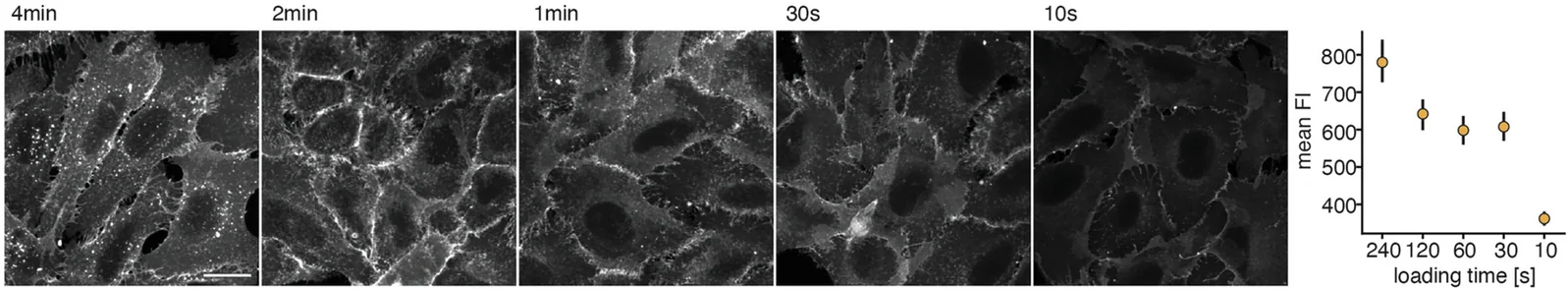
Different lipid loading times. Optimization of lipid loading (pulse) times for high temporal resolution of lipid uptake into early endosomes. U2OS wildtype cells were loaded with bifunctional sphingomyelin for 4 min, 2 min, 1 min, 30 s and 10 s. Mean fluorescent intensities are plotted, error bars indicate the standard deviations. The dataset includes 3 independent technical replicates per timepoint, that yielded similar results. N(images per replicate, per condition): 9. Scale bar in 20 µm.

To this end, lipids were pulsed to SK-MEL2 wild-type cells for 30 s (Extended Data Fig. 9) and chased for defined amounts of time (0 min, 0.5 min, 1.5 min, 3.5 min, 5.5 min or 7.5 min) before cross-linking. The lipid signal in early endosomes was quantified by co-localization with the early endosomal marker EEA1 and normalized to the total lipid signal. This quantification provided temporally highly resolved uptake kinetics of all lipids into early endosomes. To extract apparent rate constants and amplitudes of lipid uptake into early endosomes, we fitted an empirical mono-exponential model (Fig. 5b,c). We found that the rate constants were not substantially different between all tested lipid species with a mean rate constant of 1.09 min^−1^ ± 0.23 min^−1^. In addition, the amplitude of the lipid signal in the early endosome was similar for all lipids, except GlcCer(Y) and PE(18:1|Y) with slightly increased amplitudes (Fig. 5b,d). Visual inspection of the lipid signal for GlcCer(Y) and PE(18:1|Y) revealed faster depletion of the lipid signal at the plasma membrane, which suggests competing non-vesicular lipid transport processes as previously reported^19^. Thus, it is likely that this difference was caused by overlapping lipid signal from the endoplasmic reticulum (ER), or by differential uptake of GlcCer(Y) and PE(18:1|Y) by other non-CME lipid transport routes to the early endosome within the given time frame.

Together, these data imply that vesicular transport of lipid material from the plasma membrane to the early endosome is largely unspecific, in line with our partitioning analysis.

## Discussion

**Extended Data Fig. 10:**
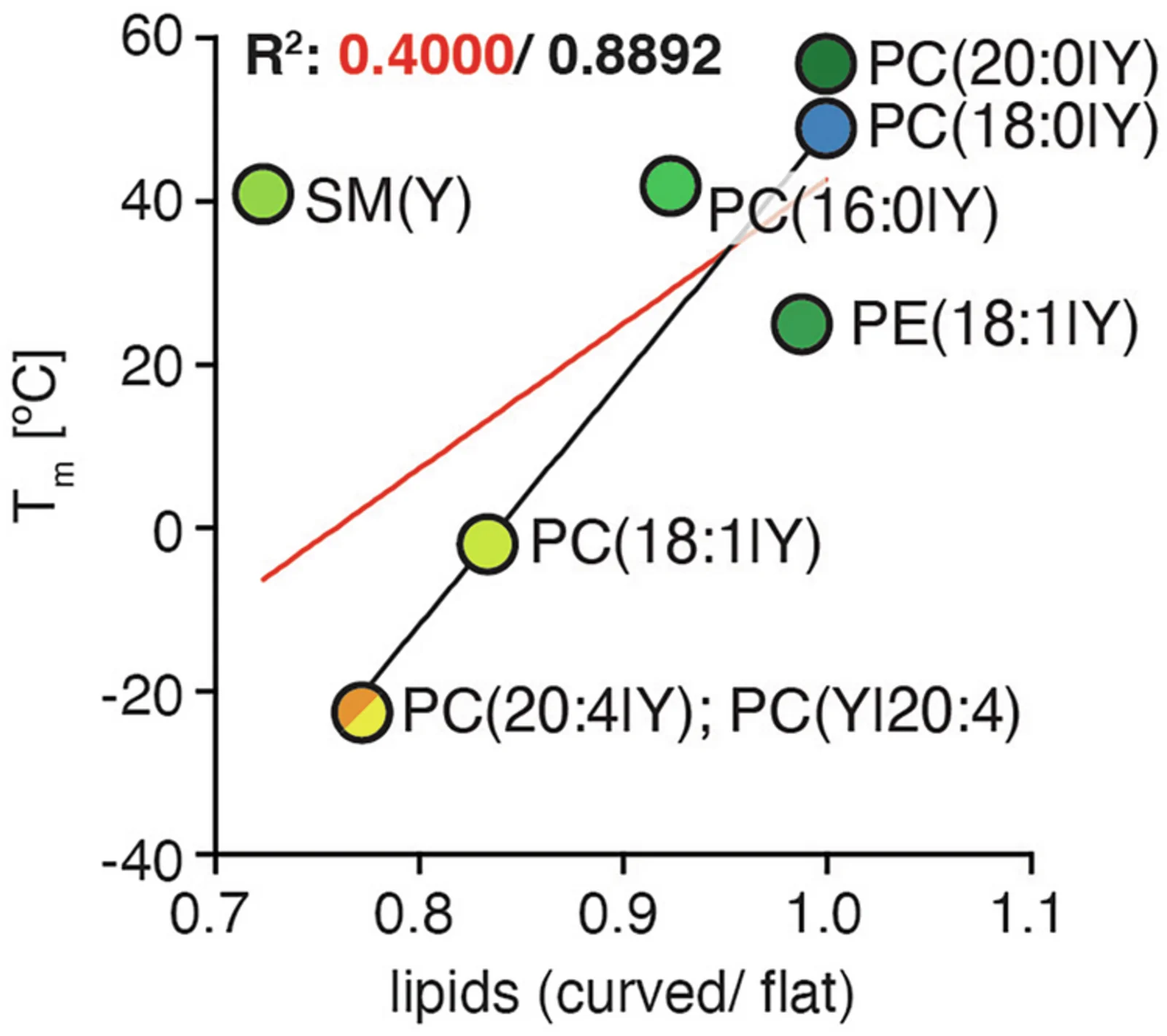
Correlation between lipid asymmetry and melting temperatures. The melting temperatures (T_m_) of different lipid species are plotted against the respective ratio of estimated lipids in a vesicle over a flat membrane of the same surface area. The values plotted can be found in the Supplementary Data 3 and 4. Only the lipids are plotted for which the corresponding values of T_m_ and lipid asymmetry were reported in the literature. The red line indicates the linear regression fit between all lipids, and the black line indicates the same fit but excluding the SM(Y) outlier. The corresponding R^2^ values are indicated on top in red and black, respectively.

Here we introduced super-resolution lipid imaging in combination with mathematical modelling to characterize the extent of lipid sorting during CME. This method allowed us to quantify lipid partitioning into late-stage CCPs. Comparing ten lipid species, we found that lipid partitioning values differed by up to 21% between lipids. A large part of this difference can be explained by the known asymmetric distribution of lipids between plasma membrane leaflets^34^ in combination with pit geometry. In addition, we found a strong correlation between melting temperature and CCP partitioning, with high-melting-temperature lipids partitioning more strongly into CCPs. This is in contrast to the expected depletion of high-melting-temperature lipids into curved membranes, based on previous reports^10,11,45,46^ and the biophysical properties of a curved membrane^43,44^. Thus, it is likely that the observed lipid partitioning trends are not explained by classical L_o_/L_d_ phase separation, which would result in the opposite partitioning behaviour. In this context, it is an intriguing observation that melting temperature and lipid asymmetry are positively correlated, at least for the glycerophospholipids (Extended Data Fig. 10). We have previously observed similar trends for the rates of non-vesicular lipid transport and metabolism for different PC species^19^. The initial step in all three processes is the mobilization and extraction of a lipid from the bulk bilayer by a protein (lipid translocases, lipid transfer proteins, lipid-metabolizing enzymes). It is a compelling speculation that lipid mobilization, which should depend heavily on lipid biophysical properties, could be the rate-limiting step in all cases. If this were the case, lipid biophysical properties would be a key determinant of trans-bilayer, intra- and inter-organelle lipid distribution.

While we cannot exclude other mechanisms, experimentally determined geometric parameters of CCP architecture and lipid-species-specific asymmetries are sufficient and plausible to explain the observed glycerophospholipid partitioning effects. Interestingly, sphingomyelin (SM) deviates from this trend, suggesting other mechanisms, such as specific lipid–protein interactions. Our findings suggest that the lipid composition of clathrin-coated vesicles generated during CME is almost exclusively a reflection of the lipid composition of the plasma membrane and its inherent asymmetric lipid transbilayer distribution. Overall, this implies a slight bias for cytoplasmic leaflet lipids to be transported in the retrograde direction. However, this selectivity is small compared with the differences observed during non-vesicular lipid transport^19,20^. In addition, we previously found that over 80% of lipid transport occurs via non-vesicular lipid transport mechanisms during retrograde transport. This indicates that lipid transport via CME is a minor portion of the total lipid transport volume^19^. Therefore, our data suggest that CME does not substantially affect the steady-state composition of the plasma membrane. By contrast, intracellular organelles will shift to a lipid composition more similar to the plasma membrane by CME, which has to be counteracted by other lipid transport processes such as non-vesicular lipid transport.

Taken together, the selective retrograde transport of lipids and proteins appears to be mostly decoupled. We propose that the main function of CME is to selectively transport proteins, while the associated lipid transport is largely unspecific. However, specific lipid transport is mediated by non-vesicular mechanisms. We note that our findings here do not preclude sorting of individual lipids during clathrin-independent endocytic processes, with caveolae-mediated endocytosis proposed as one possible lipid sorting mechanism^53^, but these mechanisms are probably minor components in the total transport volume of lipids between the plasma membrane and other organelle membranes. Our findings are in line with increasing evidence that lipid sorting during retrograde vesicular trafficking occurs at the level of the endosome as demonstrated for GM1^54^ and SM^24^. We furthermore note that our analysis covered a subset of all lipid species present in the plasma membrane. In particular, we excluded PIP2 and PS because they are well-established regulators of CME, and their inclusion would confound our ability to test whether the CME machinery itself drives the sorting of PIP2 and PS. Consequently, we cannot exclude the possibility that CME selectively regulates the distribution of these specific lipid classes. We anticipate that our methodology for quantifying lipid partitioning into cellular membrane compartments of complex geometry will help in understanding membrane compartmentalization, in particular with regard to lipid transport through the endomembrane system.

## Methods

All research reported in this paper complies with the relevant regulations of the Max Planck Society and the DFG (Deutsche Forschungsgemeinschaft).

### Statistical analysis

#### Sample selection, sample sizes and randomization

No statistical methods were used to predetermine sample sizes, but our sample sizes are similar to or exceed that of previous publications^26^. ROI to acquire STED images were chosen based on two criteria: (1) Selected cells were not blebbing and not detached from the glass surface and (2) selected cells had sufficient AP2–GFP intensity for analysis. For image acquisition by spinning disc fluorescence microscopy, ROIs were chosen automatically by the CellSens software of the microscope used, unless ROIs did not contain cells, in which case ROIs were manually changed to cell-covered areas.

#### Statistical tests

To determine statistical significance between samples of large sample sizes (primarily the numbers of CCPs), we performed a two-sided permutation test to minimize false positives due to sample size.

#### Blinding statement

Data collection analyses were not performed blind to the conditions of the experiment.

#### Data exclusion statement

Images were excluded from analysis if the acquisition process failed owing to the following reasons for fixed cell imaging: cells out of focus and bright cellular debris in the image. For live cell imaging applications, videos were not used if cells moved out of frame or out of focus, thereby precluding quantification. For fixed cell imaging, signal stemming from bright cellular debris was alternatively removed from images either by automatically thresholding images using Python or manually using Fiji. During image analysis, CCPs were excluded if closely adjacent CCPs prevented segmentation as single pits using both a circularity and size threshold in Python.

### LED characterization

LEDs were custom built in-house. The following LEDs were tested for their power output and cross-linking efficiency: a 308-nm LED without a lens (PKF-50, UVB LED, 308 ± 5 nm, 3049140, Laser Components) as previously reported^19^ and a high-power 365-nm LED with (UVB LED 365 ± 10 nm, VC2X2C45L9-365, Violumas) and without a lens (UVB LED, 365 ± 10 nm, VC2X2C45LX-365, Violumas). The power was measured using a PM400 Optical Power Meter from Thor Labs with a S175C Sensor from Thor Labs. The power stated is the maximum power measured over 10 s of exposure time. The power was measured for the wavelength specific for each LED (either 308 nm or 365 nm). Cross-linking efficiency was determined by 10 s, 5 s, 2 s and 1 s of irradiation of 100 mM bifunctional palmitic fatty acid in DMSO solution. UV irradiation was performed in 96-well plates with glass bottoms (Greiner, 655891). Spectra of the bifunctional palmitic fatty acid were measured with a TECAN SPARK 20M in a 96-well black plate with a plastic bottom (Greiner, 655090) and using no lid. Cross-linking efficiency in U2OS wild-type cells was determined by 10 s, 5 s, 2 s and 1 s of irradiation of bifunctional sphingomyelin with the respective LED.

### Cell culture

SK-MEL2 dynamin-2–GFP and SK-MEL2 wild-type cells (gift from D. Drubin, described in a previous study^28^) were cultured in DMEM/F-12 + GlutaMAX (Gibco, 10565-018) medium supplemented with 20% fetal bovine serum and 100 U ml^−1^ penicillin–streptomycin at 37 °C with 5% CO_2_ in 75-cm^2^ flasks. Cells were split every 3–4 days using 0.05% trypsin, ensuring that they never reach a confluency of 100%. Cells were used up to passage 15. For experiments, cells were seeded 24 h before the treatment into fibronectin bovine plasma-coated (Sigma, F4759-2MG) 96-well glass-bottom plates (Greiner, 655891) at a density of 20,000 cells per well. Fibronectin coating was done at 37 °C for 30 min before cell seeding at 10 µg ml^−1^ in phosphate-buffered saline (PBS).

### Liposome preparation and lipid loading

A suspension of 1.5 mM bifunctional lipid, 0.75 mM 1-palmitoyl-2-oleolyl-glycerol-3-phosphocholine (Avanti, 26853-31-6) and 0.75 mM cholesterol (Sigma, CAS: 57-88-5, C8667-5G) in PBS was prepared. Liposomes were made from this suspension using an Avanti Mini-Extruder with 0.1 µm polycarbonate membranes (Avanti, 610005-1Ea), extruding a minimum of 21 times. Liposomes were stored for up to 2 weeks at 4 °C. Liposomes and alpha-methyl-cyclodextrin (Bio-reagent, CDexA-076/BR, CAS: 699020-02-5) were dissolved in serum-free medium to a final concentration of 0.5 mM and 4 mM, respectively. This mix was incubated for at least 30 min at 37 °C before loading on cells. Before lipid loading, cells were gently washed three times with serum-free medium. Lipids were loaded for 4 min at 37 °C for STED imaging and 30 s for imaging by spinning disc microscopy. Cells were washed 3 times in serum-free medium, followed by immediate photoactivation (unless stated otherwise) using a custom-built high-power 365-nm LED (UVB LED, 365 ± 10 nm, VC2X2C45L9-365, Violumas).

### Cell fixation and immunostainings

Samples were fixed immediately after photoactivation for 20 min at room temperature using a fixation buffer of 3% PFA, 10 mM MES, 150 mM NaCl, 5 mM EGTA, 5 mM d glucose and 5 mM MgCl_2_, pH 7.2. Fixed cells were washed 3 times with 100 mM glycine in PBS and permeabilized using 0.1% Triton in PBS for 30 min at room temperature. Fixed and permeabilized samples were blocked at room temperature for 1 h in blocking buffer composed of PBS supplemented with 2% bovine serum albumin (BSA, Sigma, A7030-10G). Primary antibodies were incubated for either 1 h at room temperature or overnight at 4 °C in blocking buffer. Samples were washed 3 times for 15 min in blocking buffer at room temperature and incubated with the respective secondary antibody for 1 h at room temperature in blocking buffer. Samples were washed 3 times for 15 min in blocking buffer at room temperature and kept in PBS. Samples were always immunolabelled before staining by click chemistry. All primary and secondary antibodies used are shown in Supplementary Table 1 of the Supplementary Data file. The following antibody combinations were used for two-colour STED: clathrin light chain–Star Red anti-rabbit and AP2–Star 460L anti-mouse; and for three-colour STED: clathrin light chain–Star 460L anti-rabbit, LDL receptor/transferrin receptor–Star Red anti-mouse and GFP booster.

### Copper-catalysed click reaction

Samples were washed and equilibrated in 100 mM HEPES and stained using copper-catalysed click chemistry by incubation at 37 °C with a click mix of 2 µM AF594 picolyl-azide dye (Jena Bioscience, CLK-1296-1), 0.1 mM CuSO_4_, 5 mM ascorbic azide and 0.5 mM THPTA (Jena Bioscience, CLK-1010-1G) in 100 mM HEPES at pH 7.3. Samples were stained 3 times with fresh click mix for 30 min each.

### Plasma membrane scrambling assay

Before imaging, SK-MEL2 cells were washed and kept in calcium buffer (10 mM HEPES, 140 mM NaCl, 2.5 mM CaCl_2_, pH 7.4). Ionomycin (MP Biomedicals, 159611) was added to a total concentration of 5 µM. Calcium influx was monitored for 3.5 min at 10-s intervals using the Fluo-4 Calcium Imaging Kit (Invitrogen, F10489) according to the manufacturer’s instructions. Plasma membrane scrambling was monitored over 10 min at 1-min intervals using the previously reported phosphatidylserine sensor Lact-C2-eGFP^19^ at 0.5 µM final concentration. Dynamin localization was monitored at the basal cell membrane using the SK-MEL2 dynamin-2–GFP cell line. Transferrin uptake was determined by adding 20 µg ml^−1^ transferrin human serum, Alexa Fluor 647 conjugate (Invitrogen, T23366). Life cell imaging was performed in 96-well glass-bottom plates (Greiner, 655891) at 37 °C with 5% CO_2_ in calcium buffer. For fixed cell experiments, cells were treated with ionomycin for 5 min in calcium buffer before fixation.

### Monitoring changes in membrane microenvironment by Laurdan temperature scans

The following were purchased from Avanti Polar Lipids and used without further purification: 1-palmitoyl-2-oleoyl-glycero-3-phosphocholine (POPC), ᴅ-glucosyl-β-1,1′-*N*-palmitoyl-ᴅ-erythro-sphingosine (GlcCer (18:1/16:0)), 1-stearoyl-2-palmitoyl-*sn*-glycero-3-phosphocholine (PC (18:0/16:0)), 1,2-diarachidoyl-*sn*-glycero-3-phosphocholine (PC (20:0/20:0)) and 1-(1*Z*-octadecenyl)-2-oleoyl-*sn*-glycero-3-phosphocholine (pPC (18/18:1)). The bifunctional lipid derivatives GlcCer(Y), PC(18:0/Y), PC(20:0/Y) and pPC(18:1/Y) were synthesized as described in the section ‘Synthesis of bifunctional lipid probes’. Laurdan was purchased from Thermo Fisher. Polycarbonate membranes (100 nm) were purchased from Whatman. Organic solvents of spectroscopic grade were purchased from Merck. Other chemicals (HEPES, ᴅ-glucose and NaCl) were purchased from Sigma-Aldrich.

#### Preparation of large unilamellar vesicles with Laurdan

The desired amount of lipids dissolved in chloroform was pipetted into a clean glass vial. Laurdan was added to the lipid mixture at a probe/lipid ratio of 1:100. The organic solvent was evaporated from the lipid mixture using a gentle stream of nitrogen gas. To ensure thorough drying, the vial was placed in a vacuum chamber overnight. After overnight drying, the lipid film was rehydrated by adding the desired buffer solution (10 mM HEPES, 10 mM NaCl, and 170 mM glucose buffer, pH 7.4), reaching the final lipid concentration of 1 mM. To ensure proper hydration of the lipid film, the vial was gently vortexed, which resulted in the formation of multilamellar vesicles. To get monodisperse large unilamellar vesicles (LUVs), the solution was extruded through Whatman Nucleopore Track-Etched Membrane filters with a 100-nm pore size by a miniextruder (Avestin). The obtained solution of LUVs was subjected to measurement without any further modifications.

Laurdan temperature scans are based on the measurements of generalized polarization (GP) of Laurdan and were performed on a Lipid State Observer (LISO) device provided under a loan agreement between Augsburg University and the J. Heyrovský Institute. This device enables measurement of GP as a function of time or temperature. For the experiment, 220 μl of the LUV sample was pipetted into Sapphire PCR tubes (Greiner) and subjected to measurement. The sample was excited at 390 nm, and the emission spectra were recorded over the 400–550-nm range as a function of temperature. The integration time was set to 20 ms, and the temperature scan was performed in the range −10 °C to 40 °C at a rate of 0.5 °C min^−1^, with a pre-equilibration time of 20 min. The final GP values were calculated by measuring the intensity of Laurdan at two different wavelengths simultaneously:

$$\mathrm{GP}=\frac{{I}_{{m{B}}}-{I}_{{m{R}}}}{{I}_{{m{B}}}+{I}_{{m{R}}}}$$

where *I*_B_ and *I*_R_ are the fluorescence intensities recorded in the blue region (420–460 nm) and red region (470–510 nm), respectively. Whereas more positive values indicate an increased lipid order or decreased water permeability into the lipid bilayer, more negative values indicate a disordered character of the membrane with increased water permeability.

### Impact of bifunctional lipid derivatives on the organization of ganglioside nanodomains

The following were purchased from Avanti Polar Lipids and used without further purification: 1,2-dioleoyl-*sn*-glycero-3-phosphocholine (DOPC; molecular weight (MW), 814.62), cholesterol (ovine wool; MW, 386.65), *N*-stearoyl-ᴅ-erythro-sphingosylphosphorylcholine (SM; MW, 730.59), GM1 ganglioside (bovine brain sodium salt; MW, 1,546.8), 1-palmitoyl-2-oleoyl-glycero-3-phosphocholine (POPC; MW, 760.08), ᴅ-glucosyl-β-1,1′-*N*-palmitoyl-ᴅ-erythro-sphingosine (GlcCer), 1-stearoyl-2-palmitoyl-*sn*-glycero-3-phosphocholine (PC (18:0/16:0)), 1,2-diarachidoyl-*sn*-glycero-3-phosphocholine (PC (20:0/20:0)) and 1-(1*Z*-octadecenyl)-2-oleoyl-*sn*-glycero-3-phosphocholine (pPC (18/18:1)). The bifunctional lipid derivatives GlcCer(Y), PC(18:0/Y), PC(20:0/Y) and pPC(18:1/Y) were synthesized as described in the section ‘Synthesis of bifunctional lipid probes’. BODIPY-FL-C5-GM1 and BODIPY-564/570-C5-GM1 were synthesized by I. Mikhalyov and N. Gretskaya (Russian Academy of Sciences) as described previously^56^. All lipids were dissolved in chloroform, except for labelled and unlabelled gangliosides, which were dissolved in a mixture of chloroform and methanol in a 2:1 ratio (v/v). Organic solvents of spectroscopic grade were purchased from Merck. Biotin-labelled BSA (biotinyl-BSA) and other chemicals (HEPES, ᴅ-glucose, sucrose, NaCl) were purchased from Sigma-Aldrich. Streptavidin was purchased from Iba Lifesciences.

#### Preparation of GUVs

Giant unilamellar vesicles (GUVs) were obtained by electroformation as previously described^57^. Briefly, lipid films were prepared from chloroform lipid stock solutions deposited on two titanium plates. To ensure complete solvent evaporation, the plates were placed on a heated surface at about 35 °C and then subjected to vacuum for at least 1 h. The lipid-coated plates were assembled and the resulting chamber was filled with sucrose buffer (103 mOsm kg^−1^). The electro-swelling was performed at 47 °C at an alternating electrical field of 10 Hz starting from 0.02 V to 1.1 V (peak to peak) for the initial 45 min, followed by 2 Hz at 1.1 V for 1.5 h. The generated GUVs were transferred to BSA-biotin–streptavidin-precoated imaging chambers (8-well μ-Slide Ibidi) filled with a glucose buffer (~80 mM glucose, 10 mM HEPES and 10 mM NaCl, pH 7.2, 103 mOsm kg^−1^).

#### The approach

The impact of modified lipids on lipid nanodomains in GUVs was determined using the following lipid mixtures: DOPC/Chol/SM (75/25/0) as a control, and nanodomain mix A (65/25/10) or nanodomain mix B (65/25/10 + 4% GM1) both with 5% POPC or with 5% of either GlcCer(Y), PC(18:0/Y), PC(20:0/Y) or pPC(18:1/Y)PC(18:1/Y). Time-resolved fluorescence decays were analysed using BODIPY-FL-C5-GM1 and BODIPY-564/570-C5-GM1 (at the probe-to-lipid ratio of 1:200) as a donor–acceptor pair. Each GUV was imaged at its cross-section with a resolution of 512 × 512 pixels (0.6 ms per pixel), and an experimental donor fluorescence decay was recorded. A minimum of 5 vesicles were analysed per composition, and all measurements were conducted at a temperature of 25 °C. The analysis is outlined in detail in a previous study^58^. It is based on the use of fluorescently labelled gangliosides, specifically Bodipy-FL-GM1 as donors and Bodipy-564/570-GM1 as acceptors, forming a Förster resonance energy transfer (FRET) pair. These molecules tend to accumulate within ganglioside nanodomains, thereby bringing donors and acceptors into close proximity and enhancing FRET efficiency. The presence of nanodomains notably influences the fluorescence de-excitation kinetics of the donors when they are in close vicinity to the acceptors. Previous studies have shown that the shape of the donor decay curve carries valuable information about the average size and total area covered by the nanodomains. Thus, by solely comparing the shapes of the decay curves, insights into whether there have been alterations in nanodomain characteristics can be obtained.

### Spinning disk imaging

Images were acquired on an Olympus IX83 microscope, equipped with a Yokogawa CSU-W1 SoRa unit, an ORCA-Fusion from Hamamatsu and an ORCA-Flash 4.0 V3 digital CMOS camera. A ×100 immersion oil objective (Olympus UApoN OTIRF) was used for imaging. A 488-nm laser line was used to image EEA1 at 10% power, and a 561-nm laser line was used for the lipid at 50% laser power with their respective filter sets. All images for the lipid transport dataset were acquired using identical settings. Cells were imaged using 17-slice z-stacks, with a distance of 0.5 μm in *z* between frames. To ensure that all images were acquired at the same starting plane, the Olympus TruFocus Z-drift compensation system was used. Each frame was exposed for 100 ms. The pixel size was 65 nm.

### STED microscopy

Images were acquired using a commercial confocal infinity-line STED microscope (Abberior Instruments) equipped with a pulsed laser excitation (640 nm, 560 nm, 460 nm, 40 MHz), beam scanning module (line frequency, 3 kHz) and single-photon-counting avalanche photodiode detectors. An Olympus ×100 oil-immersion objective with a numerical aperture of 1.49 was used. A 775-nm, 40-MHz pulsed laser (Katana HP, 3 W, 1 ns pulse duration, NKT Photonics; MPB, 3 W, 1 ns pulse duration) was applied for the depletion of the 560 nm and 640 nm channel. A pinhole size of 60 µm was used. Images were acquired at a pixel size of 30 nm. The star red dye, AF594, star L460 and GFP were excited at 640 nm, 561 nm, 488 nm and 488 nm and detected in a range of 685 nm | 70 nm, 605 nm | 50 nm and 525 nm | 25 nm, respectively. The system was operated using the Imspector software (v.16.2.8415).

### FRET imaging

Fluorescence lifetime imaging microscopy FRET measurements were conducted using a custom-built set-up comprising an inverted confocal microscope body (IX71, Olympus) and a pulsed diode laser (LDH-P-C-470, 470 nm, PicoQuant) operating at a 10-MHz repetition rate. The laser intensity was maintained at 2 μW to prevent pile-up effects, and it was coupled to a polarization-maintaining single-mode optical fibre before being re-collimated at the output using an air space objective (UPLSAPO 4x, Olympus). The light was then directed onto a water immersion objective (UPLSAPO 60x, Olympus) via a 470/635 dichroic mirror. Donor emission signals were isolated using a 515/50 bandpass filter (Chroma) and detected using a single-photon avalanche diode.

### Image analysis

Image and data post-processing was performed using Python^59^, Ilastik^60^ and ImageJ/Fiji^61,62^. Python version 3.8, ImageJ/Fiji version 2.14.0/1.54f and Ilastik version 1.4.0-OSX were used.

### Linear demixing

The signal from the AlexaFluor594 fluorophore showed bleed-through into the long Stokes channel and was corrected by linear demixing. The correction factor was determined from negative control samples stained only with AlexaFluor594 by division of the long stokes shift convocal signal (bleed-through) over the 594 confocal signal using Fiji^61^. Subsequently, both the confocal image of the 594 channel and the confocal long stokes shift channel were blurred by applying a Gaussian filter. The product of the correction factor and the blurred 594 images were subtracted from the respective blurred long stokes shift images using the self-written script 240724_linear_demixing.py.

### STED image segmentation analysis

The linear demixed confocal AP2 images and confocal dynamin-2 images are segmented using conventional thresholding (threshold yen, skimage). The STED clathrin images were segmented by upsizing images fivefold (cubic interpolation, resize, cv2), rescaling intensity values from 0 to 1, and segmenting (threshold otsu, clesperanto) and labelling (voronoi otsu labelling, clesperanto) using napari. Label images were downsized to the original dimensions (nearest interpolation, resize, cv2). Next, the raw STED lipid images were background corrected. Using the clathrin labels and the background-corrected lipid images, the mean fluorescence lipid signals, area and centroid coordinates were determined per pit (regionprops.measure, skimage). Segmented clathrin pits were assigned as unidentified structures if they did not overlap with both the AP2 and dynamin-2 masks. Pits overlapping with only AP2 were classified as early-stage pits, and pits overlapping with both masks as late-stage pits. Pits below a size of 5 pixels were considered as noise and filtered out. Next, using the centroid coordinates of each pit, a square of 1.5 µm × 1.5 µm (referred to as ROI) was defined around this centre. The mean lipid fluorescent signal of each ROI was determined from background-corrected lipid images in which the areas without cells, high-intensity filopodia and regions of clathrin masks were set to ‘nan’ to be excluded from the mean intensity of each ROI. The pit lipid enrichment was calculated by division of the fluorescent mean pit/mean ROI. For each lipid type and each pit class, the mean pit lipid enrichment was calculated with the respective standard deviations and total number of analysed pits. The significance values between different lipid mean pit lipid enrichment per pit class were calculated using a two-sided randomized test (https://rasbt.github.io/mlxtend/user_guide/evaluate/permutation_test/). Clathrin pit area densities were determined for each pit class per image by taking the ratio of the area (in pixel number) for all pits over the total cell area (in pixel number). Clathrin pit number densities were determined for each pit class per image by taking the ratio of the number of all pits normalized over the total cell area (in pixel number). Data was plotted using Seaborn. The code can be found in the 240724_CCP_segmentation_analysis.py file.

### Ilastik models

We used the two-stage Autocontext pixel classification workflow of Ilastik^60^ for segmenting early endosomes co-stained with EEA1 in the 488 channel. The training was performed in 2D and using different frames of the z-stack.

### EEA1 segmentation and analysis

Image z-stacks were analysed by single images. The EEA1 label was segmented using the Ilastik script EEA1_SKMELwt.ilp. Lipid images were background corrected using the mean fluorescent value of the no-lipid control. The total fluorescent lipid intensity counts were determined per image, not counting cell-free areas. The fluorescent lipid intensity counts were determined for the pixels overlapping with the EEA1 mask (EEA1 intensity counts). Per image z-stack, the sum of the total lipid intensity counts and the EEA1 intensity counts was taken. The ratio of the EEA1 intensity counts over the total lipid intensity counts per image z-stack was considered the total lipid amount in early endosomes in %. This analysis was performed using the self-written python script 270724_EEA1_SKMEL_segmentation.py.

### CCP modelling

Pit geometry modelling was performed by sampling points on a simplified pit geometry. We assumed uniform point distribution on the pit and the membrane surrounding it with point density *ρ*. We used a cut sphere to represent the pit and a catenoid to represent the neck. The radius and opening angle of the cut sphere, along with the height and width of the catenoid, defined the pit geometry. Electron microscopy measurements of CCPs from a previous study^30^ were used to generate biologically accurate pit shapes. We sampled ${n}_{\mathrm{pit}}\sim {\mathscr{N}}\left({N}_{\mathrm{pit}},\,0.1{N}_{\mathrm{pit}}\right)$ points on the cut sphere and the catenoid, where *N*_pit_ = *A*_pit_ × *ρ* and *A*_pit_ is the pit’s surface area. Points were allocated between the cut sphere and the catenoid based on their relative surface areas. Standard uniform sampling on a sphere was used on the cut sphere, while a geometrical sampling algorithm developed previously^63^ was used for the catenoid. We simulated 1,000 early-stage pits and 1,000 late-stage pits using the pit angle distribution from a previous study^26^ to achieve the proper shape distribution of the pits. Owing to observed inconsistencies between the pit and neck parameters in a small subset of electron microscopy data, we needed to exclude certain generated pits from our analysis. We used a threshold of *δ* = 1 nm on the difference between the spherical cap opening radius *r*_s_ and the catenoid opening at the reported height *r*_c_ as defined in equation (1). Any pit with Δ*r* > *δ* is excluded from the analysis. Our threshold excludes (percentage of pits by the chosen delta) of the pits.

1 $${\Delta }r=|{r}_{{m{s}}}-{r}_{{m{c}}}|$$

### STED image simulation and segmentation

Using the coordinates of the simulated point emitters from the CCP modelling, STED images were simulated with a similar pixel size of 30 nm, 30 nm and 500 nm (*x*, *y*, *z*) as the experimental imaging data. The size of the STED microscope PSF was determined by imaging 20-nm Crimson beads and measuring the full width at half maximum in the *XYZ* axis. Simulated point emitters were convolved with a Gaussian PSF with full widths at half maximum of 80 nm, 80 nm and 500 nm (*x*, *y*, *z*) according to the experimentally determined PSF parameters using custom Python code to generate STED images of the 3D models. The generated images were cropped to a 50 pixel × 50 pixel array to remove an edge effect of lower intensities at the image edges caused by the convolution. For the segmentation of the simulated images, a line intensity profile of the experimental data was generated for all early- and late-stage pits. The profile was taken through the centroid of the clathrin mask, reading out both the lipid signal intensity and mask intensity. From the individual profiles, we determined the average width of the mask for early- and late-stage pits, as well as the respective average intensity profile of the lipid signal using the Python script CCP_average_lineprofile_all_pits.py. Average intensity profiles were normalized from 0 to 1, and the normalized intensity cut-off for segmentation in % was determined using the average segmentation mask width. The resulting cut-off was used on the intensity profiles of the individual simulated images to determine individual widths of segmentation masks for the model. Masks were generated as symmetric circles. The ratio between the mean fluorescent intensity within the mask and the mean intensity outside the mask was defined as the apparent partitioning factor. The image simulation and analysis of simulated images was done using the custom python codes Simulate_Pit_STEDImages_early.py and Simulate_Pit_STEDImages_late.py.

### Correlations and exponential fits

The linear correlations and one-phase association exponential fits were performed using GraphPad Prism version 10.4.0 for macOS, GraphPad Software (www.graphpad.com). For linear correlation analysis, values of melting temperatures, resting curvature and lipid asymmetry were determined from the literature^20,33–35,37,44,64–70^ (Supplementary Data), and these values were correlated against the experimentally determined pit partitioning values. For this analysis, we assume that PC(20:4|Y) and PC(Y|20:4) have the same values for *T*_m_, resting curvature and lipid asymmetry. For the lipid asymmetry, we assume that PC(20:0|Y) is localized fully to the cytosolic side based on the reported values of PC(18:0|Y). We estimated the melting temperature of PC(20:0|Y) based on the values of PC(16:0|Y) and PC(18:0|Y). For pPC(18:1|Y) and GlcCer(Y), no literature values were found, and thus, these lipids were excluded from the correlation analysis. For the one-phase association, exponential fits of the values for the mean lipid amount in early endosomes were determined for all lipids and all time points as discussed in the EEA1 segmentation and analysis. We assumed that at time point 0 min, no bifunctional lipid content is localized in the early endosomes. Following a nonlinear regression (curve fit) for one-phase association was performed to determine the rate constants. To determine the values of amplitudes, we performed the same fit with the constraint that the rate constant should be the same for all traces.

### Synthesis and characterization of new chemical probes

All chemicals were obtained from commercial sources (Acros, Sigma-Aldrich, TCI Chemicals, Avanti Polar Lipids, Alfa Aesar, Roth, Fluka or Merck) and were used without further purification. Solvents for flash chromatography were obtained from VWR and Penta Chemicals and dry solvents from Sigma. Deuterated solvents were obtained from Deutero. TLC was performed on precoated plates of silica gel (Merck, 60 F254) using UV light (254 nm or 365 nm) or staining solution of phosphomolybdic acid in EtOH (3 g phosphomolybdic acid in 100 ml EtOH) for analysis. Flash column chromatography was performed using silica gel from Merck (silica 60; grain size, 0.040–0.063 mm) with a pressure of 1 bar. ^1^H-, ^13^C- and ^31^P-NMR spectra were measured on a 400 MHz Advance III HD Nanobay Bruker spectrometer. The chemical shift of ^1^H- and ^13^C-NMR spectra is referenced indirectly to tetramethylsilane. *J* values are given in Hz and chemical shifts in ppm. Splitting patterns are mentioned as follows: s, singlet; d, doublet; t, triplet; q, quartet; m, multiplet; and m_c_, centred multiplet. ^13^C-NMR spectra were broadband hydrogen decoupled. Mass spectra (ESI) were acquired using a QExactive instrument (Thermo Fisher Scientific) equipped with a robotic nanoflow ion source.

### Synthesis of bifunctional lipid probes

SM(Y), PE(18:1|Y), PC(16:0|Y), PC(18:1|Y), PC(20:4|Y), PC(Y|20:4) and the C16 bifunctional fatty acid have been synthesized according to recently published protocols^19,20^. The analytical data were in accordance with those reported. All other protocols and analytical data are listed below.

### Synthesis of PC(18:0|Y_16_)

**Figure:**
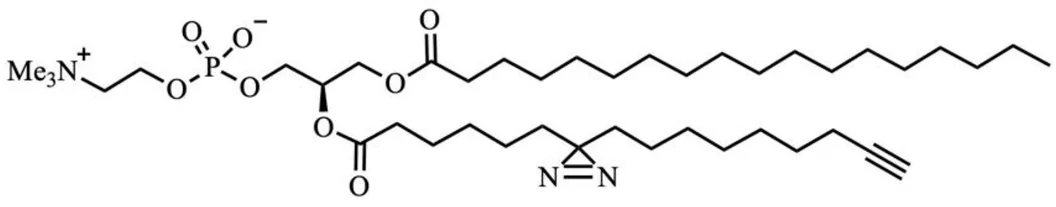

A solution of 31.0 mg of the bifunctional fatty acid C16 (111 µmol, 1.2 eq.) in 1 ml CDCl_3_ was treated with 40.0 mg EDC·HCl (209 µmol, 2.2 eq.) and 5.5 mg DMAP (45 µmol, 0.5 eq.). After 10 min, the solution of the activated fatty acid was added to a solution of 48.6 mg Lyso PC 18:0 (92.8 µmol, 1.0 eq.) in 2 ml dry DMF and 1 ml CDCl_3_. The reaction mixture was stirred overnight at room temperature. After the solvents were removed under reduced pressure, the product was purified by flash chromatography (CHCl_3_/MeOH/H_2_O 70:30:2). PC(18:0/Y_16_) was isolated as a yellowish oil.

^1^H-NMR (400 MHz, CD_3_OD) *δ* = 5.24 (m_c_, 1H), 4.44 (dd, *J* = 12.0, 3.2 Hz, 1H), 4.34–4.23 (m, 2H), 4.17 (dd, *J* = 12.1, 6.8 Hz, 1H), 4.00 (t, *J* = 6.1 Hz, 2H), 3.70–3.59 (m, 2H), 3.23 (s, 9H), 2.33 (t, *J* = 3.3 Hz, 2H), 2.32 (t, *J* = 3.3 Hz, 2H), 2.21–2.10 (m, 3H), 1.54 (dq, *J* = 39.3, 7.2 Hz, 6H), 1.44–1.22 (m, 40H), 1.18–1.04 (m, 4H) and 0.90 (t, *J* = 6.6 Hz, 3H) ppm (triplets at 2.32 and 2.33 ppm overlap).

^13^C-NMR{^1^H} (101 MHz, CD_3_OD) *δ* = 174.93, 174.43, 85.01, 71.83, 69.43, 67.49, 64.91, 63.62, 60.49, 54.69, 34.89, 33.84, 33.74, 33.09, 30.81, 30.78, 30.66, 30.49, 30.20, 29.98, 29.69, 29.64, 29.51, 26.02, 25.76, 25.74, 24.88, 24.67, 23.75, 19.00 and 14.46 ppm.

^31^P-NMR{^1^H} (162 MHz, CD_3_OD) *δ* = −0.56 ppm.

HR-MS (ESI positive) *m*/*z* calculated for C_42_H_78_N_3_O_8_P: 783.5527; found: 784.558 [M + H]^+^.

Yield: 13.4 mg (17.1 µmol, 18%).

### Synthesis of PC(20:0/Y_16_)

**Figure:**
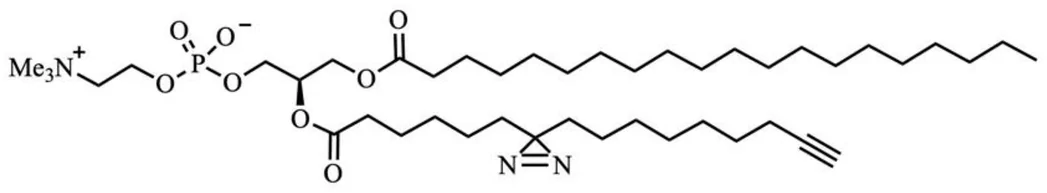

A solution of 31.0 mg of the bifunctional fatty acid C16 (111 µmol, 1.3 eq.) in 1 ml CDCl_3_ was treated with 40.0 mg EDC·HCl (209 µmol, 2.4 eq.) and 5.5 mg DMAP (45.0 µmol, 0.5 eq.). After 10 min, the solution of the activated fatty acid was added to a solution of 48.3 mg Lyso PC 20:0 (87.4 µmol, 1.0 eq.) in 2 ml dry DMF and 1 ml CDCl_3_. The reaction mixture was stirred overnight at room temperature. After the solvents were removed under reduced pressure, the product was purified by flash chromatography (CHCl_3_/MeOH/H_2_O 70:30:2). PC(20:0/Y_16_) was isolated as a yellowish oil.

^1^H-NMR (400 MHz, CD_3_OD) *δ* = 5.24 (s, 1H), 4.44 (dd, *J* = 12.0, 3.2 Hz, 1H), 4.27 (s, 2H), 4.17 (dd, *J* = 12.1, 6.8 Hz, 1H), 4.00 (t, *J* = 6.0 Hz, 2H), 3.64 (t, *J* = 4.6 Hz, 2H), 3.23 (s, 9H), 2.33 (dq, *J* = 9.4, 6.6 Hz, 4H), 2.21–2.11 (m, 3H), 1.54 (dq, *J* = 39.7, 7.1 Hz, 7H), 1.30 (d, *J* = 6.1 Hz, 49H), 1.11 (d, *J* = 10.1 Hz, 4H) and 0.90 (t, *J* = 6.6 Hz, 5H) ppm. ^13^C-NMR{^1^H} (101 MHz, CD_3_OD) *δ* = 174.92, 174.43, 85.01, 71.94, 69.43, 67.45, 64.92, 63.62, 60.50, 54.68, 34.90, 33.84, 33.74, 33.09, 30.79, 30.49, 30.20, 29.98, 29.64, 26.02, 25.77, 24.88, 24.67, 23.75, 18.99 and 14.45 ppm (signals between 29.5 and 30.8 ppm overlap and do not resolve individually).

^31^P-NMR{^1^H} (162 MHz, CD_3_OD) *δ* = −0.56 ppm.

HR-MS (ESI positive) *m*/*z* calculated for C_44_H_82_N_3_O_8_P: 811.5840; found: 812.590 [M + H]^+^.

Yield: 5.1 mg (6.4 µmol, 7%).

Synthesis of 13-oxohexadec-17-ynoic acid

**Figure:**
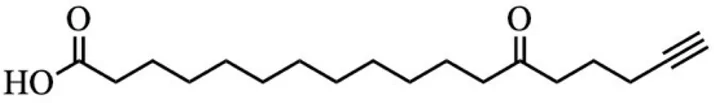

A solution of 5-hexynoic acid (5.0 g, 44.6 mmol, 1.0 eq.) in 40 ml dry DCM was treated with 6 drops of dry DMF. Subsequently, oxalyl chloride (3.4 ml, 51 mmol, 1.1 eq.) was added dropwise at 0 °C under an argon atmosphere. The reaction mixture was stirred for 20 min at 0 °C followed by 60 min at room temperature. A solution of 1-morpholinocyclododecene (15.0 g, 59.7 mmol, 1.3 eq.) in 20 ml dry DCM was heated to 35 °C in an open flask. After the addition of NEt_3_ (11 ml,78.9 mmol, 1.8 eq.) the acyl choride mixture was added dropwise. The reaction mixture was stirred overnight at room temperature. Then, 75 ml chloroform and aqueous HCl (50 ml, 20% v/v) were added, and the mixture was stirred at room temperature for 4 h. The phases were separated and the organic phase was washed with water (3×). The combined aqueous layers were re-extracted with DCM (3×), and the organic phases were then combined and their solvent removed under reduced pressure. The crude product was purified by flash chromatography (CyHex/EtOAc, 97:3). Product-containing fractions were evaporated under reduced pressure and directly used in the next step. The crude intermediate (∼10 g) was heated to 100 °C and treated with aqueous KOH (70 ml, 60% v/v). After stirring for 15 min at 100 °C, the reaction mixture was cooled to room temperature for 60 min. The reaction mixture was diluted with 200 ml water and slowly neutralized through dropwise addition of concentrated aqueous HCl (20 ml). Upon neutralization, a colourless precipitate of crude product appeared, which was collected by filtration. Further crude product was obtained by extracting the remaining water phase with chloroform (3×), combining the organic layers and removing the solvent under reduced pressure. The crude product was purified by flash chromatography (DCM/MeOH, 97:3 to 95:5). 13-Oxohexadec-17-ynoic acid was isolated as a white solid.

^1^H-NMR (400 MHz, CDCl_3_) *δ* = 2.55 (t, *J* = 7.3 Hz, 2H), 2.37 (dt, *J* = 23.6, 7.5 Hz, 4H), 2.28–2.17 (m, 2H), 1.99–1.91 (m, 1H), 1.86–1.72 (m, 2H), 1.70–1.49 (m, 4H) and 1.40–1.17 (m, 15H) ppm.

^13^C-NMR{^1^H} (101 MHz, CDCl_3_) *δ* = 210.91, 179.26, 83.80, 69.11, 43.16, 41.17, 34.02, 29.61, 29.51, 29.36, 29.33, 29.17, 24.82, 24.02, 22.40 and 17.93 ppm (signals between 29.2 and 29.9 ppm overlap and do not resolve individually).

HR-MS (ESI negative) *m*/*z* calculated for C_18_H_30_O_3_: 294.2195; found: 293.213 [M − H]^−^.

Yield: 2.2 g (8.3 mmol, 18 % over 2 steps).

### Synthesis of bifunctional fatty acid Y_18_

**Figure:**
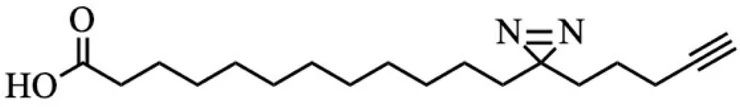

In a 250-ml flask, molecular sieves (3 Å, powder) were dried with heat under high vacuum. Ammonia (20 ml, 7 N, solution in MeOH) was then added, and a solution of 13-oxohexadec-17-ynoic acid (2.0 g, 7.5 mmol, 1.0 eq.) in 25 ml dry MeOH was added dropwise. The reaction mixture was stirred at room temperature for 3 h. Subsequently, a solution of hydroxylamine-*O*-sulfonic acid (1.80 g, 15.9 mmol, 2.1 eq., dried under high vacuum for 3 h) was dissolved in 15 ml dry MeOH and the solution was added dropwise at 0 °C to the reaction mixture. The reaction mixture was stirred at room temperature overnight. The molecular sieves were removed by filtration, and the filtrate was concentrated under reduced pressure. The residue was redissolved in 150 ml MeOH. Then, 6 ml NEt_3_ was added dropwise at 0 °C. Subsequently, iodine was added in small batches at 0 °C until the mixture remained brownish. The reaction was quenched with saturated aqueous Na_2_S_2_O_3_ solution. The reaction mixture was extracted with DCM (3×), the combined organic layers were dried over Na_2_SO_4_ and the solvent was removed under reduced pressure. The crude product was purified by flash chromatography (DCM/MeOH 97:3). The product-containing fraction was purified further by HPLC using a Macherey Nagel VP 250/32 Nucleodur C18 HTec column at 30 ml min^−1^ eluting with a gradient. The solvent system used was A (75% MeOH, 25% water, plus 4% AcOH) and B (50% MeCN, 40% i-propanol, 10% MeOH, plus 4% AcOH). Gradient: 0–5 min, 0–45% B; 5–15 min, 45–75% B; 15–20 min, 75–100% B; 20–27 min, 100–0% B; 27–30 min, 0% B. The retention time of the product was found to be 17.2 min. The bifunctional fatty acid was isolated as a light yellowish oil.

^1^H-NMR (400 MHz, CDCl_3_) *δ* = 2.35 (t, *J* = 7.5 Hz, 2H), 2.16 (td, *J* = 7.0, 2.7 Hz, 2H), 1.97–1.91 (m, 1H), 1.71–1.58 (m, 2H), 1.55–1.44 (m, 2H), 1.42–1.15 (m, 18H) and 1.15–0.99 (m, 2H) ppm.

^13^C-NMR{^1^H} (101 MHz, CDCl_3_) *δ* = 178.14, 83.63, 69.00, 33.81, 33.02, 32.00, 29.63, 29.57, 29.51, 29.35, 29.19, 28.60, 24.83, 23.97, 22.91 and 18.12 ppm.

HR-MS (ESI negative) *m*/*z* calculated for C_18_H_30_N_2_O_2_: 306.2307; found: 305.224 [M − H]^−^.

Yield: 135.0 mg (440.0 μmol, 6 %)

### Synthesis of GlcCer(Y)

**Figure:**
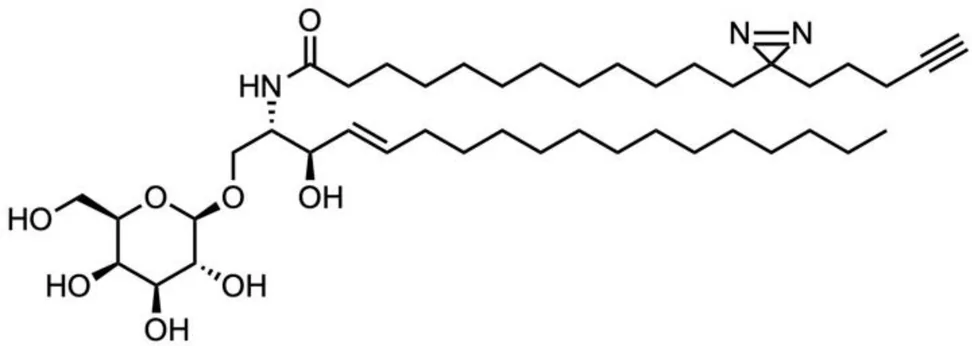

Of the C18 bifunctional fatty acid, 4.3 mg (14 µmol, 1.1 eq.) was mixed with 5.9 mg (12.8 µmol, 1.0 eq.) of ᴅ-glucosyl-β1-1′-ᴅ-erythro-sphingosine and 5.1 mg (33.3 µmol, 2.6 eq.) of 1-hydroxybenzotriazole hydrate. The solids were dried over high vacuum and dissolved in 2 ml of dry DCM and 1 ml of dry THF. The reaction mixture was cooled down to 0 °C, and *N*-(3-dimethylaminopropyl)-*N*′-ethylcarbodiimide (4.5 µl, 25.4 µmol, 2 eq.) was added. The reaction mixture was stirred under an argon atmosphere at room temperature for 48 h. The solvents were removed under reduced pressure, and the crude product was purified with flash chromatography (CHCl_3_/MeOH 20:1 to 10:1). The product GlcCer(Y) was isolated as a white solid.

^1^H-NMR (400 MHz, CD_3_OD) *δ* = 7.83 (t, *J* = 7.9 Hz, 1H), 7.69 (t, 1H), 7.56–7.38 (m, 2H), 5.73–5.57 (m, 1H), 5.48–5.33 (m, 1H), 4.24–4.10 (m, 2H), 4.09–3.99 (m, 1H), 3.98–3.87 (m, 1H), 3.83–3.74 (m, 1H), 3.74–3.61 (m, 2H), 3.62–3.39 (m, 5H), 2.23–2.06 (m, 5H), 2.05–1.92 (m, 2H), 1.62–1.49 (m, 3H), 1.49–1.39 (m, 3H), 1.39–1.15 (m, 43H), 1.13–0.99 (m, 3H) and 0.92–0.78 (m, 4H) ppm.

^13^C-NMR{^1^H} (101 MHz, CD_3_OD) *δ* = 175.97, 134.97, 131.36, 129.57, 128.26, 127.12, 118.69, 111.45, 105.39, 84.12, 76.78, 74.86, 72.99, 72.65, 70.32, 70.05, 69.96, 62.53, 54.82, 37.38, 33.82, 33.46, 33.10, 32.79, 30.85, 30.81, 30.75, 30.66, 30.61, 30.55, 30.50, 30.43, 30.32, 29.21, 27.15, 24.91, 24.01, 23.76, 18.56 and 14.46 ppm.

HR-MS (ESI positive) *m*/*z* calculated for C_42_H_75_N_3_O_8_: 749.5554; found: 750.561 [M + H]^+^.

Yield: 9.0 mg (12.0 µmol, 94%).

### Reporting summary

Further information on research design is available in the Nature Portfolio Reporting Summary linked to this article.

## Online content

Any methods, additional references, Nature Portfolio reporting summaries, source data, extended data, supplementary information, acknowledgements, peer review information; details of author contributions and competing interests; and statements of data and code availability are available at https://doi.org/10.1038/s41556-026-02068-7.

## Supplementary information

Supplementary Information Supplementary Figs. 1–12 and description of Supplementary Data.

Reporting Summary

Peer Review File

Supplementary Data Supplementary data files.

## Source data

Source Data Figs. 1–5, and Extended Data Figs. 1–5 and 7–10 Numerical source data.

## Data Availability

The data that support the findings of the study are available via the EDMOND repository of the Max Planck Society at https://doi.org/10.17617/3.GLIVDU and upon request from the corresponding authors. Source data are provided with this paper. All other data supporting the findings of this study are available from the corresponding authors on reasonable request.

## Code availability

The software code used for analysis is available via the EDMOND repository of the Max Planck Society at https://doi.org/10.17617/3.GLIVDU and upon request from the corresponding authors.
