# Supplementary material for: Quantification of lipid sorting during clathrin-mediated endocytosis
Source: Nat Cell Biol. 2026 Sep 29;28(10):2027–35. doi: 10.1038/s41556-026-02068-7 (PMC13661289; doi:10.1038/s41556-026-02068-7)
Supplement: Supplementary file 1 [file 41556_2026_2068_MOESM1_ESM.pdf]

nature cell biology

Supplementary information https://doi.org/10.1038/s41556-026-02068-7

Quantification of lipid sorting during clathrin-mediated endocytosis

In the format provided by the authors and unedited

Table of contents

SI Figures 1-12 NMR spectra of newly synthesized compounds 2
Description of Supplementary Data 1-4 14

NMR Spectra

Supplementary Figure 1 ^{1}H spectrum of PC(18:0|Y)

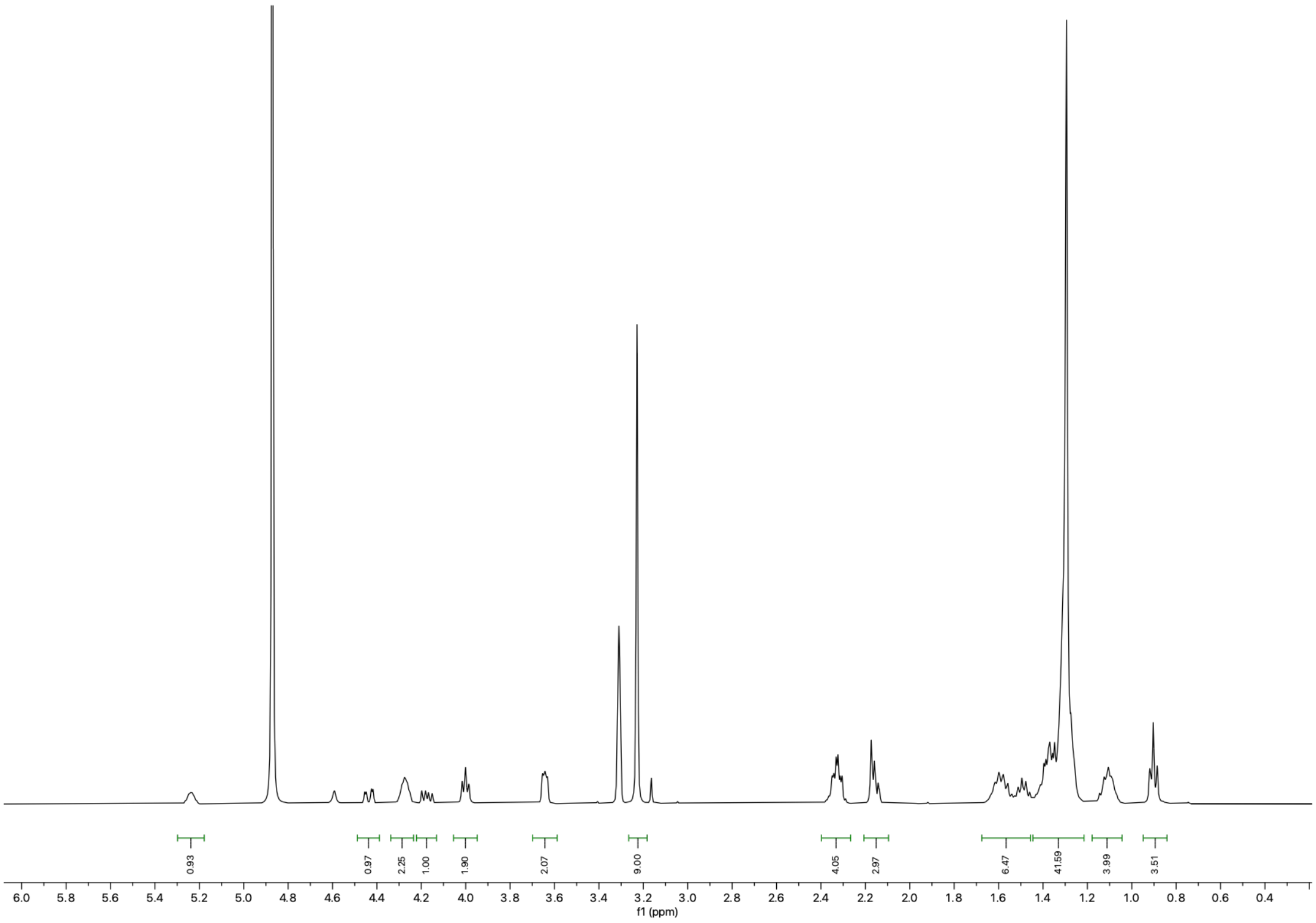

Supplementary Figure 2 ^{13}C spectrum PC(18:0|Y)

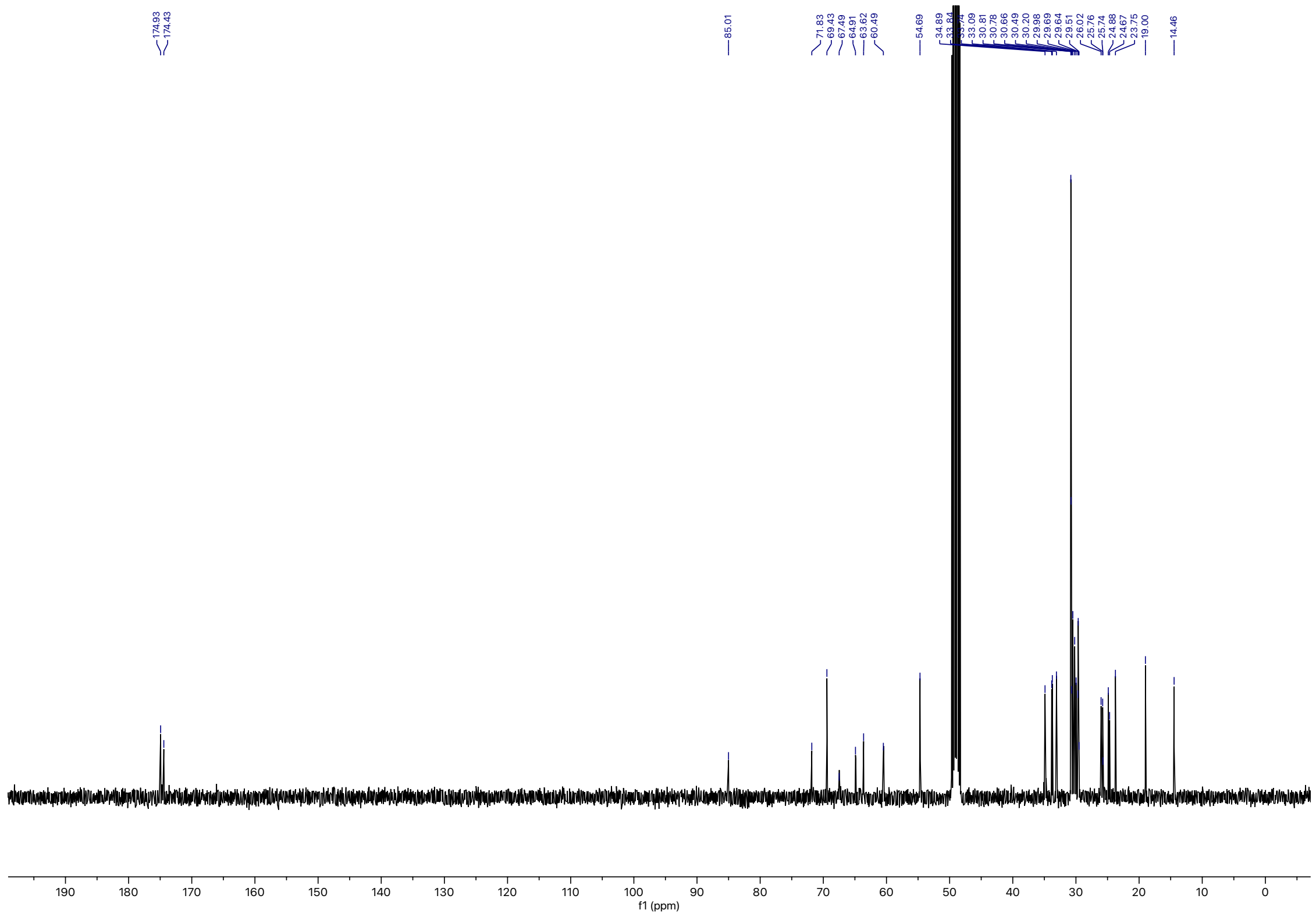

Supplementary Figure 3 ^{31}P spectrum of PC(18:0|Y)

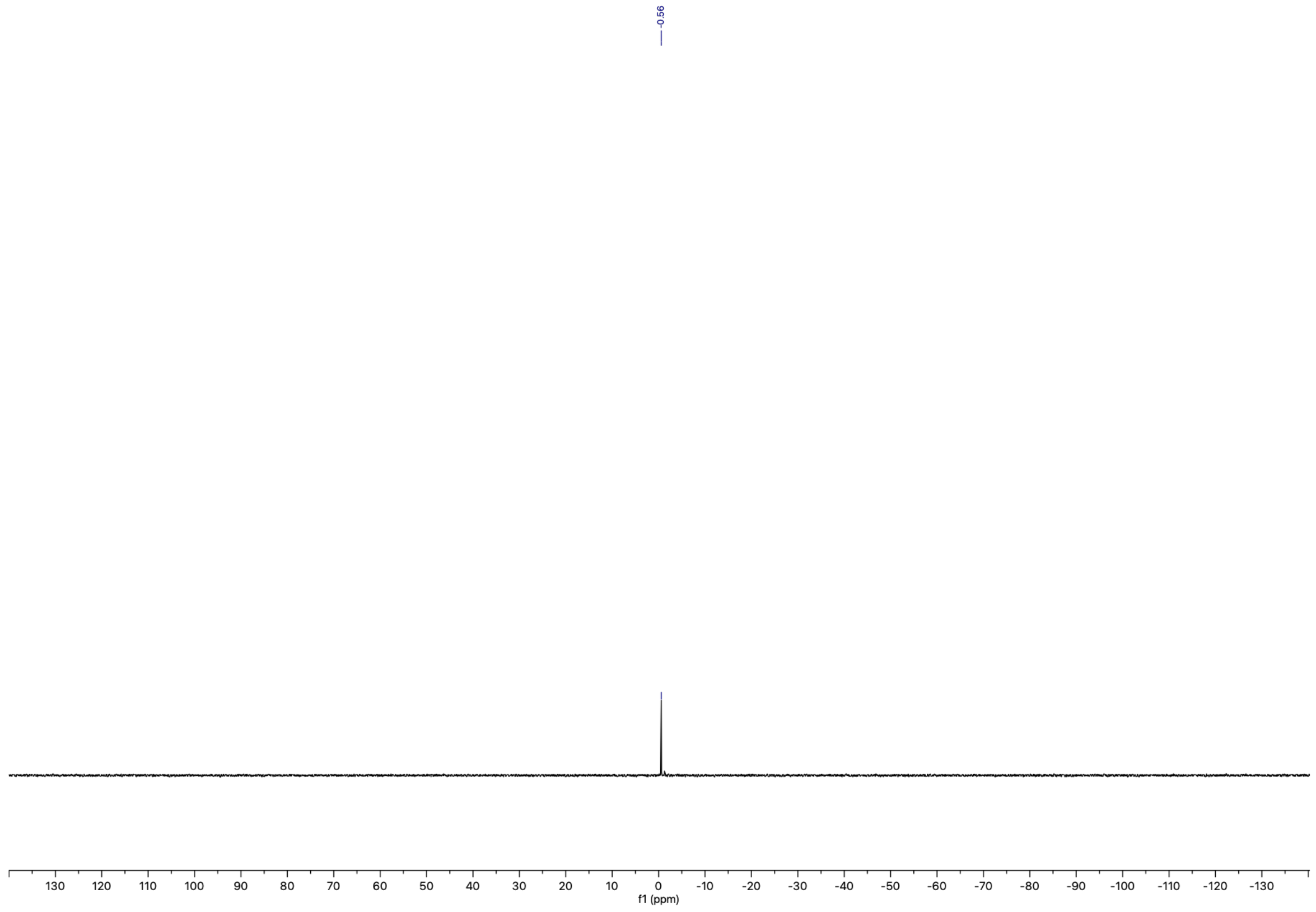

Supplementary Figure 4 ¹H spectrum of PC(20:0|Y)

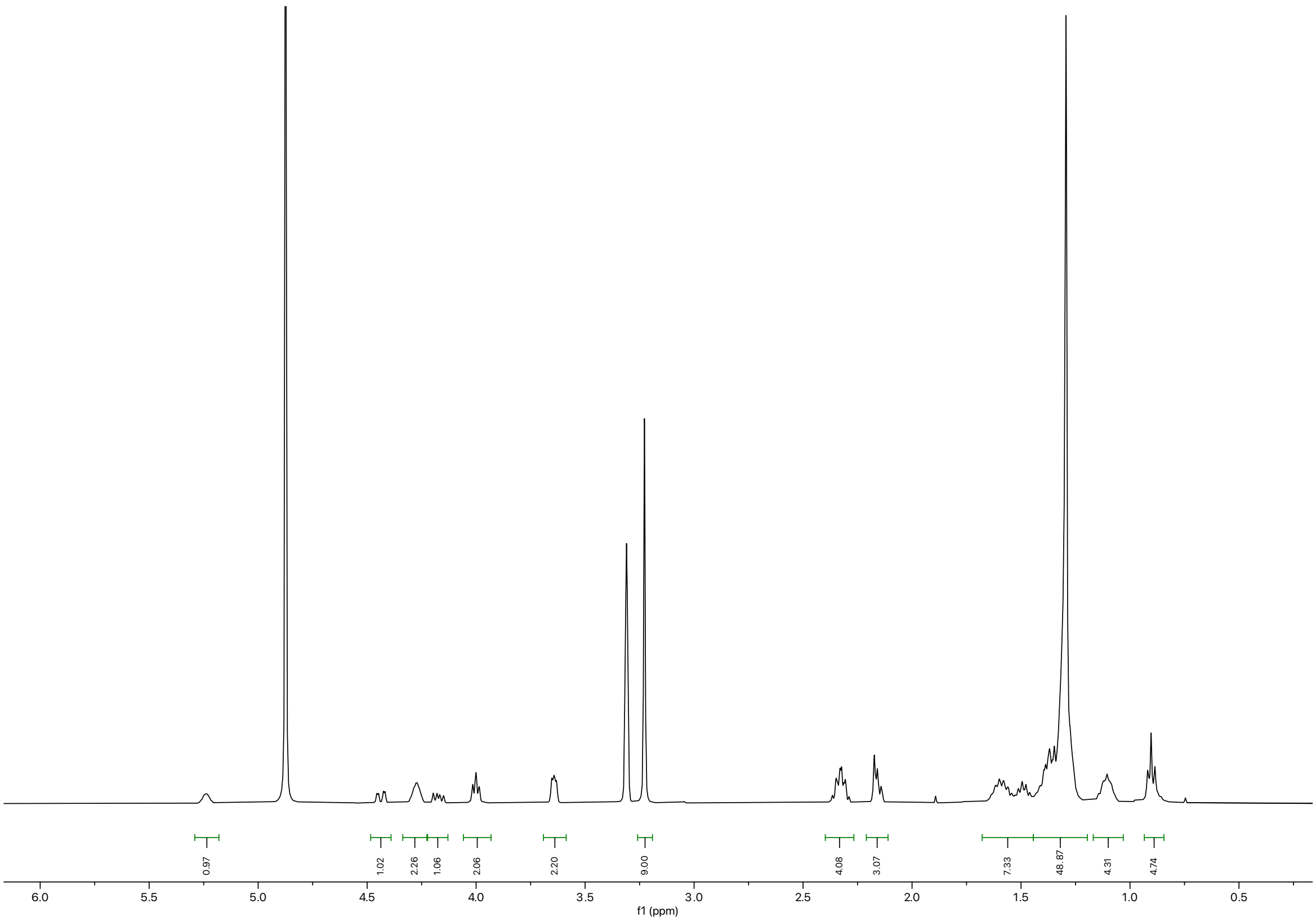

Supplementary Figure 5 ^{13}C spectrum of PC(20:0|Y)

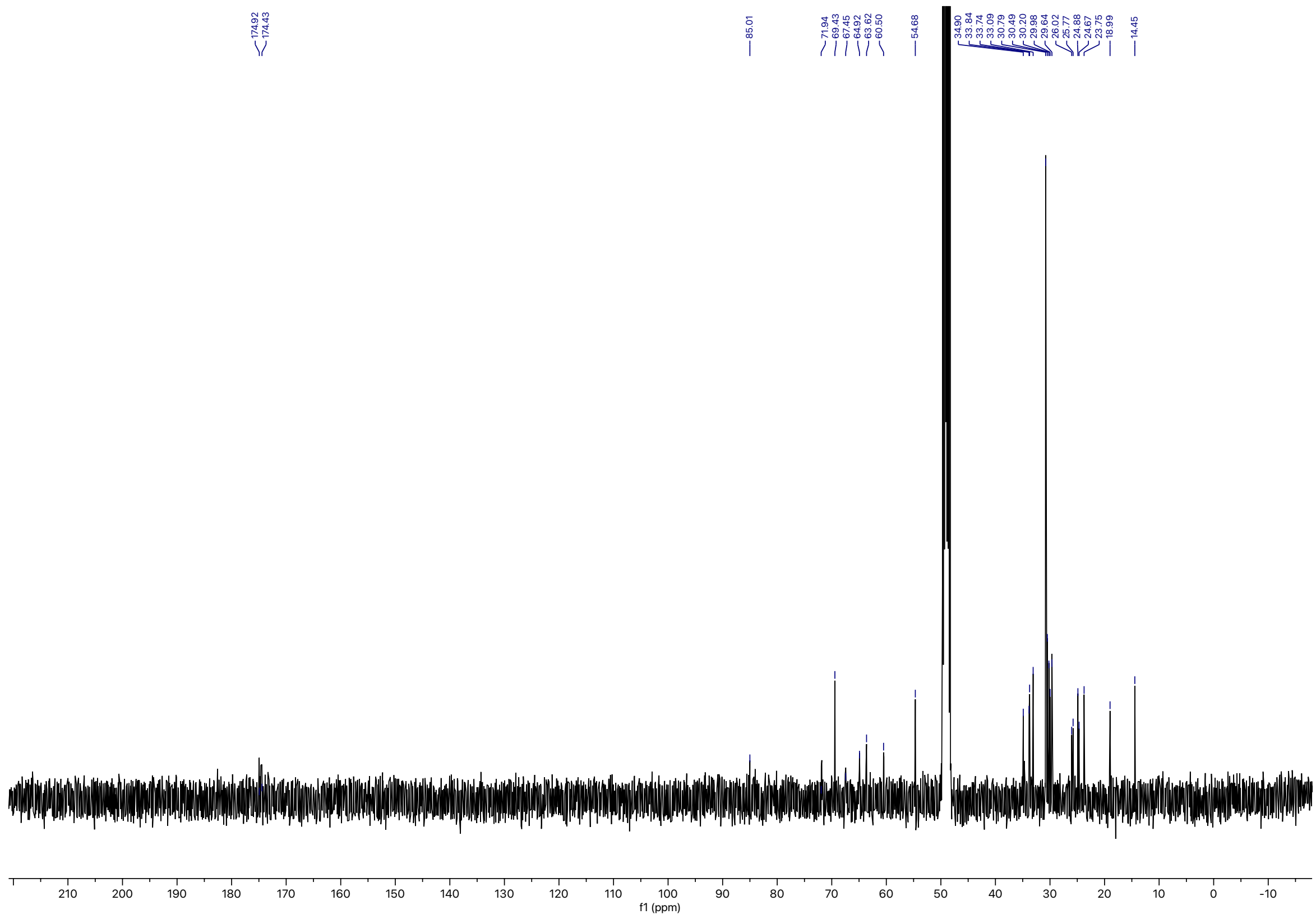

Supplementary Figure 6 ^{31}P spectrum of PC(20:0|Y)

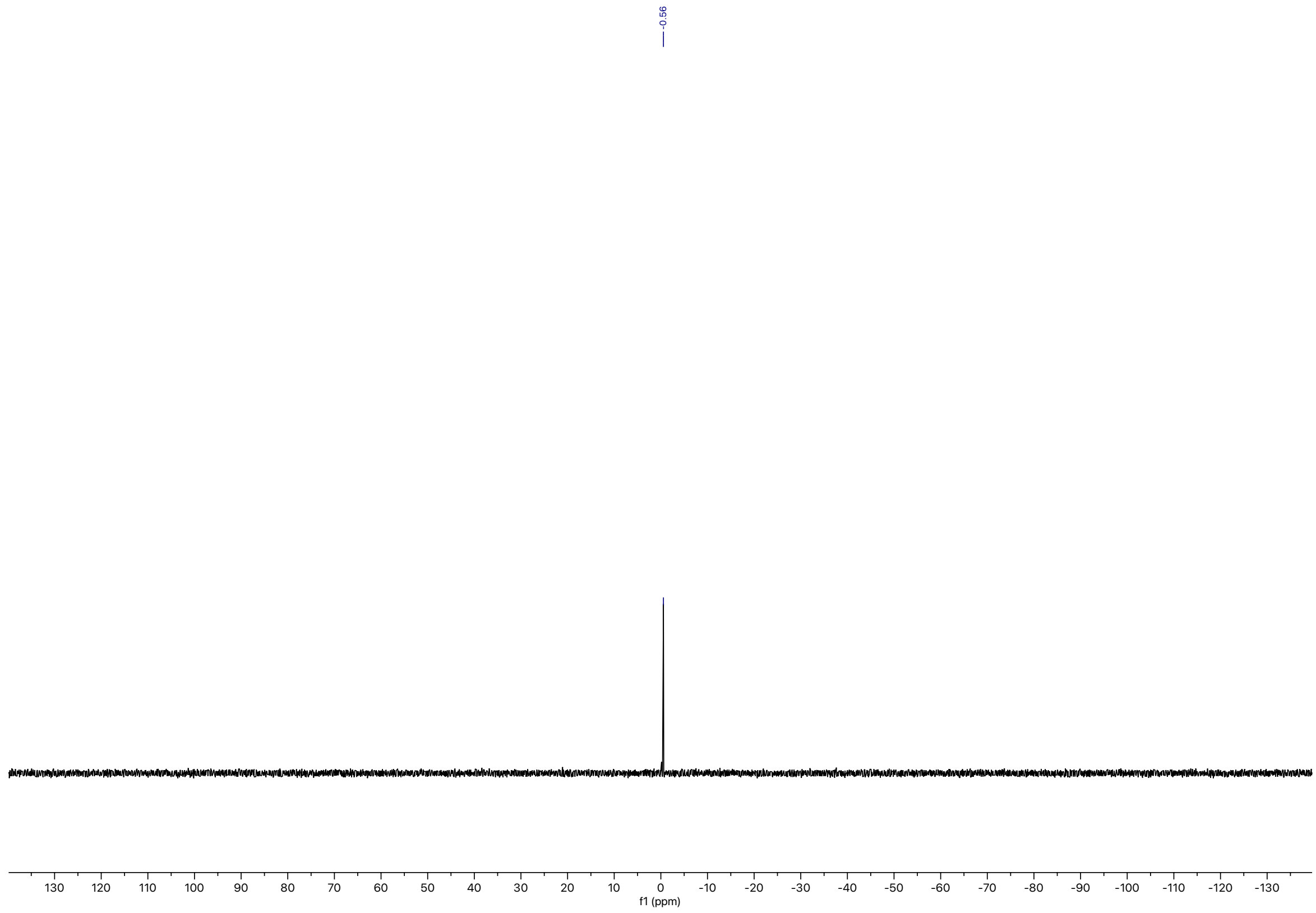

Supplementary Figure 7 ^1H spectrum of 13-Oxooctadec-17-ynoic acid

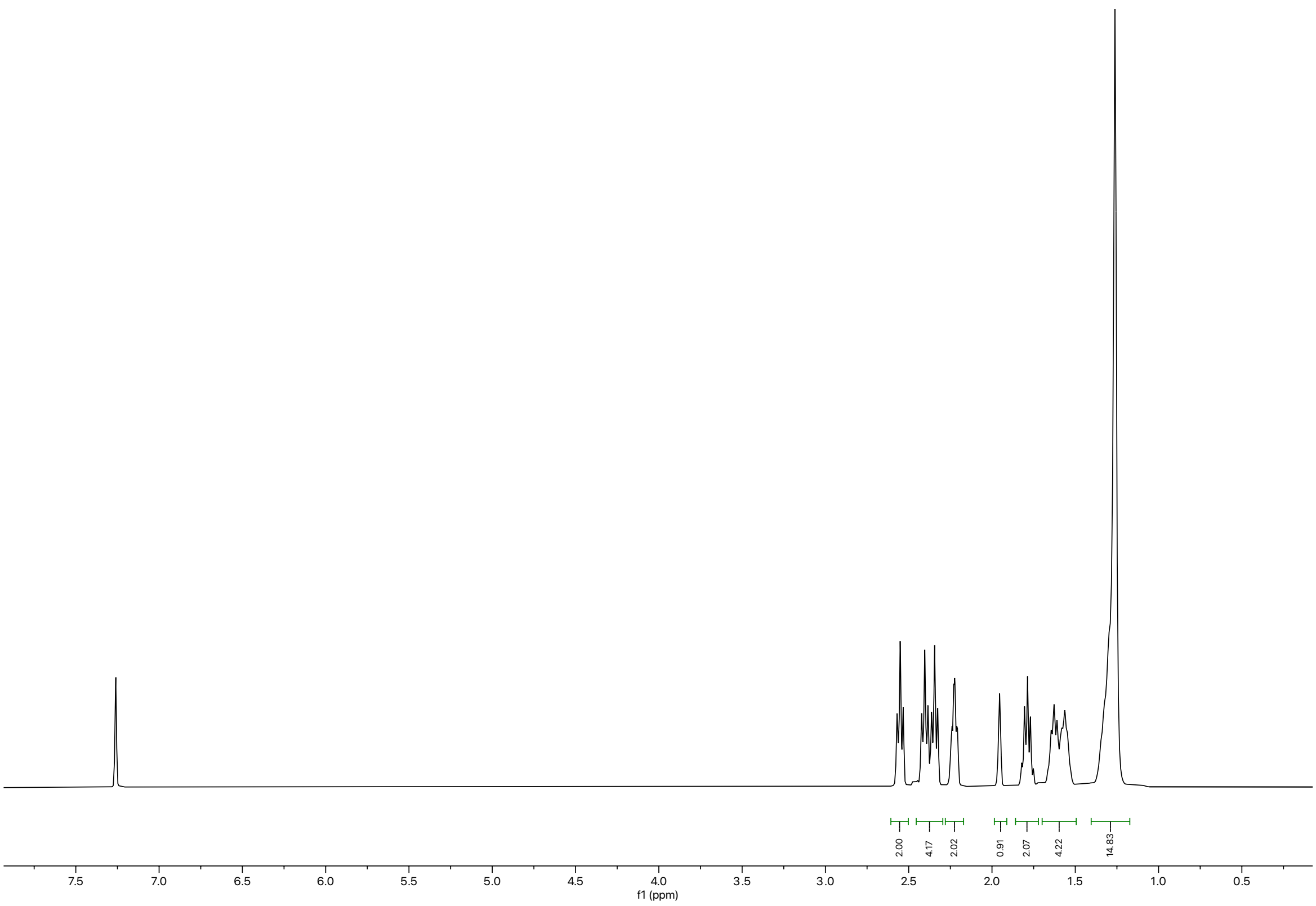

Supplementary Figure 8 ^{13}C spectrum of 13-Oxooctadec-17-ynoic acid

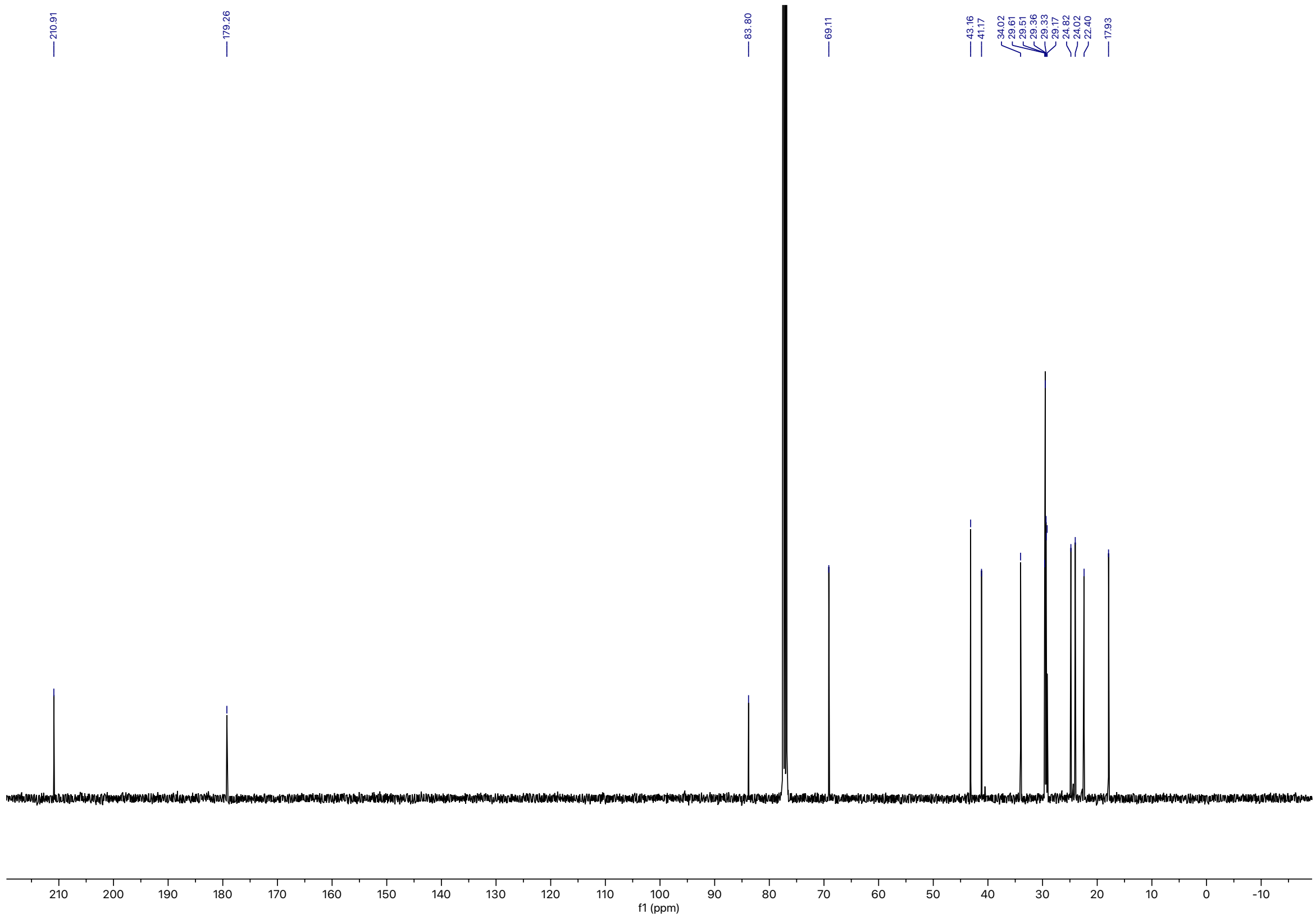

Supplementary Figure 9 ^{1}H spectrum of bifunctional fatty acid Y_{18}

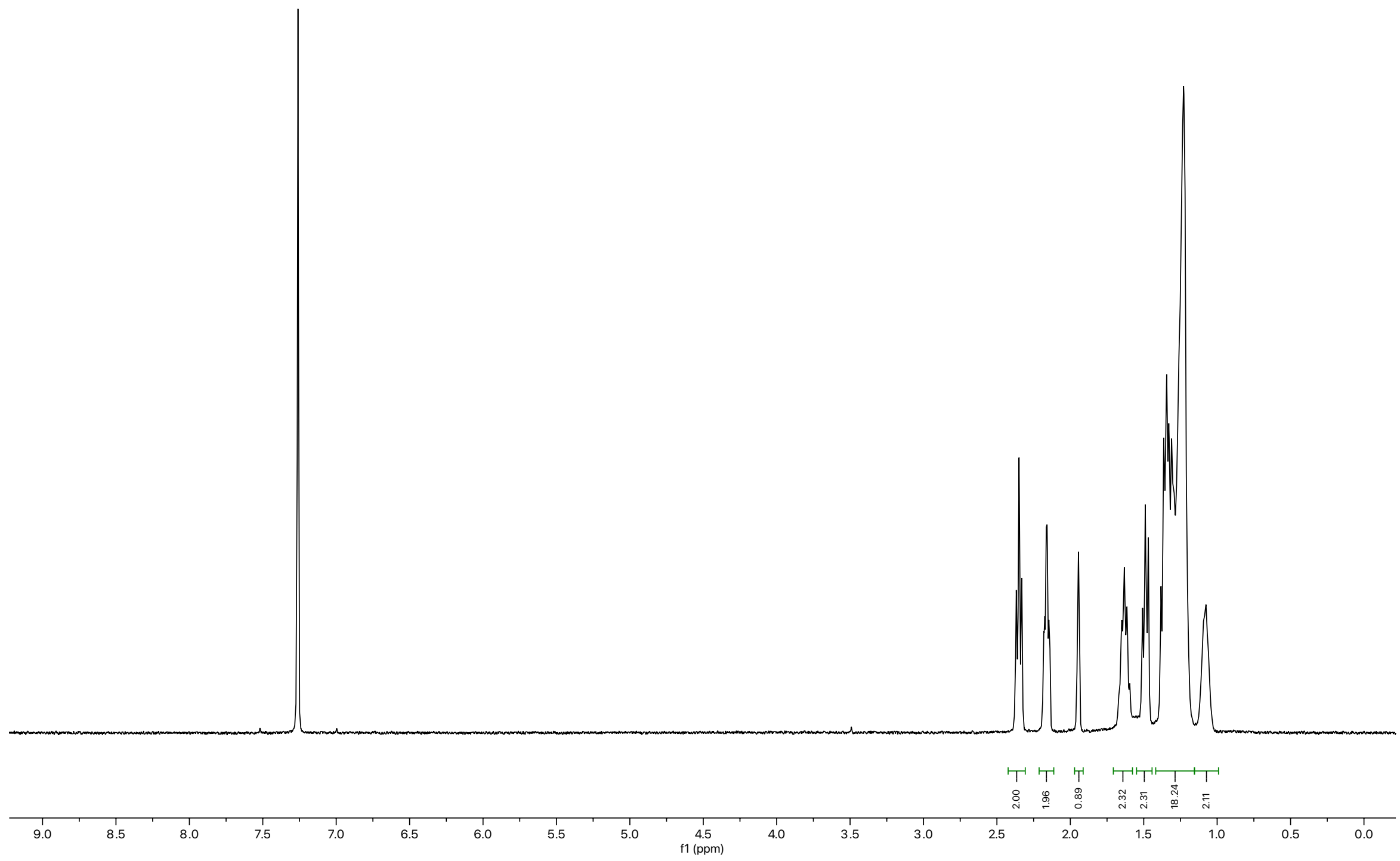

Supplementary Figure 10 ^{13}C spectrum of bifunctional fatty acid Y_{18}

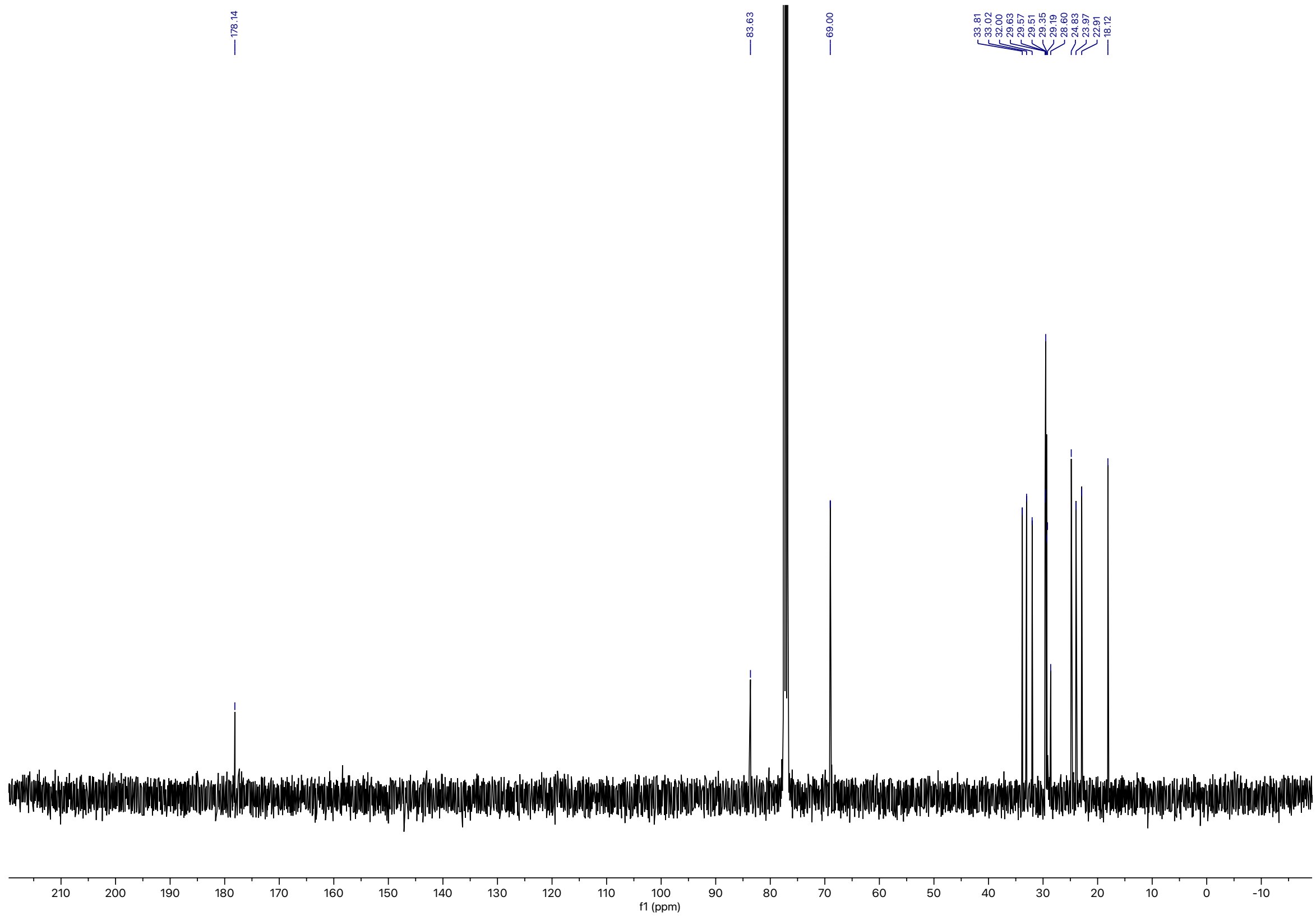

Supplementary Figure 11 ^{1}H spectrum of GlcCer(Y)

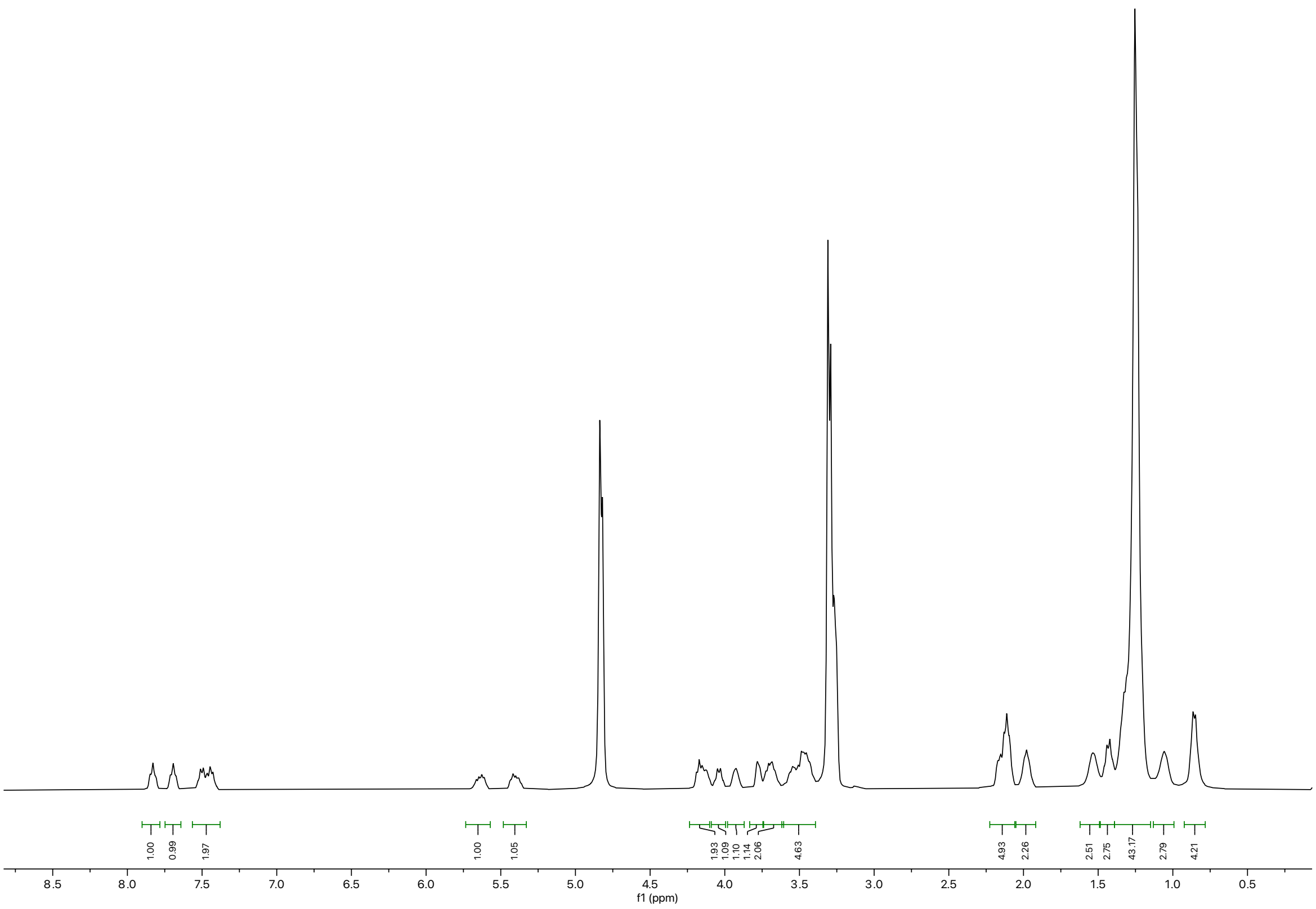

Supplementary Figure 12 ^{13}C spectrum of GlcCer(Y)

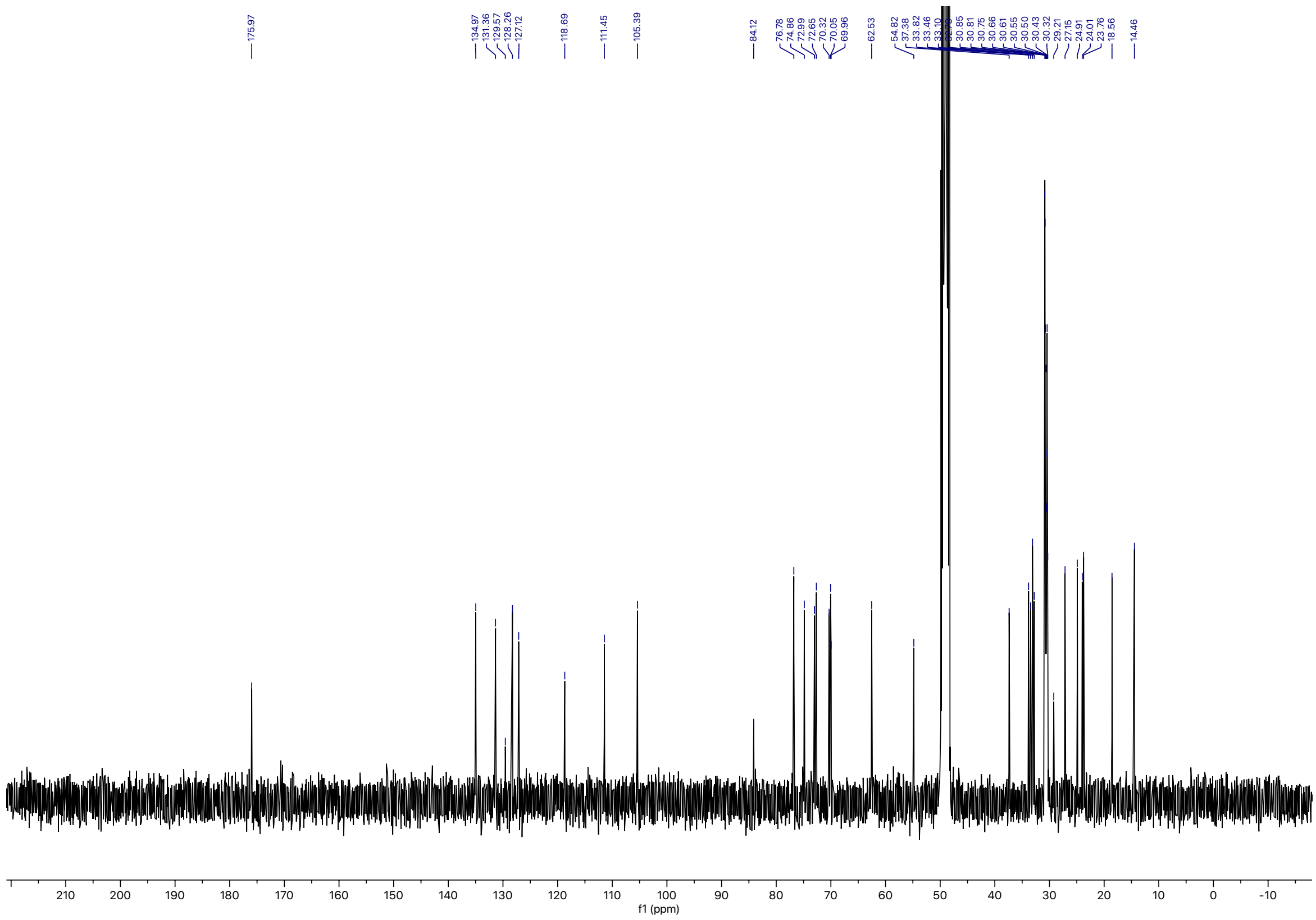

Supplementary Data descriptions

Supplementary Data 1: LED powers

The power output of a 308nm LED as previously reported [19], and a high-power 365nm LED with and without collimating lens were measured over 10s. Values are reported in SI Data 1.
A multi-LED strip suitable for synchronous photo-activation of 12 wells in a 96-well format was characterized for their power output per LED. The respective values are reported in SI Data 1

Supplementary Data 2: An estimation for geometry-based sorting of lipids into curved membranes

The Helfrich membrane bending energy [64] $E = \frac{1}{2}kA(C - Co)^2$ describes the energy cost to bend a surface area A of a membrane from its resting curvature C_0 is dependent on both the stiffness k of the membrane as well as the final curvature C. Both C_0, k, and the total bending energy depend on the lipid composition. In order to reduce the bending energy, lipids can either rearrange in order for C_0 to match C or to locally reduce k [65-69]. For determining the sorting of lipids with C_0 from a flat membrane to a bent membrane we calculate: $\Delta E = E_{flat} - E_{curved} = \frac{1}{2}ka[\left(C_{flat} - C_0\right)^2 - (C_{curved} - C_0)^2]$, with a boing the surface area of the lipid. We estimate the following values: k = ~20 k_BT; a = ~0.5 nm^2, C_{flat} = 0 nm-1; C_{curved} = 1/50 nm^{-1} or 1/25 nm^{-1}. Results are summarized SI Data 2

Supplementary Data 3: Values used for melting temperatures T_m of lipid species

The melting temperatures (T_m) of all experimentally assessed lipid species were determined from the literature, if possible. Values used in this study are summarized in SI Data 3.

Supplementary Data 4: An estimation for asymmetry-based sorting of lipids into curved membranes

We assume that lipid asymmetry is maintained in CCPs and that lipid probes have reached their steady state trans-bilayer distribution at the time point of photocrosslinking. We used the mol% of distinct lipid species per total phospholipids on the cytosolic leaflet and extracellular leaflet from Lorent et al. [36], which are shown SI Data 4. Next, we estimated the phospholipid content of the cytosolic and extracellular leaflet of a vesicle with an outer diameter (cytosolic, short: cyto) of 100 nm. The vesicle functions as a proxy for the highly curved late-stage CCP just before fission. We assume a bilayer thickness of 4 nm, and thus an inner (extracellular, short: extra) diameter of 92 nm. Thus, we calculate the surface areas A_{cyto} = 31416 nm^2 and A_{extra} = 26590 nm^2. We assume that 50% of the surface area (15708 nm^2) is covered by proteins of cylindrical shape. The surface area per phospholipid is estimated to 0.5 nm^2. Finally, we consider the asymmetric distribution of cholesterol, which drives an asymmetric distribution of phospholipids across both leaflets with an estimate of 42 mol% of all plasma membrane lipids on the cytosolic side and 22 mol% of all plasma membrane lipids on the extracellular side. These values were taken as estimates from Doktorova et al. [70]. Taken together, this renders a total of 13020 phospholipids to the cytosolic

side of the vesicle and 4840 to the extracellular side. We similarly estimate lipid numbers for a flat membrane with the surface area of A_{flat} = 31416 nm^2 to $\#_{cyto}$ = 13020 and $\#_{extra}$ = 6820.

To determine the numbers of lipid species per leaflet for a flat membrane and a curved membrane we take the product of the total estimated phospholipid number per leaflet with the previously reported mol% for the respective lipid species and leaflet as shown SI Data 4. Finally, the total estimated number is evaluated for the flat membrane and curved membrane (pit) and their ratio of the lipid number in the curved membrane over the number of lipids in the flat membrane (Ratio) is taken similar to the analysis to determine lipid pit enrichments for the STED data.
