# Supplementary material for: Quantification of lipid sorting during clathrin-mediated endocytosis
Source: Nat Cell Biol. 2026 Sep 29;28(10):2027–35. doi: 10.1038/s41556-026-02068-7 (PMC13661289; doi:10.1038/s41556-026-02068-7)
Supplement: Supplementary file 2 [file 41556_2026_2068_MOESM2_ESM.pdf]

nature portfolio

Corresponding author(s): André Nadler, Alf Honigmann

Last updated by author(s): May 20, 2026

Reporting Summary

Nature Portfolio wishes to improve the reproducibility of the work that we publish. This form provides structure for consistency and transparency in reporting. For further information on Nature Portfolio policies, see our Editorial Policies and the Editorial Policy Checklist.

Statistics

For all statistical analyses, confirm that the following items are present in the figure legend, table legend, main text, or Methods section.

n/a	Confirmed	
☐	☒	The exact sample size (*n*) for each experimental group/condition, given as a discrete number and unit of measurement
☐	☒	A statement on whether measurements were taken from distinct samples or whether the same sample was measured repeatedly
☐	☒	The statistical test(s) used AND whether they are one- or two-sided<br>*Only common tests should be described solely by name; describe more complex techniques in the Methods section.*
☐	☒	A description of all covariates tested
☐	☒	A description of any assumptions or corrections, such as tests of normality and adjustment for multiple comparisons
☐	☒	A full description of the statistical parameters including central tendency (e.g. means) or other basic estimates (e.g. regression coefficient) AND variation (e.g. standard deviation) or associated estimates of uncertainty (e.g. confidence intervals)
☐	☒	For null hypothesis testing, the test statistic (e.g. *F*, *t*, *r*) with confidence intervals, effect sizes, degrees of freedom and *P* value noted<br>*Give P values as exact values whenever suitable.*
☒	☐	For Bayesian analysis, information on the choice of priors and Markov chain Monte Carlo settings
☒	☐	For hierarchical and complex designs, identification of the appropriate level for tests and full reporting of outcomes
☒	☐	Estimates of effect sizes (e.g. Cohen's *d*, Pearson's *r*), indicating how they were calculated

Our web collection on statistics for biologists contains articles on many of the points above.

Software and code

Policy information about availability of computer code

Data collection	Olympus cellSens Dimension (Version 4.1), Imspector software (v.16.2.8415)
Data analysis	python 3 (Version 3.8 and 3.10)<br>Ilastik (Version 1.4.0-OSX)<br>Prism 10 (Version 10.4.0 (527))<br>ImageJ/Fiji (Version 2.14.0/ 1.54f)

For manuscripts utilizing custom algorithms or software that are central to the research but not yet described in published literature, software must be made available to editors and reviewers. We strongly encourage code deposition in a community repository (e.g. GitHub). See the Nature Portfolio guidelines for submitting code & software for further information.

Data

Policy information about availability of data

All manuscripts must include a data availability statement. This statement should provide the following information, where applicable:

- Accession codes, unique identifiers, or web links for publicly available datasets
- A description of any restrictions on data availability
- For clinical datasets or third party data, please ensure that the statement adheres to our policy

All software codes used for analysis and data have been deposited on the Max Planck Institute of Molecular Cell Biology and Genetics repository and are available for the reader at https://doi.org/10.17617/3.GLIVDU and upon request from the corresponding authors.

Research involving human participants, their data, or biological material

Policy information about studies with human participants or human data. See also policy information about sex, gender (identity/presentation), and sexual orientation and race, ethnicity and racism.

Reporting on sex and gender	n/a
Reporting on race, ethnicity, or other socially relevant groupings	n/a
Population characteristics	n/a
Recruitment	n/a
Ethics oversight	n/a

Note that full information on the approval of the study protocol must also be provided in the manuscript.

Field-specific reporting

Please select the one below that is the best fit for your research. If you are not sure, read the appropriate sections before making your selection.

☒ Life sciences ☐ Behavioural & social sciences ☐ Ecological, evolutionary & environmental sciences

For a reference copy of the document with all sections, see nature.com/documents/nr-reporting-summary-flat.pdf

Life sciences study design

All studies must disclose on these points even when the disclosure is negative.

Sample size	No sample size calculation was performed. The low variability between the different labeling conditions shows that a small number of independent repeats is sufficient. For the determination of the lipid densities in clathrin coated pits 3 biological replicates were performed per condition, comprising at least 1500 pits in total.
Data exclusions	STED images were excluded if the acquisition process failed by either being out of focus or poor laser alignment. Images acquired by spinning disc microscopy were excluded if the acquisition process failed by either being out of focus or if unwanted cellular debris prevented image analysis.
Replication	All attempts at replication were successful between biological and technical replicas. Sample preparation and data acquisition were reproducibly performed by different people.
Randomization	Images were taken at random positions in the wells of a 96 well-plate.
Blinding	n/a

Reporting for specific materials, systems and methods

We require information from authors about some types of materials, experimental systems and methods used in many studies. Here, indicate whether each material, system or method listed is relevant to your study. If you are not sure if a list item applies to your research, read the appropriate section before selecting a response.

Materials & experimental systems

n/a	Involved in the study
☐	☒ Antibodies
☐	☒ Eukaryotic cell lines
☒	☐ Palaeontology and archaeology
☒	☐ Animals and other organisms
☒	☐ Clinical data
☒	☐ Dual use research of concern
☒	☐ Plants

Methods

n/a	Involved in the study
☒	☐ ChIP-seq
☒	☐ Flow cytometry
☒	☐ MRI-based neuroimaging

Antibodies

Antibodies used

AP2 (mouse), Invitrogen MA1-064, Clone: AP6, Lot:WB315160
Clathrin light chain (rabbit), proteintech 10852-1-AP, Lot: 00097561
EEA1 (mouse), BD Bioscience 610457, Clone: 14, Lot: 1117266
LDL receptor (mouse), Invitrogen, MA5-38556, Clone: 1B10H10, Lot: 80346632
Transferrin receptor (mouse), Invitrogen, 13-6800, Clone: H68.4, Lot: ZI396641
GFP booster, Chromotek, gb2AF488-50, Lot: LM00000353

Validation

All primary antibodies were used directly from the manufacturer. All antibodies were validated by the manufacturers to be suitable for immunofluorescence (IF) and reactive with Human samples.

AP2, MA1-064 -> Applications: WB, IHC, ICC/IF, FC, IP. Species Reactivity: Human, Mouse, Hamster, Bovine, Rat, Primate
Clathrin light chain, 10852-1-AP -> Applications: WB, IP, IHC, IF/ICC, ELISA. Species Reactivity: Mouse, Human, Rat
EEA1, 610457 -> Applications: WB, IF, IP, IHC(P). Species Reactivity: Human
LDL receptor, MA5-38556 -> Applications: WB, IHC (P), ICC/IF, Flow, ELISA. Species Reactivity: Human
Transferrin receptor, 13-6800 -> Application: WB, IHC (P), ICC/IF, Flow, ELISA, IP. Species Reactivity: Human, Hamster, Chicken, Mouse, Rat
GFP booster, gb2AF488 -> Applications: IF, IHC, ICC. Species Reactivity: GFP

Eukaryotic cell lines

Policy information about cell lines and Sex and Gender in Research

Cell line source(s)

SK-MEL2 dynamin-2-GFP and SK-MEL2 wildtype cells were provided by David G. Drubin as described by Doyon et al., 2011. HEK293 were acquired from DSMZ. Ref: ACC305

Authentication

SK-MEL2 dynamin-2-GFP and SK-MEL2 wildtype cells were authenticated by David G. Drubin as described by Doyon et al., 2011. HEK293 were authenticated by DSMZ.

Mycoplasma contamination

All cell lines are routinely tested for Mycoplasma contamination

Commonly misidentified lines
(See ICLAC register)

n/a

Plants

Seed stocks

n/a

Novel plant genotypes

n/a

Authentication

n/a
