# Supplementary material for: Quantification of lipid sorting during clathrin-mediated endocytosis
Source: Nat Cell Biol. 2026 Sep 29;28(10):2027–35. doi: 10.1038/s41556-026-02068-7 (PMC13661289; doi:10.1038/s41556-026-02068-7)
Supplement: Supplementary file 3 [file 41556_2026_2068_MOESM3_ESM.pdf]

nature portfolio

Peer Review File

Quantification of lipid sorting during clathrin-mediated endocytosis

Corresponding Author: Dr André Nadler

Version 0:

Decision Letter:

*Please delete the link to your author homepage if you wish to forward this email to co-authors.

Dear Dr Nadler,

Your manuscript, "Quantification of lipid sorting during clathrin-mediated endocytosis", has now been seen by 3 referees, who are experts in lipid transport and membranes(referee 1); Super-resolution microscopy and methods development (referee 2); and CME and imaging(referee 3). As you will see from their comments (attached below) they find this work of potential interest, but have raised substantial concerns, which in our view would need to be addressed in full with considerable revisions before we can consider publication in Nature Cell Biology.

Nature Cell Biology editors discuss the referee reports in detail within the editorial team, including the chief editor, to identify key referee points that should be addressed with priority, and requests that are overruled as being beyond the scope of the current study. To guide the scope of the revisions, I have listed these points below. We are committed to providing a fair and constructive peer-review process, so please feel free to contact me if you would like to discuss any of the referee comments further.

Please note that given this manuscript relies on methodology in a separate manuscript (NCB-TR57011), we would require the Technical Report to be published first or simultaneously. In addition, significant biological insight must be provided in the revision of this paper to distinguish it from the technical report.

In particular, it would be essential to:

Further develop the conceptual advance of this manuscript by further experimental evidence supporting the model that lipid asymmetry drives lipid sorting. We would also expect all comments regarding the lipid composition of different membranes and assessing the outcome of using flippases to alter lipid composition to be addressed.

All other referee concerns pertaining to strengthening existing data, providing controls, methodological details, clarifications and textual changes, should also be addressed.

Finally please pay close attention to our guidelines on statistical and methodological reporting (listed below) as failure to do so may delay the reconsideration of the revised manuscript. In particular please provide:

- a Supplementary Figure including unprocessed images of all gels/blots in the form of a multi-page pdf file. Please ensure that blots/gels are labeled and the sections presented in the figures are clearly indicated.

- a Supplementary Table including all numerical source data in Excel format, with data for different figures provided as different sheets within a single Excel file. The file should include source data giving rise to graphical representations and statistical descriptions in the paper and for all instances where the figures present representative experiments of multiple independent repeats, the source data of all repeats should be provided.

We would be happy to consider a revised manuscript that would satisfactorily address these points, unless a similar paper is published elsewhere, or is accepted for publication in Nature Cell Biology in the meantime.

When revising the manuscript please:

- ensure that it conforms to our format instructions and publication policies (see below and https://www.nature.com/nature/for-authors).

- provide a point-by-point rebuttal to the full referee reports verbatim, as provided at the end of this letter.

- provide the completed Reporting Summary (found here https://www.nature.com/documents/nr-reporting-summary.pdf). This is essential for reconsideration of the manuscript will be available to editors and referees in the event of peer review. For more information see http://www.nature.com/authors/policies/availability.html or contact me.

When submitting the revised version of your manuscript, please pay close attention to our href="https://www.nature.com/nature-portfolio/editorial-policies/image-integrity">Digital Image Integrity Guidelines. and to the following points below:

-- that unprocessed scans are clearly labelled and match the gels and western blots presented in figures.
-- that control panels for gels and western blots are appropriately described as loading on sample processing controls
-- all images in the paper are checked for duplication of panels and for splicing of gel lanes.

Finally, please ensure that you retain unprocessed data and metadata files after publication, ideally archiving data in perpetuity, as these may be requested during the peer review and production process or after publication if any issues arise.

Nature Cell Biology is committed to improving transparency in authorship. As part of our efforts in this direction, we are now requesting that all authors identified as 'corresponding author' on published papers create and link their Open Researcher and Contributor Identifier (ORCID) with their account on the Manuscript Tracking System (MTS), prior to acceptance. ORCID helps the scientific community achieve unambiguous attribution of all scholarly contributions. You can create and link your ORCID from the home page of the MTS by clicking on 'Modify my Springer Nature account'. For more information please visit please visit www.springernature.com/orcid.

This journal strongly supports public availability of data. Please place the data used in your paper into a public data repository, or alternatively, present the data as Supplementary Information. If data can only be shared on request, please explain why in your Data Availability Statement, and also in the correspondence with your editor. Please note that for some data types, deposition in a public repository is mandatory - more information on our data deposition policies and available repositories appears below.

Please submit the revised manuscript files and the point-by-point rebuttal to the referee comments using this link:

Link Redacted

*This url links to your confidential home page and associated information about manuscripts you may have submitted or be reviewing for us. If you wish to forward this email to co-authors, please delete the link to your homepage.

We would like to receive a revised submission within six months.

We hope that you will find our referees' comments, and editorial guidance helpful. Please do not hesitate to contact me if there is anything you would like to discuss.

Best wishes,

Angela Parrish

Angela R Parrish, PhD
Locum Senior Editor
Nature Cell Biology

Reviewers' Comments:

Reviewer #1 (Remarks to the Author):

In this manuscript, Lennartz et al present a compelling study of lipid partitioning into clathrin-coated pits. It addresses a big, open question that many have approached but none have carefully investigated – is there lipid selectivity in the clathrin-mediated endocytosis? This study answers the question - quite definitively - with advanced imaging of carefully designed chemical probes combined with rigorous analysis and modeling. The latter is essential and non-trivial: clathrin pits are 3D objects usually imaged in 2D projections that dramatically convolute shape and lateral organization (cartoon in Fig 2D is nice: makes an important, quantitatively accurate point). The approach here is smart and rigorous, relying on EM images of pits to model their structures and the resulting effect on lipid imaging.

The short answer to the big question is that there is almost no lateral lipid selectivity, with the observed trends fitting well to lipid transbilayer distributions. That is, there is less outer leaflet area in a CCP, so lipids of the outer leaflet should be generically slightly excluded. This may sound like a 'negative' result, but it isnt. 'No selectivity' is still an interesting – and quite surprising – effect. It is to the authors credit that they emphasize the clear major conclusion rather than trying to hype up small differences.

Overall, I find the work rigorous, interesting, and beautifully presented. My only significant suggestions are for the Discussion: I point out a couple of specifics, though I feel that the authors could expand on other possibilities (eg selectivity of non-clathrin-mediated vesicular transport).

The manuscript is very nicely written: clear, concise, and direct. But the Discussion is too short, omitting some important points:
a. the strong correlation to lipid Tm is probably also explainable by asymmetry… but the first order effect is exactly what the authors were testing (ie phase enrichment), just backwards from expectation. I think this should at least be discussed.
b. Relatedly, it is probably an important caveat to mention that the while the analogs used here are indeed "near-native", the diazerene moiety seems likely to perturb acyl chain packing, in the same direction that a double-bond would. As the authors surely recognize, its an important caveat for their rafty interpretations (or conspicuous lack thereof).
c. My only major gripe is with the correlations to expected lipid physical properties. Again, as with the rest of the paper, its careful, rigorous analysis. But the presentation gives the impression that the lipid shapes, melting temps, etc are actually known, which they are not for these analogs. It is probably the case that the trends to the native lipids are generally maintained, since the tags are always similar. But Fig 4C really implies that these are real values, not inferred or proxies. I don't have a recommendation for how to be more circumspect with the data presentation, but minimally the caveat should be discussed

Minor wording issues:
1. Last sentence of abstract is odd. Are the authors intending to highlight their technology as a major advance? That's not obvious from the wording and its not clear why this sentence occupies this position of importance.
2. The orange images representing lipid signal are a bit unclear to me: is it possible to estimate how many molecules a given intensity level represents? Maybe this is too difficult or irrelevant, but its not clear whether the bright spots represent accumulations or individual localizations, or similarly, whether the orange haze represents noise or fewer (but real) fluorescent molecules.
3. "if their neck diameter measured less than 40 nm" – does this refer to the modeled shapes or actual measurements from EM?
4. "…geometries and resulting apparent partitioning values are not normally distributed" I don't understand the explanation here ("directly reflects the original EM dataset"), nor why a normal distribution would necessarily be the expectation.
5. "A closer inspection of the modeled pit structures" what is the point of this paragraph? Does this have any influence on the interpretation of experiments?
6. "temporally highly resolved uptake kinetics of all lipids into early endosomes" these data look nice and seem quite rigorous, and also nicely align with the partitioning measurements. Why are they relegated to being the only figures in Supplement?
7. Its pretty confusing that there are both "Extended" and "Supplementary" figures. See rec above to move Supp Figs into main manuscript.
8. The red lines in Fig 4C are weird. I totally don't understand the one for lipid shape… and this behavior is also inherently conflated with asymmetry (inverted cone fits well if on outer leaflet, not on inner). More minor, why is the red line shifted in rightmost graph? If its just a cartoon, might as well put it right on top of your fit!

Reviewer #2 (Remarks to the Author):

The manuscript titled " Quantification of lipid sorting during clathrin-mediated endocytosis" investigates the role of clathrin-mediated endocytosis (CME) in lipid sorting and quantifies the distribution of 10 different lipids in clathrin-coated pits (CCPs) using super-resolution STED microscopy and mathematical modeling. The findings reveal that the differential distribution of lipids in CCPs is primarily driven by lipid asymmetry in the cell membrane. By integrating super-resolution STED microscopy with mathematical modeling, the study provides a novel method for quantifying lipid distribution in cellular membrane compartments. This technological advancement offers an important tool for future cell biology research, with potential applications.
Major issues
1. The lipid probes used in the study were chemically modified (e.g., by introducing diazirine and alkyne groups). Might these alterations substantially impact their polarity, solubility, or protein-binding affinity? Could such changes influence their distribution and behavior within the cell membrane? Additionally, could the use of these modified probes disrupt normal cellular processes, such as membrane dynamics or protein function? Are there experimental approaches available to exclude these possible effects?
2. Can these probes effectively detect the distribution of lipids on endosomes?
3. The study's mathematical model accounts for the intricate geometry and dynamic behavior of CCPs, suggesting that lipid asymmetry in the cell membrane is the key driver of lipid sorting in CCPs. Is it possible to further confirm this hypothesis with additional experimental evidence?
4. Are there variations in lipid distribution within the cell membranes of different cell types, such as phagocytic versus non-phagocytic cells? If such differences exist, could they impact the phagocytic capabilities of these cells?

Reviewer #3 (Remarks to the Author):

If and how the lipids are sorted by clarthrin-mediated endocytosis (CME) is an important biological question. In this paper, Lennartz et al. used super-resolution STED imaging of bifunctional lipid probes, in parallel with mathematical modeling, to compare the partitioning of 10 structurally diverse lipid species at clathrin-coated pits. Their quantitative analysis observes moderate but significant differences of pit partitioning among the 10 lipid species. By correlation analysis with known biophysical properties of lipids, the authors concluded that the moderate differences in pit partitioning are due to leaflet asymmetry of various lipids. Before this study, it was not possible to directly compare lipid partitioning, and pairing the experiments with geometric modeling allowed the authors to extract a weak biological signal. This work provided important direct quantitative evidence for the differential enrichment of several lipid groups at clarthrin-coated pits. Overall, the data presentation is clear and information-rich. However, two important lipid groups on plasma membrane, phosphatidylserine and phosphatidylinositol, which are implicated in playing key roles during CME, are not examined and are not considered for the mechanism they proposed. The conclusion that inner leaflet residence predicts lipid partitioning was drawn from an oversimplified analysis. In particular, this analysis assumes that all lipid species are primarily partitioned by the same mutually exclusive partitioning mechanism. The differential partitioning data is supported and interesting, but the underlying mechanism is not sufficiently explored.

Major comments:
• The reference papers validating the method in the first half of the work are posted on bioRxiv, but a peer-reviewed version is not publicly available.
• The abstract states there is “time resolved data” but only late-stage CME sites are analyzed. Figures depicting time evolution of CME, or the modeling of the signal for early-stage CME sites set the reader up to expect time-resolved data.
• Figure 4 legend title: “Sufficient to explain” is misleading, while the authors only demonstrate “consistent with a model of” or a correlation.
• The authors should apply geometric modeling to predict expected lipid enrichment for inner- and outer-leaflet resident lipids. Is the relative partitioning of the same order of magnitude as the differences in surface area the authors claim to explain the partitioning?
• The conclusion of the second half of the paper, that inner/outer leaflet residence drives lipid partitioning, would be stronger if the authors introduced a biological perturbation that flips the lipid residence, and tests if it changes the partitioning.
• An important hypothesis that the authors discuss in the Discussion but do not test is that lipid-binding proteins may stabilize some lipid species at CME sites.

Minor comments:
• The Mund&Tchanz fixation protocol is cited by names, but does not match what is reported in the materials and methods.
• In general, there are several acronyms that are not defined (Ld, Lo) or are defined after use in the text (lipid names).
• Figure 1:
o SK-MEL-2 are not typically round after being plated in the reported conditions. Cells shown in the Supplement exhibit the expected morphology. Choose a more representative image for this figure.
o Depicting how the exogenously added tagged lipids enter the inner leaflet in the cartoon would make the remainder of the paper easier to read.
o Figure 1 B and Figure 1C subpanels mislead readers to expect time-resolved data.
• Figure 2:
• Remove modeling of expected intensities for early-stage CME sites.
• Fig2A: The outline of the CME site in the last panel changes color, and is confusing to the reader.
• Figure 3:
o Consider moving Fig 3A to Fig1, so that the readers (1) understand the lipid naming scheme used in the paper text and (2) establishes the lipids used in the studies to establish expectations for the readers.

GUIDELINES FOR MANUSCRIPT SUBMISSION TO NATURE CELL BIOLOGY

READABILITY OF MANUSCRIPTS – Nature Cell Biology is read by cell biologists from diverse backgrounds, many of whom are not native English speakers. Authors should aim to communicate their findings clearly, explaining technical jargon that might be unfamiliar to non-specialists, and avoiding non-standard abbreviations. Titles and abstracts should concisely communicate the main findings of the study, and the background, rationale, results and conclusions should be clearly explained in the manuscript in a manner accessible to a broad cell biology audience. Nature Cell Biology uses British spelling.

MANUSCRIPT FORMAT – please follow the guidelines listed in our Guide to Authors regarding manuscript formats at Nature Cell Biology.

TITLE – should be no more than 100 characters including spaces, without punctuation and avoiding technical terms, abbreviations, and active verbs..

AUTHOR NAMES – should be given in full.

AUTHOR AFFILIATIONS – should be denoted with numerical superscripts (not symbols) preceding the names. Full addresses should be included, with US states in full and providing zip/post codes. The corresponding author is denoted by: "Correspondence should be addressed to [initials]."

ABSTRACT AND MAIN TEXT – please follow the guidelines that are specific to the format of your manuscript, as listed in our Guide to Authors (http://www.nature.com/ncb/pdf/ncb_gta.pdf) Briefly, Nature Cell Biology Articles, Resources and Technical Reports have 3500 words, including a 150 word abstract, and the main text is subdivided in Introduction, Results, and Discussion sections. Nature Cell Biology Letters have up to 2500 words, including a 180 word introductory paragraph (abstract), and the text is not subdivided in sections.

ACKNOWLEDGEMENTS – should be kept brief. Professional titles and affiliations are unnecessary. Grant numbers can be listed.

AUTHOR CONTRIBUTIONS – must be included after the Acknowledgements, detailing the contributions of each author to the paper (e.g. experimental work, project planning, data analysis etc.). Each author should be listed by his/her initials.

FINANCIAL AND NON-FINANCIAL COMPETING INTERESTS – the authors must include one of three declarations: (1) that they have no financial and non-financial competing interests; (2) that they have financial and non-financial competing interests; or (3) that they decline to respond, after the Author Contributions section. This statement will be published with the article, and in cases where financial and non-financial competing interests are declared, these will be itemized in a web supplement to the article. For further details please see https://www.nature.com/licenceforms/nrg/competing-interests.pdf.

REFERENCES – are limited to a total of 70 for Articles, Resources, Technical Reports; and 40 for Letters. This includes references in the main text and Methods combined. References must be numbered sequentially as they appear in the main text, tables and figure legends and Methods and must follow the precise style of Nature Cell Biology references. References only cited in the Methods should be numbered consecutively following the last reference cited in the main text. References only associated with Supplementary Information (e.g. in supplementary legends) do not count toward the total reference limit and do not need to be cited in numerical continuity with references in the main text. Only published papers can be cited, and each publication cited should be included in the numbered reference list, which should include the manuscript titles. Footnotes are not permitted.

METHODS – Nature Cell Biology publishes methods online. The methods section should be provided as a separate Word document, which will be copyedited and appended to the manuscript PDF, and incorporated within the HTML format of the paper.

Methods should be written concisely, but should contain all elements necessary to allow interpretation and replication of the results. As a guideline, Methods sections typically do not exceed 3,000 words. The Methods should be divided into subsections listing reagents and techniques. When citing previous methods, accurate references should be provided and any alterations should be noted. Information must be provided about: antibody dilutions, company names, catalogue numbers and clone numbers for monoclonal antibodies; sequences of RNAi and cDNA probes/primers or company names and catalogue numbers if reagents are commercial; cell line names, sources and information on cell line identity and authentication. Animal studies and experiments involving human subjects must be reported in detail, identifying the committees approving the protocols. For studies involving human subjects/samples, a statement must be included confirming that informed consent was obtained. Statistical analyses and information on the reproducibility of experimental results should be provided in a section titled “Statistics and Reproducibility”.

All Nature Cell Biology manuscripts submitted on or after March 21 2016 must include a Data availability statement as a separate section after Methods but before references, under the heading "Data Availability”. . For Springer Nature policies on data availability see http://www.nature.com/authors/policies/availability.html; for more information on this particular policy see http://www.nature.com/authors/policies/data/data-availability-statements-data-citations.pdf. The Data availability statement should include:

• Accession codes for primary datasets (generated during the study under consideration and designated as "primary accessions") and secondary datasets (published datasets reanalysed during the study under consideration, designated as "referenced accessions"). For primary accessions data should be made public to coincide with publication of the manuscript. A list of data types for which submission to community-endorsed public repositories is mandated (including sequence, structure, microarray, deep sequencing data) can be found here http://www.nature.com/authors/policies/availability.html#data.

• Unique identifiers (accession codes, DOIs or other unique persistent identifier) and hyperlinks for datasets deposited in an approved repository, but for which data deposition is not mandated (see here for details http://www.nature.com/sdata/data-policies/repositories).

• At a minimum, please include a statement confirming that all relevant data are available from the authors, and/or are included with the manuscript (e.g. as source data or supplementary information), listing which data are included (e.g. by figure panels and data types) and mentioning any restrictions on availability.

• If a dataset has a Digital Object Identifier (DOI) as its unique identifier, we strongly encourage including this in the Reference list and citing the dataset in the Methods.

We recommend that you upload the step-by-step protocols used in this manuscript to protocols.io. More details can found at https://www.protocols.io/help/publish-articles.

DISPLAY ITEMS – main display items are limited to 6-8 main figures and/or main tables for Articles, Resources, Technical Reports; and 5 main figures and/or main tables for Letters. For Supplementary Information see below.

FIGURES – Colour figure publication costs $600 for the first, and $300 for each subsequent colour figure. All panels of a multi-panel figure must be logically connected and arranged as they would appear in the final version. Unnecessary figures and figure panels should be avoided (e.g. data presented in small tables could be stated briefly in the text instead).

All imaging data should be accompanied by scale bars, which should be defined in the legend.
Cropped images of gels/blots are acceptable, but need to be accompanied by size markers, and to retain visible background signal within the linear range (i.e. should not be saturated). The boundaries of panels with low background have to be demarked with black lines. Splicing of panels should only be considered if unavoidable, and must be clearly marked on the figure, and noted in the legend with a statement on whether the samples were obtained and processed simultaneously. Quantitative comparisons between samples on different gels/blots are discouraged; if this is unavoidable, it should only be performed for samples derived from the same experiment with gels/blots were processed in parallel, which needs to be stated in the legend.

Figures should be provided at approximately the size that they are to be printed at (single column is 86 mm, double column is 170 mm) and should not exceed an A4 page (8.5 x 11"). Reduction to the scale that will be used on the page is not necessary, but multi-panel figures should be sized so that the whole figure can be reduced by the same amount at the smallest size at which essential details in each panel are visible. In the interest of our colour-blind readers we ask that you avoid using red and green for contrast in figures. Replacing red with magenta and green with turquoise are two possible colour-safe alternatives. Lines with widths of less than 1 point should be avoided. Sans serif typefaces, such as Helvetica (preferred) or Arial should be used. All text that forms part of a figure should be rewritable and removable.

We accept files from the following graphics packages in either PC or Macintosh format:

- For line art, graphs, charts and schematics we prefer Adobe Illustrator (.AI), Encapsulated PostScript (.EPS) or Portable Document Format (.PDF). Files should be saved or exported as such directly from the application in which they were made, to allow us to restyle them according to our journal house style.

- We accept PowerPoint (.PPT) files if they are fully editable. However, please refrain from adding PowerPoint graphical effects to objects, as this results in them outputting poor quality raster art. Text used for PowerPoint figures should be Helvetica (preferred) or Arial.

- We do not recommend using Adobe Photoshop for designing figures, but we can accept Photoshop generated (.PSD or .TIFF) files only if each element included in the figure (text, labels, pictures, graphs, arrows and scale bars) are on separate layers. All text should be editable in 'type layers' and line-art such as graphs and other simple schematics should be preserved and embedded within 'vector smart objects' - not flattened raster/bitmap graphics.

- Some programs can generate Postscript by 'printing to file' (found in the Print dialogue). If using an application not listed above, save the file in PostScript format or email our Art Editor, Allen Beattie for advice (a.beattie@nature.com).

Regardless of format, all figures must be vector graphic compatible files, not supplied in a flattened raster/bitmap graphics format, but should be fully editable, allowing us to highlight/copy/paste all text and move individual parts of the figures (i.e. arrows, lines, x and y axes, graphs, tick marks, scale bars etc.). The only parts of the figure that should be in pixel raster/bitmap format are photographic images or 3D rendered graphics/complex technical illustrations.

All placed images (i.e. a photo incorporated into a figure) should be on a separate layer and independent from any superimposed scale bars or text. Individual photographic images must be a minimum of 300+ DPI (at actual size) or kept constant from the original picture acquisition and not decreased in resolution post image acquisition. All colour artwork should be RGB format.

FIGURE LEGENDS – must not exceed 350 words for each figure to allow fit on a single printed NCB page together with the figure. They must include a brief title for the whole figure, and short descriptions of each panel with definitions of the symbols used, but without detailing methodology.

TABLES – main tables should be provided as individual Word files, together with a brief title and legend. For supplementary tables see below.

SUPPLEMENTARY INFORMATION – Supplementary information is material directly relevant to the conclusion of a paper, but which cannot be included in the printed version in order to keep the manuscript concise and accessible to the general reader. Supplementary information is an integral part of a Nature Cell Biology publication and should be prepared and presented with as much care as the main display item, but it must not include non-essential data or text, which may be removed at the editor's discretion. All supplementary material is fully peer-reviewed and published online as part of the HTML version of the manuscript. Supplementary Figures and Supplementary Notes are appended at the end of the main PDF of the published manuscript.

Supplementary items should relate to a main text figure, wherever possible, and should be mentioned sequentially in the main manuscript, designated as Supplementary Figure, Table, Video, or Note, and numbered continuously (e.g. Supplementary Figure 1, Supplementary Figure 2, Supplementary Table 1, Supplementary Table 2 etc.).

Unprocessed scans of all key data generated through electrophoretic separation techniques need to be presented in a supplementary figure that should be labelled and numbered as the final supplementary figure, and should be mentioned in every relevant figure legend. This figure does not count towards the total number of figures and is the only figure that can be displayed over multiple pages, but should be provided as a single file, in PDF or TIFF format. Data in this figure can be displayed in a relatively informal style, but size markers and the figures panels corresponding to the presented data must be indicated.

The total number of Supplementary Figures (not including the “unprocessed scans” Supplementary Figure) should not exceed the number of main display items (figures and/or tables (see our Guide to Authors and March 2012 editorial http://www.nature.com/ncb/authors/submit/index.html#suppinfo; http://www.nature.com/ncb/journal/v14/n3/index.html#ed). No restrictions apply to Supplementary Tables or Videos, but we advise authors to be selective in including supplemental data.

Each Supplementary Figure should be provided as a single page and as an individual file in one of our accepted figure formats and should be presented according to our figure guidelines (see above). Supplementary Tables should be provided as individual Excel files. Supplementary Videos should be provided as .avi or .mov files up to 50 MB in size. Supplementary Figures, Tables and Videos much be accompanied by a separate Word document including titles and legends.

GUIDELINES FOR EXPERIMENTAL AND STATISTICAL REPORTING

REPORTING REQUIREMENTS – We are trying to improve the quality of methods and statistics reporting in our papers. To that end, we are now asking authors to complete a reporting summary that collects information on experimental design and reagents. The Reporting Summary can be found here https://www.nature.com/documents/nr-reporting-summary.pdf)If you would like to reference the guidance text as you complete the template, please access these flattened versions at http://www.nature.com/authors/policies/availability.html.

STATISTICS – Wherever statistics have been derived the legend needs to provide the n number (i.e. the sample size used to derive statistics) as a precise value (not a range), and define what this value represents. Error bars need to be defined in the legends (e.g. SD, SEM) together with a measure of centre (e.g. mean, median). Box plots need to be defined in terms of minima, maxima, centre, and percentiles. Ranges are more appropriate than standard errors for small data sets. Wherever statistical significance has been derived, precise p values need to be provided and the statistical test used needs to be stated in the legend. Statistics such as error bars must not be derived from n<3. For sample sizes of n<5 please plot the individual data points rather than providing bar graphs. Deriving statistics from technical replicate samples, rather than biological replicates is strongly discouraged. Wherever statistical significance has been derived, precise p values need to be provided and the statistical test stated in the legend.

Information on how many times each experiment was repeated independently with similar results needs to be provided in the legends and/or Methods for all experiments, and in particular wherever representative experiments are shown.

We strongly recommend the presentation of source data for graphical and statistical analyses as a separate Supplementary Table, and request that source data for all independent repeats are provided when representative experiments of multiple independent repeats, or averages of two independent experiments are presented. This supplementary table should be in Excel format, with data for different figures provided as different sheets within a single Excel file. It should be labelled and numbered as one of the supplementary tables, titled “Statistics Source Data”, and mentioned in all relevant figure legends.

--------- Please don't hesitate to contact NCB@nature.com should you have queries about any of the above requirements ---------

Version 1:

Decision Letter:

Our ref: NCB-A57418A

1st July 2026

Dear Dr. Nadler,

Thank you for submitting your revised manuscript "Quantification of lipid sorting during clathrin-mediated endocytosis" (NCB-A57418A). It has now been seen by the original referees and their comments are below. The reviewers find that the paper has been strengthened in revision, and therefore we'll be happy in principle to publish it in Nature Cell Biology, pending minor revisions to satisfy the referees' final requests and to comply with our editorial and formatting guidelines.

Please note that the current version of your manuscript is in a PDF format. Could you please email us a copy of the file in an editable format (Microsoft Word) as we cannot proceed with PDFs at this stage? Thank you in advance.

We are now performing detailed checks on your paper and will send you a checklist detailing our editorial and formatting requirements in a couple of weeks. Please do not upload the final materials and make any revisions until you receive this additional information from us.

Thank you again for your interest in Nature Cell Biology. Please do not hesitate to contact me if you have any questions.

Sincerely,

Melina Casadio, PhD
Senior Editor, Nature Cell Biology
ORCID ID: https://orcid.org/0000-0003-2389-2243

Reviewer #1 (Remarks to the Author):

I commend the authors on their (typically) rigorous and thorough revision. The new experiments add quite a lot to the manuscript, addressing the main caveat (probe behavior) directly and quantitatively. I was supportive of this story originally and now am convinced that it should be published without delay. Congrats to the authors on important and rigorous work!

Reviewer #2 (Remarks to the Author):

I appreciate the revision efforts, which clearly addressed my previous comments.

Reviewer #3 (Remarks to the Author):

We thank the authors for their thorough response and for dedicating significant effort to addressing our previous comments. The additional experimental data and clarifications have markedly strengthened the manuscript. However, there remain a few final points that we believe require minor text adjustments and clarification prior to publication.

1. PIP2 and PS trafficking point: Because lipids like PIP2 and PS template the initiation of CME sites, it remains highly possible that CME actively regulates CME site plasma membrane distribution. These specific species were not explicitly tested in this study. However, the manuscript text frequently refers to “lipids” in a general sense. To avoid confusing the reader, it is crucial to explicitly state the caveat that the specific probes evaluated here may not reflect the trafficking behavior of these key regulatory lipids.

The authors modified the text to read: “We note that our findings here do not preclude sorting of individual lipids during clathrin-independent endocytic processes, with caveolae-mediated endocytosis proposed as one possible lipid sorting mechanism 23, but these mechanisms are likely minor components in the total transport volume of lipids between the plasma membrane and other organelle membranes.”

We feel a more precise and appropriate clarification for the reader would be: “While our results indicate that clathrin-mediated endocytosis exhibits only limited selectivity for cytoplasmic leaflet lipids, our analysis was not intended to comprehensively assess all lipid species. In particular, we excluded PIP2 and PS because they are well-established regulators of CME, and their inclusion would confound our ability to test whether the CME machinery itself drives the sorting of PIP2 and PS. Consequently, we cannot exclude the possibility that CME selectively regulates the distribution of these specific lipid species.”

2. Geometric modeling point: We were expecting estimates of the expected differences in maximal lipid numbers observed in the inner vs outer leaflets for a select number of relevant lipids, and apologize for the lack of specificity in the request. While it would have been nice to see, we are content with this topic being omitted from the manuscript, given the strength of the existing modeling.

3. Ionomycin biological perturbation: We thank the authors for their diligent attempt to address our hypothesis using acute membrane scrambling. Although the rapid disassembly of clathrin-coated pits (CCPs) prevented a direct test of the original partitioning hypothesis, documenting this structural dependence provides a valuable and interesting record for the endocytosis literature.

4. Receptor density experiment: We appreciate the rigorous effort that went into executing this new cargo density correlation analysis. To better match the definitive nature of the data, we recommend adjusting the title of Extended Data 7 to read: “Extended Data 7: The receptor density in late-stage CCPs does not predict lipid pit partitioning. “

5. Finally, there are a few typographical errors in the text that must be corrected during final production: For example: “Dymanin2” should be corrected to “Dynamin2” in the Acknowledgments; “Life cell imaging” should be “live cell imaging” in Extended Data 8; and “diplamitoylphosphatidylcholine” should be “dipalmitoylphosphatidylcholine” in Reference 38.

INTERNAL USE ONLY AIP modules required: ["Clinical","Life Science"]

Version 2:

Decision Letter:

Dear Dr Nadler,

I am pleased to inform you that your manuscript, "Quantification of lipid sorting during clathrin-mediated endocytosis", has now been accepted for publication in Nature Cell Biology.

Thank you for sending us the final manuscript files to be processed for print and online production, and for returning the manuscript checklists and other forms. Your manuscript will now be passed to our production team who will be in contact with you if there are any questions with the production quality of supplied figures and text.

Over the next few weeks, your paper will be copyedited to ensure that it conforms to Nature Cell Biology style. Once your paper is typeset, you will receive an email with a link to choose the appropriate publishing options for your paper and our Author Services team will be in touch regarding any additional information that may be required.

After the grant of rights is completed, you will receive a link to your electronic proof via email with a request to make any corrections within 48 hours. If, when you receive your proof, you cannot meet this deadline, please inform us at rjsproduction@springernature.com immediately.

You will not receive your proofs until the publishing agreement has been received through our system.

Due to the importance of these deadlines, we ask that you please let us know now whether you will be difficult to contact over the next month. If this is the case, we ask you provide us with the contact information (email, phone and fax) of someone who will be able to check the proofs on your behalf, and who will be available to address any last-minute problems.

If you have any questions about our publishing options, costs, Open Access requirements, or our legal forms, please contact ASJournals@springernature.com

Once your paper has been scheduled for online publication, the Nature press office will be in touch to confirm the details. An online order form for reprints of your paper is available at https://www.nature.com/reprints/author-reprints.html. All co-authors, authors' institutions and authors' funding agencies can order reprints using the form appropriate to their geographical region.

Publication is conditional on the manuscript not being published elsewhere and on there being no announcement of this work to any media outlet until the online publication date in Nature Cell Biology.

You may wish to make your media relations office aware of your accepted publication, in case they consider it appropriate to organize some internal or external publicity. Once your paper has been scheduled you will receive an email confirming the publication details. This is normally 3-4 working days in advance of publication. If you need additional notice of the date and time of publication, please let the production team know when you receive the proof of your article to ensure there is sufficient time to coordinate. Further information on our embargo policies can be found here: https://www.nature.com/authors/policies/embargo.html

Please note that *Nature Cell Biology* is a Transformative Journal (TJ). Authors may publish their research with us through the traditional subscription access route or make their paper immediately open access through payment of an article-

processing charge (APC). Authors will not be required to make a final decision about access to their article until it has been accepted. Find out more about Transformative Journals

Authors may need to take specific actions to achieve compliance with funder and institutional open access mandates. If your research is supported by a funder that requires immediate open access (e.g. according to Plan S principles or the NIH public access policy) then you should select the gold OA route, and we will direct you to the compliant route where possible. Because authors warrant under our subscription licensing terms that they haven’t committed to licensing any version of their article under a licence inconsistent with the terms of our agreement – including the applicable embargo period – publication under the subscription model isn’t suitable for authors whose funders require no embargo.

To assist our authors in disseminating their research to the broader community, our SharedIt initiative provides you with a unique shareable link that will allow anyone (with or without a subscription) to read the published article. Recipients of the link with a subscription will also be able to download and print the PDF.

If your paper includes color figures, please be aware that in order to help cover some of the additional cost of four-color reproduction, Nature Portfolio charges our authors a fee for the printing of their color figures. Please contact our offices for exact pricing and details.

As soon as your article is published, you will receive an automated email with your shareable link.

If you have not already done so, we strongly recommend that you upload the step-by-step protocols used in this manuscript to protocols.io (https://protocols.io), an open online resource that allows researchers to share their detailed experimental know-how. All uploaded protocols are made freely available and are assigned DOIs for ease of citation. Protocols and Nature Portfolio journal papers in which they are used can be linked to one another, and this link is clearly and prominently visible in the online versions of both. Authors who performed the specific experiments can act as primary authors for the Protocol as they will be best placed to share the methodology details, but the Corresponding Author of the present research paper should be included as one of the authors. By uploading your Protocols onto protocols.io, you are enabling researchers to more readily reproduce or adapt the methodology you use, as well as increasing the visibility of your protocols and papers. You can also establish a dedicated workspace to collect your lab Protocols. Further information can be found at https://www.protocols.io/help/publish-articles.

You can use a single sign-on for all your accounts, view the status of all your manuscript submissions and reviews, access usage statistics for your published articles and download a record of your refereeing activity for the Nature Portfolio.

Nature Cell Biology encourages authors presenting evidence for cell, biological, molecular, and genetic interactions to consider communicating these findings using Biofactoid (https://biofactoid.org/). This tool helps users share a searchable representation of interactions (e.g. binding, gene expression, post-translational modification) between genes, gene products, or chemicals. Information added to Biofactoid, with author attribution, is shared on social media and public databases, such as Pathway Commons, where it can be discovered and analyzed in the context of a large and growing corpus of knowledge.

Please feel free to contact us if you have any questions.

With kind regards,

Melina Casadio, PhD
Senior Editor, Nature Cell Biology
ORCID ID: https://orcid.org/0000-0003-2389-2243

Click here if you would like to recommend Nature Cell Biology to your librarian
http://www.nature.com/subscriptions/recommend.html#forms

** Visit the Springer Nature Editorial and Publishing website at www.springernature.com/editorial-and-publishing-jobs for more information about our career opportunities. If you have any questions please click here.**

Open Access This Peer Review File is licensed under a Creative Commons Attribution 4.0 International License, which permits use, sharing, adaptation, distribution and reproduction in any medium or format, as long as you give appropriate credit to the original author(s) and the source, provide a link to the Creative Commons license, and indicate if changes were made.
In cases where reviewers are anonymous, credit should be given to 'Anonymous Referee' and the source.
The images or other third party material in this Peer Review File are included in the article's Creative Commons license, unless indicated otherwise in a credit line to the material. If material is not included in the article's Creative Commons license and your intended use is not permitted by statutory regulation or exceeds the permitted use, you will need to obtain permission directly from the copyright holder.
To view a copy of this license, visit https://creativecommons.org/licenses/by/4.0/

Quantification of lipid sorting during clathrin-mediated endocytosis

Corresponding Authors: Prof. Dr. Alf Honigmann, Dr. André Nadler

General remarks:
We thank all reviewers for their comments, which provided helpful and constructive feedback. We believe that the revisions made the manuscript substantially stronger.
We have addressed all points experimentally that were feasible in the time frame of the revision. We have acquired extensive new data, which are shown in the 3 new and several expanded Extended Data figures. The main improvements of the manuscript are:

- We provide and reference extensive methodological controls, both from our previous lipid imaging papers as well as new data to establish that the effect of the chemical modifications of our lipid probes are approximately equivalent to that of one additional double bond.
- We assess the interplay of lipid asymmetry and clathrin-mediated endocytosis in depth and show that the asymmetric lipid distribution across the plasma membrane is actually needed for clathrin coated pit stability.
- We show that the density of individual protein cargoes (receptors) in clathrin coated pits does not affect lipid partitioning

All new datasets align with our conclusions, as discussed in detail below in our responses to the individual reviewer queries.

The following section outlines the structure of this document and the revised version, and gives an overview of the restructuring of the original manuscript version, which was required to accommodate the new datasets.

1. Full reviewer comments are provided together with our point-by-point answers to maintain the context. Key results are included here as revised / new figures or reviewer figures, and the primary conclusion from a new experiment/dataset is highlighted. Our answers to individual reviewer comments are highlighted in blue to enhance clarity.

2. We have added a new main text Figure:
Supplementary figure 2 is now main text figure 5

3. The following main text figures have been revised:
Main text Figure 1
Main text figure 4

4. The following ED figures have been revised:
Extended Data 1 and 3 were combined to the new Extended Data 2;
Extended Data 2 and 4 were combined to the new Extended Data 3;
Extended Data 5 and 6 were combined to the new Extended Data 4;
Extended Data 7 and 8 were combined to the new Extended Data 5;
Extended Data 9 is now the new Extended Data 6;
Supplementary Figure 1 is now the new Extended Data 9;

5. The following new figures have been added to the Extended Data:
Extended Data 1, Extended Data 7, Extended Data 8

6. All textual changes in the revised manuscript have been highlighted in yellow.

<u>Answers to specific reviewer questions:</u>

Reviewer #1 (Remarks to the Author):

In this manuscript, Lennartz et al present a compelling study of lipid partitioning into clathrin-coated pits. It addresses a big, open question that many have approached but none have carefully investigated – is there lipid selectivity in the clathrin-mediated endocytosis? This study answers the question - quite definitively - with advanced imaging of carefully designed chemical probes combined with rigorous analysis and modeling. The latter is essential and non-trivial: clathrin pits are 3D objects usually imaged in 2D projections that dramatically convolute shape and lateral organization (cartoon in Fig 2D is nice: makes an important, quantitatively accurate point). The approach here is smart and rigorous, relying on EM images of pits to model their structures and the resulting effect on lipid imaging.

The short answer to the big question is that there is almost no lateral lipid selectivity, with the observed trends fitting well to lipid transbilayer distributions. That is, there is less outer leaflet area in a CCP, so lipids of the outer leaflet should be generically slightly excluded. This may sound like a 'negative' result, but it isnt. 'No selectivity' is still an interesting – and quite surprising – effect. It is to the authors credit that they emphasize the clear major conclusion rather than trying to hype up small differences.

Overall, I find the work rigorous, interesting, and beautifully presented. My only significant suggestions are for the Discussion: I point out a couple of specifics, though I feel that the authors could expand on other possibilities (eg selectivity of non-clathrin-mediated vesicular transport).

We thank reviewer #1 for the in-depth and very positive assessment of our manuscript. We agree with the notion that this is not a "negative" result. We are just at the very beginning of understanding mechanism of species-specific lipid sorting into organelle membranes, which means that we have to map selective and non-selective processes to understand how the cellular lipid flux network is organized and each datapoint helps us in this regard. Please see below for our specific responses.

The manuscript is very nicely written: clear, concise, and direct. But the **Discussion is too short**, omitting some important points:
a. the strong correlation to lipid Tm is probably also explainable by asymmetry… but the first order effect is exactly what the authors were testing (ie phase enrichment), just backwards from expectation. I think this should at least be discussed.

We agree with reviewer #1 and have expanded the discussion of this aspect, also in the light of new experiments that we have conducted. Briefly, we tried to perturb lipid asymmetry and measure the consequences for lipid partitioning into clathrin coated pits. To avoid long-term cellular adaptations upon genetic perturbations (for instance, TMEM30A KD causes broad remodeling of the cellular lipidome and proteome and results in a major change of plasma membrane biophysics), we induced plasma membrane scrambling using ionomycin generated calcium influx in SK-MEL cells (ED 8A). Calcium activates a subset of endogenous plasma membrane resident scramblases, thus initiating scrambling without the need of genetic perturbations, and at fast time scales (seconds to minutes). Scrambling was monitored by PS exposure (ED 8B). Calcium-induced lipid scrambling lead to a mis-localization of clathrin, fast dissociation of dynamin-2 GFP, and an almost instant block of transferrin uptake (ED 8C - E).

This data suggests that clathrin-coated pits disassemble upon plasma membrane scrambling, with a kinetic that appeared to be faster than the rate of PS externalization, indicating that there is no time-window during which a scrambled plasma membrane lipid composition and regular CCP organization can co-exist (ED 8C-E). To understand whether CCP disassembly is indeed caused by plasma membrane scrambling and not the sudden influx of calcium, we induced calcium influx in HEK293 cells, that have been reported for their low endogenous scramblase activity [1,2]. In HEK293, while we do monitor calcium influx, we could not observe PS exposure to the extracellular leaflet, indicative of the lack of PM scrambling. In ionomycin - treated HEK293 cells, clathrin localization appears unaltered compared to the untreated control. These data are included in the manuscript in new ED Figure 8. It appears – at least in our hands – that CME and lipid asymmetry are too closely linked to be functionally separated.

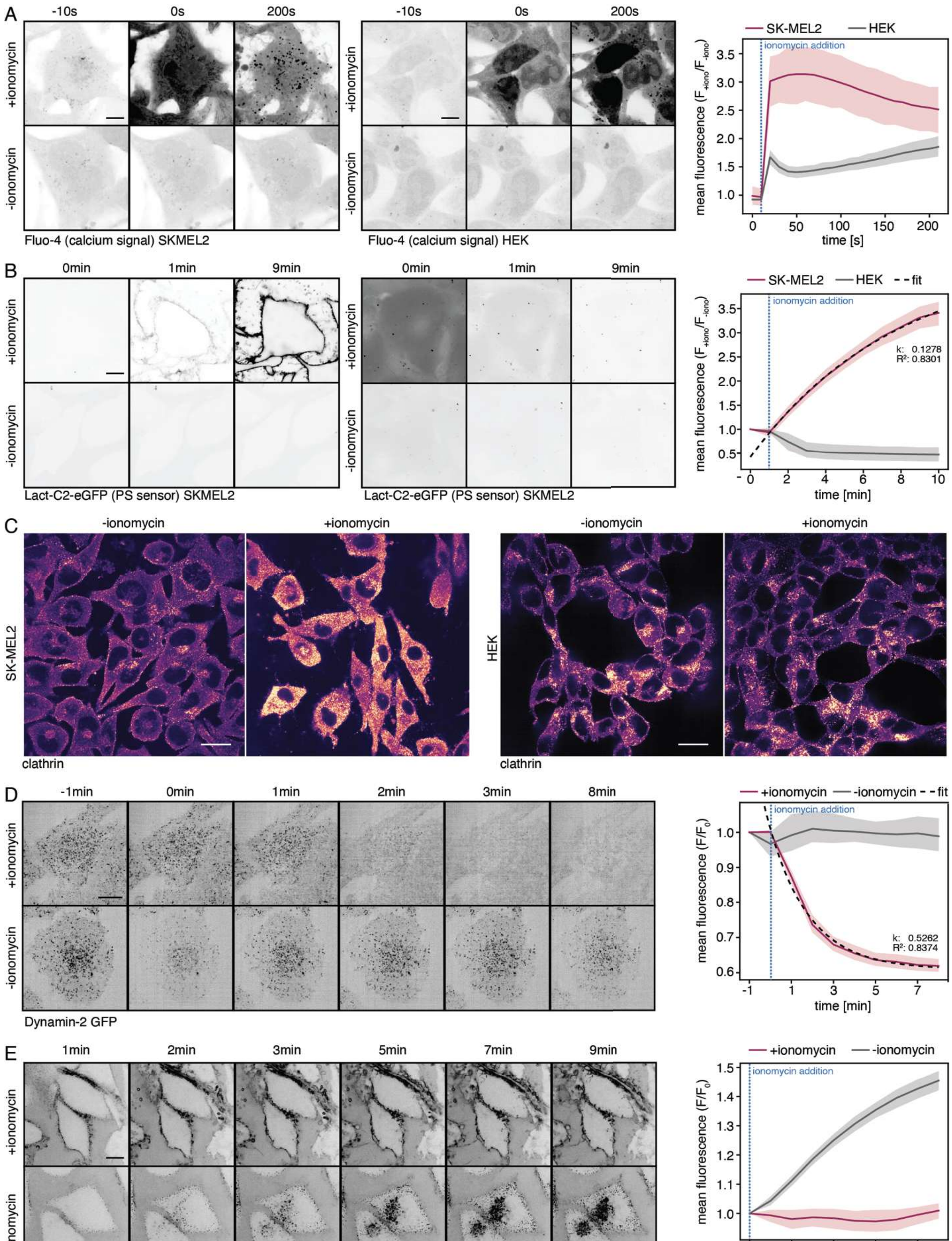
A
-10s
0s
200s
+ionomycin
-ionomycin
Fluo-4 (calcium signal) SKMEL2
Fluo-4 (calcium signal) HEK
SK-MEL2
HEK
ionomycin addition
mean fluorescence (F_{+iono}/F_{-iono})
time [s]
B
0min
1min
9min
Lact-C2-eGFP (PS sensor) SKMEL2
fit
k: 0.1278
R²: 0.8301
time [min]
C
SK-MEL2
HEK
clathrin
D
-1min
0min
1min
2min
3min
8min
Dynamin-2 GFP
+ionomycin
-ionomycin
mean fluorescence (F/F_0)
k: 0.5262
R²: 0.8374
E
1min
2min
3min
5min
7min
9min
Transferrin-AF647 fluorescent conjugate

Extended Data 8: Clathrin-coated pits disassemble upon plasma membrane scrambling. SK-MEL2 or HEK293 cells were treated with 5 μM of the ionophore ionomycin in buffer containing 2.5 mM Ca^{2+}. **A**: Relative cytosolic calcium was monitored during life cell imaging for 200 s at 10 s intervals using the Fluo-4 Calcium Imaging Kit (Invitrogen), signal shown in grey. The average Fluo-4 signal of SK-MEL2 (purple) or HEK293 (grey) cells is plotted against time. The signal is normalized to the mean signal of the control (no ionomycin) across time. The error of the normalized average fluorescent signal is shaded and given as the confidence interval (CI) of 95%. Three independent biological samples were analyzed for each condition, n(total fields of view): 18. **B**: Phosphatidylserine (PS) exposure to the extracellular leaflet of the plasma membrane is monitored as a proxy for plasma membrane scrambling. PS exposure is measured using the PS sensor Lact-C2-eGFP [3] over 10 min at 1 min intervals of SK-MEL2 (purple) or HEK293 (grey) cells. The GFP signal is normalized to the mean signal of the control (no ionomycin) across time. The error of the normalized average fluorescent signal is shaded and given as CI: 95%. Three independent samples were analyzed for each condition, n(total fields of view): 18. The SKMEL trace was fitted (black dotted line) using a simple exponential one phase association model. The rate constant k and R^2 are indicated. **C**: SK-MEL2 (left panel) or HEK293 (right panel) cells were treated with ionomycin for 5 min in buffer containing 2.5mM Ca^{2+} and fixed. Clathrin localization was assessed using immunostaining. Representative images of 3 independent biological replicates are shown, that yielded similar results. **D**: Dynamin-2 of a SK-MEL2 dynamin-2-GFP cell line localization was monitored over 10 min at 1 min intervals of cells treated with (purple) or without (grey) ionomycin at the basal plasma membrane. The mean GFP signal is normalized to the mean signal at time -1 min (relative to ionomycin addition). The error of the normalized average fluorescent signal is shaded and given as CI: 95%. Three independent samples were analyzed for each condition, n(total fields of view per condition): 18. The SKMEL trace was fitted (black dotted line) using a simple exponential one phase dissociation model. The rate constant k and R^2 are indicated. **E**: The uptake of a fluorescently tagged transferrin conjugate was monitored over 10 min at 1 min intervals of cells treated with (purple) or without (grey) ionomycin. The mean fluorescent signal is normalized to the mean signal at time -1 min (relative to ionomycin addition). The error of the normalized average fluorescent signal is shaded and given as CI: 95%. Three independent samples were analyzed for each condition, n(total fields of view): 30. Scale bars: 10μm. Scale bars of ROIs: 1μm.

The corresponding new section in the results now reads:

Despite the experimental challenges associated with perturbing lipid transbilayer composition in living cells, we attempted to investigate the proposed connection between lipid asymmetry and lipid partitioning into CCPs. Since genetic perturbations of the flippase machinery that establishes lipid asymmetry (TMEM30A) result in complex remodeling of the lipidome, proteome and metabolism that go far beyond a simple loss of lipid asymmetry[4,5], we opted for an acute perturbation. To this end, we induced lipid scrambling mediated by the Ca^{2+}-dependent scramblase TMEM16F by treating both SK-MEL2 cells, which express TMEM16F and HEK293 cells, which have low endogenous scramblase activity [1,2] with ionomycin in a calcium containing buffer. Both cell types responded with robust calcium influx (ED 8A), but phosphatidylserine (PS) externalization was observed only in SK-MEL2 cells (ED 8B). Thus, by comparing both cell lines we wanted to separate the effects of elevated Ca^{2+} levels from the scrambling of plasma membrane lipids. However, we found that upon ionomycin treatment clathrin was rapidly released from the plasma membrane in SK-MEL2 but not in HEK293 cells (ED 8C). Similarly, the plasma membrane bound pool of Dynamin-2-GFP was fully released 3 min after ionomycin addition. Indeed, late-stage CCPs disassembled with a ~4 times faster kinetic (rate constant $k_{(dynamin\ disassembly)} \pm$ CI95: 0.5262 ± 0.0691) compared to PS externalization ($k_{(PS\ assembly)} \pm$ CI95: 0.1278 ± 0.0564) (ED 8D). In line with this data, we found that ionomycin also fully blocks transferrin uptake in SK-MEL-2 cells (ED 8E). Thus, experimentally it was not possible to monitor lipid partitioning into CCPs after plasma membrane scrambling.
Together, our data indicates that the asymmetric lipid composition of the plasma membrane is a prerequisite for CME and that lipid asymmetry and CME are too closely linked to be functionally separated. This finding is in line with previous studies [6] and the reported role of lipids, such as $PI(4,5,)P_2$ and PS, to facilitate the binding of multiple proteins of the CME machinery [6,7].

Thus, despite our best efforts, we were not able to disentangle the effect of lipid steady-state asymmetry from a putative effect purely caused by biophysical properties of lipids by experimental means. It may very well be that this is a fundamental problem since lipid asymmetry requires the mobilization of lipids from the bulk bilayer by lipid translocases – and the transition states of the corresponding reaction coordinates will depend on the biophysical properties of the substrate lipid species.
With regard to the conclusion of this manuscript, we have adapted the discussion to account for this uncertainty. Nevertheless, we do think that this is a case were Ockham's razor applies. The respective sections now read as follows:

In addition, we found a strong correlation between melting temperature and CCP partitioning, with high melting temperature lipids partitioning more strongly into CCPs. This is in contrast to the expected depletion of high melting temperature lipids into curved membranes, based on previous reports [8–11] and the biophysical properties of a curved membrane [12,13]. Thus, it is likely that the observed lipid partitioning trends are not explained by classical L_o/L_d phase separation, which would result in the opposite partitioning behavior. In this context, it is an intriguing observation that melting temperature and lipid asymmetry are positively correlated, at least for the glycerophospholipids (ED 10). We have previously observed similar trends for the rates of non-vesicular lipid transport and metabolism for different PC species [3]. The initial step in all three processes is the mobilization/extraction of a lipid from the bulk bilayer by a protein (lipid translocases, lipid transfer proteins, lipid metabolizing enzymes). It is a compelling speculation that lipid mobilization, which should depend heavily on lipid biophysical properties, could be the

rate limiting step in all cases. If this were the case, lipid biophysical properties would be a key determinant of trans-bilayer, intra- and inter-organelle lipid distribution.

b. Relatedly, it is probably an important caveat to mention that the while the analogs used here are indeed “near-native”, the diazerene moiety seems likely to perturb acyl chain packing, in the same direction that a double-bond would. As the authors surely recognize, its an important caveat for their rafty interpretations (or conspicuous lack thereof).

This is correct. Indeed, we have carried out a quite comprehensive biophysical characterization of these probes in our recent story (Iglesias-Artola et al., Nature, 646, 474-482, 2025; see Extended Data Figure 1 and Supplementary Figure 1, reproduced below as Reviewer Figure 1). The result was the one reviewer #1 anticipated, the diazirine lipids behave as a native lipid featuring a single additional double bond across all analyzed probes. Given that the internal angle of the 3-membered diazirine ring is 60°, the resulting kink in the fatty acid chain resembles the one induced by a double bond. We have discussed this issue in our previous manuscript as follows:
“There are two main limitations with our current approach to measuring lipid transport. First, our data indicate that the C16 bifunctional fatty acid used in this study resembles a native monounsaturated fatty acid (C16:1). It is therefore unlikely that fully saturated lipids can be faithfully mimicked using the bifunctional fatty acid that we used in the current study. To an extent, it may be possible to mitigate this by placing the diazirine moiety at either end of fatty acid rather than in the centre of the aliphatic chain (…).”
Thus, we are very aware of the issue that the function & localization of fully saturated lipids is highly likely only inadequately captured by bifunctional lipid probes, we are discussing it in our published work & are suggesting possible mitigation strategies.
For the purpose of the present manuscript, we have:

(i) teamed up again with the Hof lab to biophysically characterize the probes that were not part of the initial Iglesias-Artola et al. story (using Laurdan temperature scans and FRET assays of nanodomain formation in model membranes). We (unsurprisingly) find that the trend of the diazirine group resembling the effect of one additional double bond holds up for the additional probes as well. These data are included in the manuscript in new Extended Data 1, also shown below.
(ii) We have added a statement to the text that clarifies that the melting temperatures and intrinsic curvatures are the values for the native lipids, which will be shifted in a pretty uniform way due to the presence of the diazirine group.

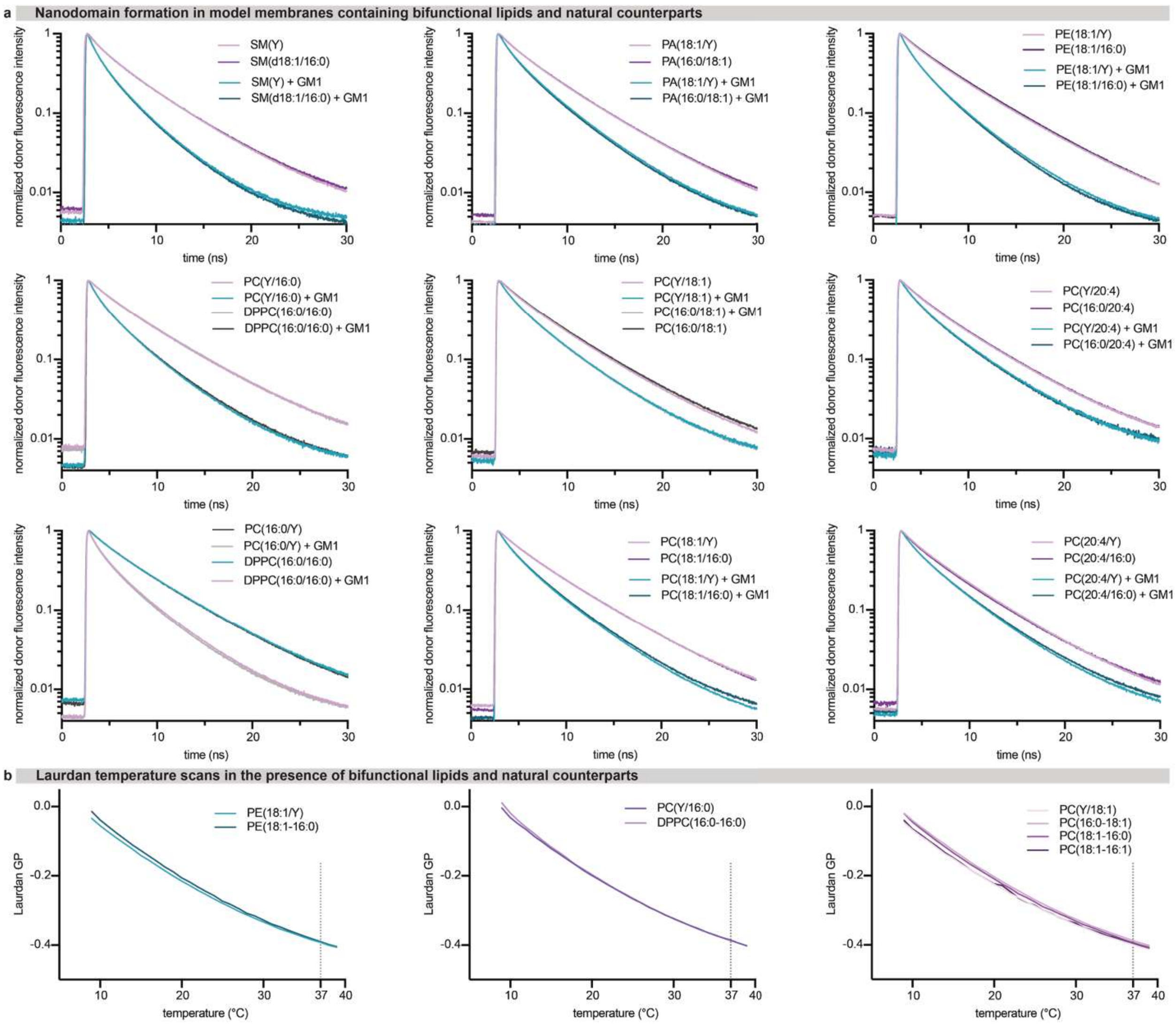

Reviewer Figure 1: Biophysical characterization of bifunctional lipid probes. **a.** Bifunctional lipid probes were characterized for their biophysical properties. Formation of ganglioside nanodomains leading to faster deexcitation of Bodipy-FL-GM1 donors via FRET is unaffected by replacing the closest structurally related native lipid with the bifunctional variant for all probes. **b.** Laurdan temperature scans indicate that the biophysical implications of introducing a bifunctional fatty acid are small.

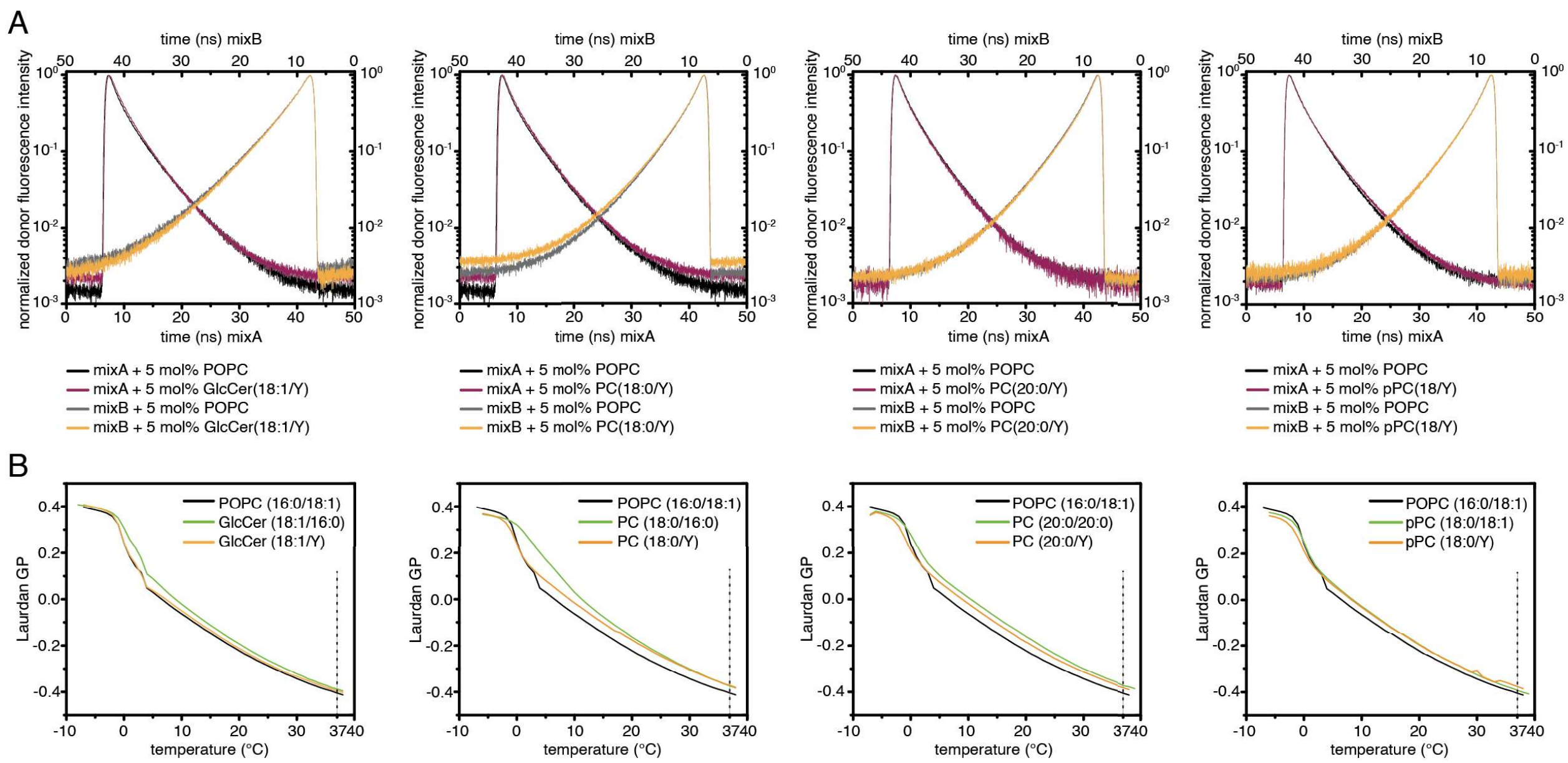

Extended Data1: Biophysical characterization of bifunctional lipid probes by comparison to native counterparts. A: Formation of ganglioside nanodomains leading to faster deexcitation of Bodipy-FL-GM1 donors via FRET is unaffected by replacing the closest structurally related native lipid with the bifunctional variant for all probes. Lipids tested included GlcCer(18:1/Y), PC(18:0/Y), PC(20:0/Y), and pPC(18/Y). MixA: DOPC/Cholesterol/SM (65/25/10 in mol%) containing lipid nanodomains with the average radius of 9 nm and occupying 45% [14] of the membrane surface, mixB: DOPC/Cholesterol/SM (75/25/10 in mol%) + 4% GM1 with the nanodomains of the radius 99 nm and occupying 50% [15] of the membrane surface. Data was acquired for 1 replicate, n(vesicles per condition): at least 5. **B:** Laurdan temperature scans indicate that biophysical implications of introducing a bifunctional fatty acid are small. Equivalent FRET and Laurdan experiments for the additional lipids used in this study have been previously reported by Iglesias-Artola et al. [3]. Data was acquired for 1 replicate.

We have added two sections to the manuscripts that summarize the results on probe characterization, which now reads as follows:

To quantify the role of CME for sorting lipids at the plasma membrane, we optimized our recently developed lipid imaging workflow [16] for super-resolution STED microscopy. The lipid imaging approach relies on near-native bifunctional lipids, which are modified with a diazirine and alkyne moiety. The diazirine can be photochemically crosslinked to neighboring biomolecules, while the alkyne is used for labelling by click chemistry. These probes closely resemble native lipids with regard to their metabolism and biophysical properties. The combined effect of the modifications is approximately equivalent to the introduction of one additional double bond [3,17,18] (ED 1). Briefly, bifunctional lipids were loaded to the outer leaflet of the plasma membrane and fixed via subsequent UV-light- and chemical crosslinking (Figure 1A).

And:

UV-crosslinking and fixative addition were completed within < 10 seconds, which allowed to capture snapshots of CCPs at different maturation stages [19], under conditions that do not affect major membrane trafficking pathways [17].

We have also included that the correlations are indicating the melting temperatures of native lipid counterparts in the corresponding figure legend (shown in our answer to the next point below) and have included a sentence in the main text to clarify the context:

These values correspond to the (saturated) native counterparts of the lipid probes used here. The actual values of the bifunctional probes are likely shifted to slightly lower melting temperatures, because the diazirine group acts as one additional double bond (ED 1) [3].

c. My only major gripe is with the correlations to expected lipid physical properties. Again, as with the rest of the paper, its careful, rigorous analysis. But the presentation gives the impression that the lipid shapes, melting temps, etc are actually known, **which they are not for these analogs**. It is probably the case that the trends to the native lipids are generally maintained, since the tags are always similar. But Fig 4C really implies that these are real values, not inferred or proxies. I don't have a recommendation for how to be more circumspect with the data presentation, but minimally the caveat should be discussed

Please also see our answer to the related point directly above, we (i) discuss the effect of the introduction of the diazirine now and (ii) provide new data both in this manuscript and our previous work that the label indeed results in a homogenous shift compared to native lipids, most closely representing a single double bond. We have also adapted the figure legend to point out the approximation between the biophysical properties of native lipids used as proxies for the here used bifunctional lipid probes (see below):

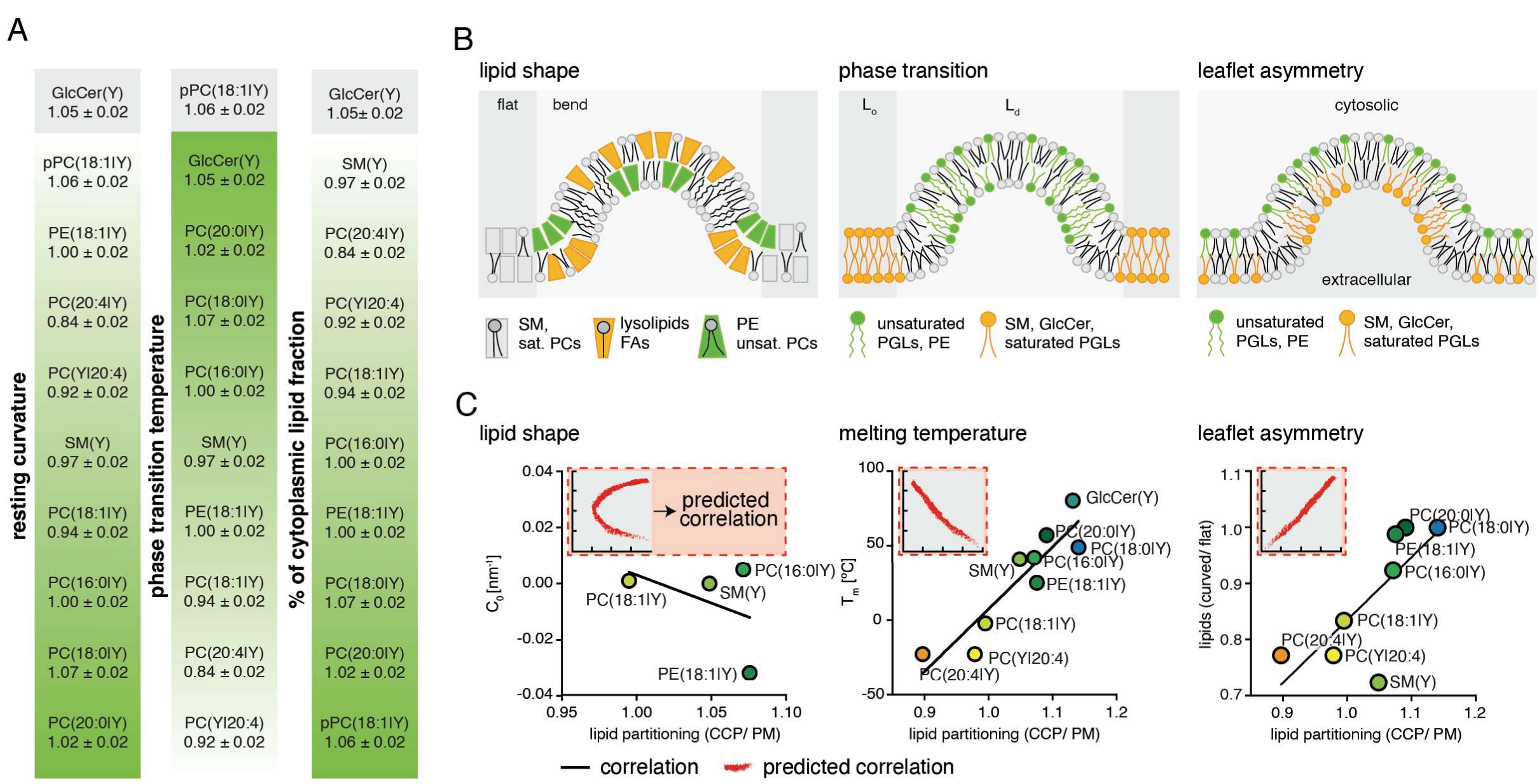

Figure 4: Leaflet asymmetry is sufficient to explain lipid partitioning into clathrin-coated pits. A: Bifunctional lipids were ordered according to the resting curvature, melting temperature, and transbilayer distribution (cytoplasmic/ exoplasmic) of their native lipid counterparts based on reported values from the literature or estimates based on close structural analogs. B: Schemes of possible mechanisms of lipid sorting into clathrin-coated pits are shown. L_d – liquid disordered phase; L_o – liquid-ordered phase. C: The median of absolute lipid densities of late-stage pits is plotted against the resting curvature (C_0), melting temperature (T_m), and the ratio of estimated lipids in a vesicle over a flat membrane of the same surface area determined from their respective native lipid counterparts. The values plotted can be found in the supplement 2 - 4. Only the lipids are plotted for which the corresponding values of C_0, T_m, and lipid asymmetry were reported in the literature. The red line is a sketch and indicates the expected trend of the correlation according to the model.

Minor wording issues:

1. Last sentence of abstract is odd. Are the authors intending to highlight their technology as a major advance? That's not obvious from the wording and its not clear why this sentence occupies this position of importance.

Agreed. We removed the sentence.

2. The orange images representing lipid signal are a bit unclear to me: is it possible to estimate how many molecules a given intensity level represents? Maybe this is too difficult or irrelevant, but its not clear whether the bright spots represent accumulations or individual localizations, or similarly, whether the orange haze represents noise or fewer (but real) fluorescent molecules.

We assume this refers to Figure 2C an ED7 (now ED 4). These figures show registered and averaged STED images. Thus, these are fluorescence intensities, but not individual localizations or molecule counts. The orange haze represents real fluorescence – in essence the amount of fluorescence stemming from the flat basal membrane. To enhance clarity, we have added lookup tables for Figure 2 (see below). We have streamlined the language in this regard.
In principle, one could calculate a rough estimate of the number of fluorescently labeled lipid probes in the final sample. We have previously measured the amount of lipid probes incorporated into cellular membranes as a fraction of the overall lipidome, recently estimated an upper bound for crosslinking yields using native MS of crosslinked lipid-protein conjugates using an LTP-PC lipid complex as a model system. If one assumes a near complete click chemistry labelling, this is sufficient information to calculate e.g. the average number of labelled lipid probes per pit. We would, however, prefer to not include such an exercise in this manuscript, as it would come with huge errors and therefore would likely not be all that helpful in our opinion. We think that this number would be very valuable, but the best way to do it is probably to use single molecule microscopy techniques in combination with known membrane geometries (e.g. the flat membrane/pit system here), but that would be its own project.

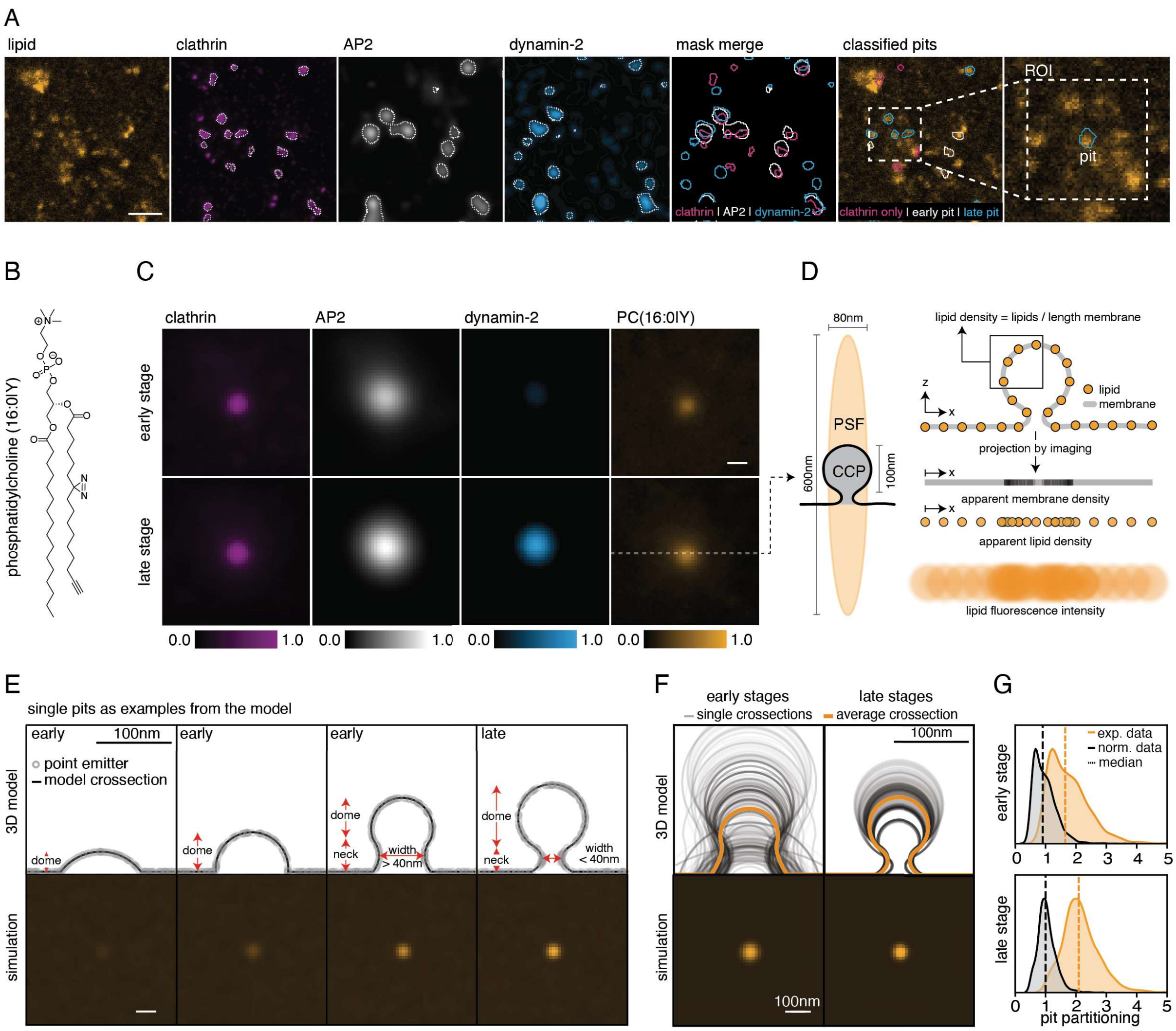

Figure 2: Quantification of lipid partitioning in clathrin-coated pits.

A: Image analysis pipeline. The super-resolved clathrin signal (purple) was used to generate a segmentation mask (white dotted overlay). Pits were classified as clathrin only (purple), early-stage (white), or late-stage pits (blue), depending on their overlap with the AP2 and dynamin-2 masks generated from the respective channels. Apparent lipid partitioning was calculated by measuring the mean lipid intensities of individual pits and division by the mean lipid intensity of the surrounding plasma membrane (ROI). Scale bar: 1μm. B: Chemical structure of PC(16:0/Y). C: Average 4-color fluorescence images for early- and late-stage pits. Fluorescent images of CCPs were centered on the centroid of the clathrin mask before averaging. Scale bar: 100 nm. Averages were acquired from 3 biological replicates. N(early CCPs): 3990, n(late CCPs): 2801. D: A scheme is shown on the left for the dimensions of the experimentally determined point spread function of the microscope in relation to a late-stage CCP. On the right, the process of convolution during imaging is depicted. A pit cross-section with even point emitter distribution is projected into one dimension, resulting in higher apparent membrane densities and lipid densities at the region of the pit invagination. After blurring by the PSF of the microscope, the pit appears brighter compared to the surrounding flat membrane. Lipid density as a readout for lipid partitioning must be determined as the ratio of lipids over surface area. E: Top panels: Cross-sections of modeled CCPs. Bottom panels: Simulated STED for the displayed pit geometries. Scale bars: 100 nm. Representative simulations are shown from a pool of 2000 simulations. F: Average cross-sections (black) of all simulated pits (grey) and averaged simulated images are shown. Scale bar 100 nm. Averages contain 1000 cross-sections for early and late stages each. G: The pit partitioning distributions of early and late-stage pits are shown for PC(16:0IY) before (orange, exp. data) and after (grey, norm. data), normalizing to the median of the model. The respective median of the data is shown as a dotted line.

3. "if their neck diameter measured less than 40 nm" – does this refer to the modeled shapes or actual measurements from EM?

This is referring to the modelled distributions shapes. However, these modelled distributions do reflect the exact parameters determined from the EM dataset, so they should very closely resemble the actual geometries. The geometric parameters from the original EM datasets encompass the entire distribution of pit geometries. We therefore needed a threshold value to distinguish late-stage pits. We considered neck diameter to be the most meaningful geometric parameter for this purpose, as this allowed us to make an informed estimate of the likelihood of dynamin recruitment based on previous reports. The indicated sentence now starts with "Modelled pits were classified…" to make clear what is referred to:

Modelled pits were classified as late-stage if their neck diameter measured less than 40 nm, a curvature that has been reported to facilitate dynamin membrane association *in vitro* [20].

4. "…geometries and resulting apparent partitioning values are not normally distributed" I don't understand the explanation here ("directly reflects the original EM dataset"), nor why a normal distribution would necessarily be the expectation.

5. "A closer inspection of the modeled pit structures" what is the point of this paragraph? Does this have any influence on the interpretation of experiments?

As these two points are related, we provide an answer to both below:
In essence, we were trying to say that the analysis should be focused on late-stage pits. The primary reason for this is the fact that early-stage pits come in a wide variety of shapes and it is not clear whether the EM dataset which contains ~ 200 pits is large enough to fully capture the structural variability of early-stage pits. It is, however, clear from the distribution of shapes in the EM dataset that we cannot assume a normal distribution for early-stage pits. Early-stage pits feature a much broader distribution of predicted surface area values compared to late-stage pits (see ED 5), which means that their the calculated surface area distribution for early stage-pits comes is much more uncertain (see Figure 2F). Since this is the parameter which we use for converting "apparent" into "absolute" partitioning values, it has to be as precise as possible.
Furthermore, it is not clear what the biological meaning of early-stage lipid partitioning is. Late-stage pits presumably closely resemble the lipid composition of clathrin-coated vesicles as they represent the situation immediately prior to the membrane fission event, but this is not expected to be the case for early-stage pits which are temporally more distant from the eventual membrane fission event.
We realize that we did not fully convey the underlying rationale – we have removed the sentence mentioned above and expanded the following paragraph to make our rationale clearer:

A closer inspection of the modeled pit structures showed that late-stage pits are much more consistent in their geometry compared to the early stages (Figure 2F). The early-stage pits contain a mixture of two geometries (domes without necks and domes with necks), but do not capture any transition geometries. A similar effect was not observed for late-stage CCPs, which include pits of similar geometries, indicating a more faithful recapitulation of the late-stage pit structures by the model. Respectively, early-stage pits show a much broader distribution of apparent partitioning values compared to late-stage pits, resulting in a larger expected error for the geometry correction (ED 5). Additionally, lipid partitioning into late-stage pits is much more

meaningful in the context of lipid sorting during CME, as they represent the step immediately before the membrane fission event and thus more closely resemble the lipid composition of the CCVs being released into the cytosol. Therefore, while we here provide measurements for both early- and late-stage pits, we base our conclusions regarding the lipid selectivity of CME primarily on the late-stage CCP lipid partitioning data.

6. “temporally highly resolved uptake kinetics of all lipids into early endosomes” these data look nice and seem quite rigorous, and also nicely align with the partitioning measurements. Why are they relegated to being the only figures in Supplement?

Agreed. We have moved this dataset into the main text as a new main text figure 5, and added the following paragraph:

Uptake kinetics of lipid species into endosomes are similar

Finally, we assessed whether the magnitude of the observed partitioning differences of lipids into CCPs is sufficient to affect bulk endocytic lipid uptake (Figure 5).

To this end, lipids were pulsed to SK-MEL2 wildtype cells for 30 s (ED 9) and chased for defined amounts of time (0 min, 0.5 min, 1.5 min, 3.5 min, 5.5 min or 7.5 min) before crosslinking. Lipid signal in early endosomes was quantified by colocalization with the early endosomal marker EEA1 and normalized to total lipid signal. This quantification provided temporally highly resolved uptake kinetics of all lipids into early endosomes. To extract apparent rate constants and amplitudes of lipid uptake into early endosomes we fitted an empirical mono-exponential model (Figure 5B, C). We found that the rate constants were not significantly different between all tested lipid species with a mean rate constant of 1.09 min^{-1} ± 0.23 min^{-1}. In addition, the amplitude of the lipid signal in the early endosome was similar for all lipids, except GlcCer(Y) and PE(18:1lY) with slightly increased amplitudes (Figure 5B, D). Visual inspection of the lipid signal for GlcCer(Y) and PE(18:1lY) revealed faster depletion of the lipid signal at the plasma membrane, which suggests competing non-vesicular lipid transport processes as previously reported [3]. Thus, it is likely that this difference was caused by overlapping lipid signal from the ER, or by differential uptake of GlcCer(Y) and PE(18:1lY) by other non-CME lipid transport routes to the early endosome within the given time frame.

Together, these data imply that vesicular transport of lipid material from the plasma membrane to the early endosome is largely unspecific, in line with our partitioning analysis.

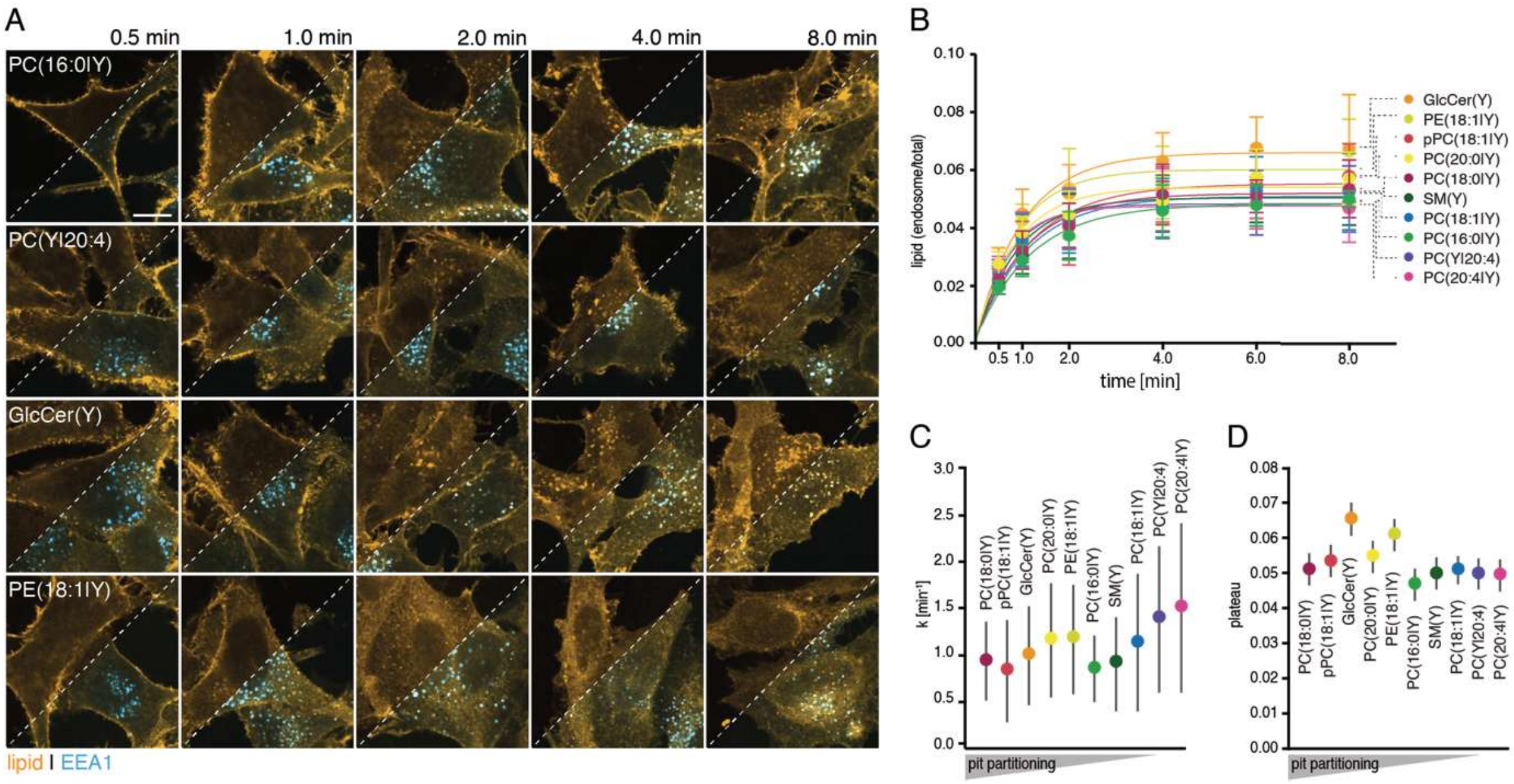

Figure 5: Lipid uptake into early endosomes reveals moderate sorting during endocytosis. A: Exemplary fluorescent images of SK-MEL2 wildtypes cells loaded with lipid for 0.5 min and chased for 0 min, 0.5 min, 1.5 min, 3.5 min, and 7.5 min are shown. The left half of each image shows the lipid channel only (orange), and the right half the overlay with the EEA1 co-stain (blue). Scale bar: 10μm. B: Relative fluorescent lipid signal in early endosomes over total lipid signal was quantified for six different time points and 10 lipids for 3 biological replicates. N(fluorescence image per timepoint per lipid): 36. Mean and standard deviation are shown. Uptake traces were fitted to a one-phase association model. The order of lipids from highest to lowest early endosomal amount at 8 min is shown to the right. C: The rate constants determined from the fit are plotted with the 95% confidence interval error are shown. Rate constants are ordered from highest to lowest pit partitioning. D: The amplitudes of each fit are shown with the 95% confidence interval error indicated. Amplitude values are ordered from highest to lowest pit partitioning.

7. Its pretty confusing that there are both “Extended” and “Supplementary” figures. See rec above to move Supp Figs into main manuscript.

We distributed all data that were in the SI between main text and ED figures, there are no more SI figures in the revised version.

8. The red lines in Fig 4C are weird. I totally don’t understand the one for lipid shape… and this behavior is also inherently conflated with asymmetry (inverted cone fits well if on outer leaflet, not on inner). More minor, why is the red line shifted in rightmost graph? If its just a cartoon, might as well put it right on top of your fit!

We removed the red lines from the data graphs and placed small cartoon that show the expected correlation above the data graphs. Alternatively, we could also completely remove the visual representation of the expected outcomes, but we’d argue that a graphical representation of expectations helps with readability. Regarding the expected correlation for lipid shape and pit partitioning: according to the model, we expect higher enrichment of inverse cone and cone shaped lipids in a curved membrane while cylindrical lipids would be relatively depleted.

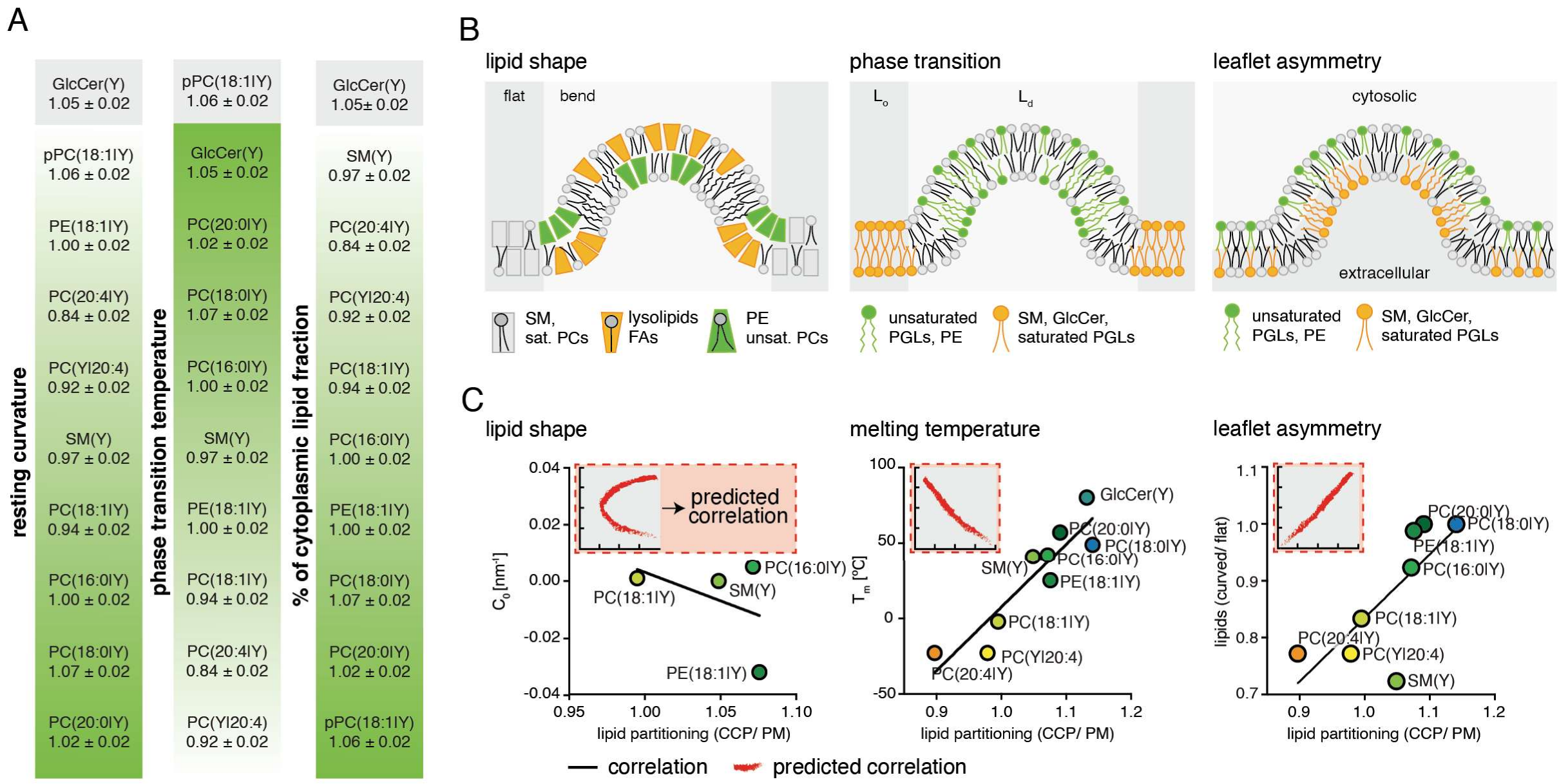

Figure 4: Leaflet asymmetry is sufficient to explain lipid partitioning into clathrin-coated pits. A: Bifunctional lipids were ordered according to the resting curvature, melting temperature, and transbilayer distribution (cytoplasmic/ exoplasmic) of their native lipid counterparts based on reported values from the literature or estimates based on close structural analogs. B: Schemes of possible mechanisms of lipid sorting into clathrin-coated pits are shown. L_d – liquid disordered phase; L_o – liquid-ordered phase. C: The median of absolute lipid densities of late-stage pits is plotted against the resting curvature (C_0), melting temperature (T_m), and the ratio of estimated lipids in a vesicle over a flat membrane of the same surface area determined from their respective native lipid counterparts. The values plotted can be found in the supplement 2 - 4. Only the lipids are plotted for which the corresponding values of C_0, T_m, and lipid asymmetry were reported in the literature. The red line is a sketch and indicates the expected trend of the correlation according to the model.

Reviewer #2 (Remarks to the Author):

The manuscript titled " Quantification of lipid sorting during clathrin-mediated endocytosis" investigates the role of clathrin-mediated endocytosis (CME) in lipid sorting and quantifies the distribution of 10 different lipids in clathrin-coated pits (CCPs) using super-resolution STED microscopy and mathematical modeling. The findings reveal that the differential distribution of lipids in CCPs is primarily driven by lipid asymmetry in the cell membrane. By integrating super-resolution STED microscopy with mathematical modeling, the study provides a novel method for quantifying lipid distribution in cellular membrane compartments. This technological advancement offers an important tool for future cell biology research, with potential applications.

We thank Reviewer #2 for the positive assessment of our work and the constructive suggestions.

Major issues

1. The lipid probes used in the study were chemically modified (e.g., by introducing diazirine and alkyne groups). Might these alterations substantially impact their polarity, solubility, or protein-binding affinity? Could such changes influence their distribution and behavior within the cell membrane? Additionally, could the use of these modified probes disrupt normal cellular processes, such as membrane dynamics or protein function? Are there experimental approaches available to exclude these possible effects?

This is an important point that has also come up in the revision process of our previous lipid imaging papers. We have done extensive control experiments to assess the influence of the diazirine and alkyne modifications on lipid function in cells. The overall outcome is the following:

1. Biophysically, bifunctional lipids most closely resemble native lipid species that carry one additional double bond in the place of the diazirine, which makes sense, as the kink induced by the diazirine unit with its internal angle of 60 degree closely matches that of a double bond.
2. With regard to their metabolism, bifunctional lipids also behave similar to native lipid species that carry one additional double bond in the place of the diazirine
3. Delivery of bifunctional lipids does not change the native lipidome composition
4. Endocytic structures and cargo uptake rates are not affected by delivering bifunctional lipid probes.

We have added two sections to the manuscripts that summarize these results, which now reads as follows:

To quantify the role of CME for sorting lipids at the plasma membrane, we optimized our recently developed lipid imaging workflow [3] for super-resolution STED microscopy. The lipid imaging approach relies on near-native bifunctional lipids, which are modified with a diazirine and alkyne moiety. The diazirine can be photochemically crosslinked to neighboring biomolecules, while the alkyne is used for labelling by click chemistry. These probes closely resemble native lipids with regard to their metabolism and biophysical properties. The combined effect of the modifications is approximately equivalent to the introduction of one additional double bond [3,17,18] (ED 1). Briefly, bifunctional lipids were loaded to the outer leaflet of the plasma membrane and fixed via subsequent UV-light- and chemical crosslinking (Figure 1A).

And:

UV-crosslinking and fixative addition were completed within < 10 seconds, which allowed to capture snapshots of CCPs at different maturation stages [19], under conditions that do not affect major membrane trafficking pathways [17].

We would like to note, though, that all lipids are bioactive molecules and this activity is to a large degree retained in bifunctional lipids, as they so closely resemble their native counterparts (e.g., bifunctional versions of lipid second messengers trigger similar responses compared to their native counterparts, see Höglinger et al. PNAS, 114, 1566-1571, 2017). While we do not see strong disruptions of cellular functions by bifunctional lipids, we do expect that lipid-specific protein expression changes/phosphorylation patterns will occur after delivery as a consequence of the inherent biological activity.

Below, we provide a summary of the control experiments that were carried out to reach the conclusions detailed above. Please note that most of these datasets were included in our previous manuscripts (Iglesias-Artola et al., Nature, 646, 474-482, 2025, Lennartz et al. Nature Cell Biology, 28, 828-837, 2026), with detailed explanations (which we partially re-use here) also given during the peer review of these manuscripts, except for the biophysical characterization of probes newly synthesized for the present manuscript (new ED 1).

Biophysical characterization:

We relied on two different assays to establish the effect of the modifications on lipid behavior ((Iglesias-Artola et al., Nature 646, 474–482 (2025); Supplementary Figure 1, shown below as Reviewer Figure 2), new ED figure 1 in this manuscript showing the results for probes that were not included in the Iglesias-Artola et al. paper). We (i) compared the influence of bifunctional lipids on membrane nanodomain formation with their native analogues lacking diazirine and alkyne moieties. Additionally (ii), Laurdan temperature scans were performed on vesicles incorporating bifunctional lipid probes to assess the influence of the dual modifications on membrane phase behavior. These combined analyses demonstrate that the changes in biophysical properties imposed by the diazirine and alkyne units are minimal and are comparable to, or slightly less pronounced than, the effect of introducing a single additional double bond into the acyl chain; in other words, the bifunctional chain most closely resembles a 16:1 acyl chain. Experimental details on the respective assays are now included in the Supplementary information.

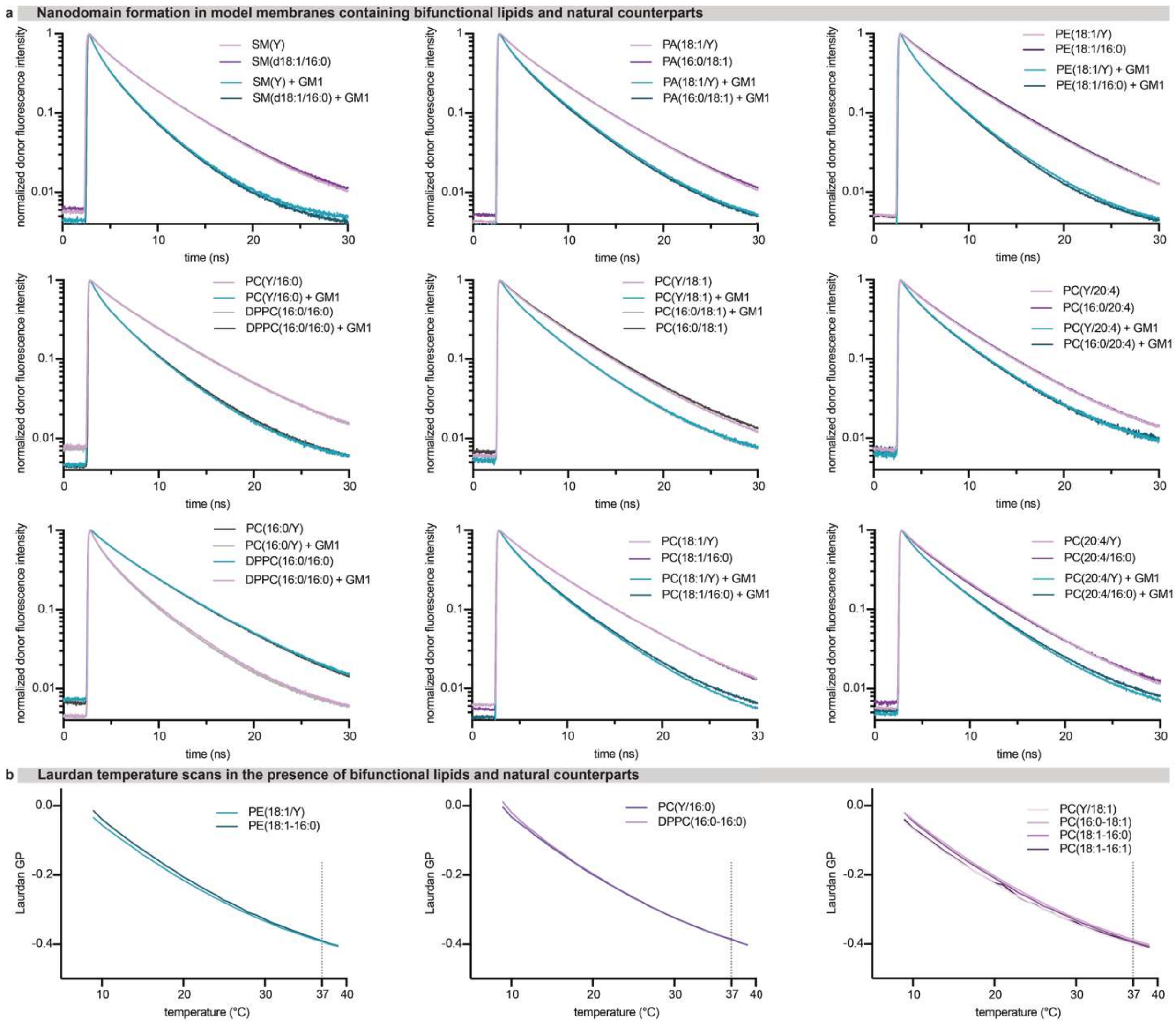

Reviewer Figure 1: Biophysical characterization of bifunctional lipid probes. **a.** Bifunctional lipid probes were characterized for their biophysical properties. Formation of ganglioside nanodomains leading to faster deexcitation of Bodipy-FL-GM1 donors via FRET is unaffected by replacing the closest structurally related native lipid with the bifunctional variant for all probes. **b.** Laurdan temperature scans indicate that the biophysical implications of introducing a bifunctional fatty acid are small.

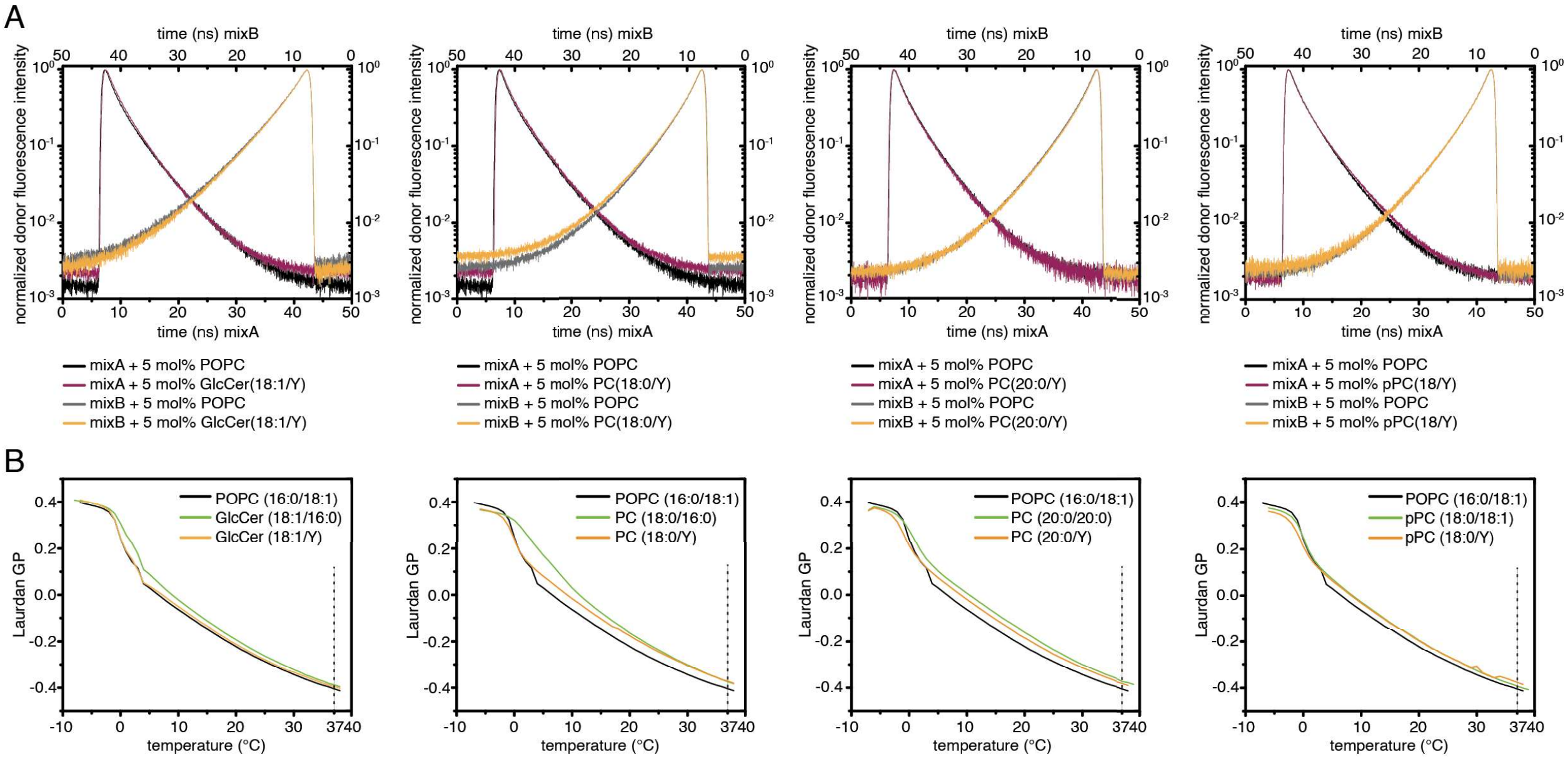

Extended Data 1: Biophysical characterization of bifunctional lipid probes by comparison to native counterparts. A: Formation of ganglioside nanodomains leading to faster deexcitation of Bodipy-FL-GM1 donors via FRET is unaffected by replacing the closest structurally related native lipid with the bifunctional variant for all probes. Lipids tested included GlcCer(18:1/Y), PC(18:0/Y), PC(20:0/Y), and pPC(18/Y). MixA: DOPC/Cholesterol/SM (65/25/10 in mol%) containing lipid nanodomains with the average radius of 9 nm and occupying 45% [14] of the membrane surface, mixB: DOPC/Cholesterol/SM (75/25/10 in mol%) + 4% GM1 with the nanodomains of the radius 99 nm and occupying 50% [15] of the membrane surface. Data was acquired for 1 replicate, n(vesicles per condition): at least 5. **B:** Laurdan temperature scans indicate that biophysical implications of introducing a bifunctional fatty acid are small. Equivalent FRET and Laurdan experiments for the additional lipids used in this study have been previously reported by Iglesias-Artola et al. [3]. Data was acquired for 1 replicate.

Lipid metabolism:

We compared the metabolic processing of bifunctional lipids to that of endogenous lipids (Iglesias-Artola et al., Nature 646, 474–482 (2025); Extended Data Figure 5). Specifically, we compared the metabolic fate of bifunctional PC species carrying an oleic or palmitic acid with a native isotope-labelled species, PC(18:1/16:0[C13]). Bifunctional and isotope-labelled lipids were delivered to the outer leaflet of the plasma membrane by a cyclodextrin pulse. Our results indicate that PC(18:1/16:0[C^{13}]) is metabolized similarly to the bifunctional PC species PC(Y/16:0) and PC(16:0/Y), whereas the species containing oleic acid (PC(Y/18:1) and PC(18:1/Y)) are metabolized at a somewhat faster rate. Importantly, the retention of the original PC(18:1/16:0[C^{13}]) species, expressed as a percentage of all labelled lipids, is comparable to that of the palmitate-containing bifunctional lipids.

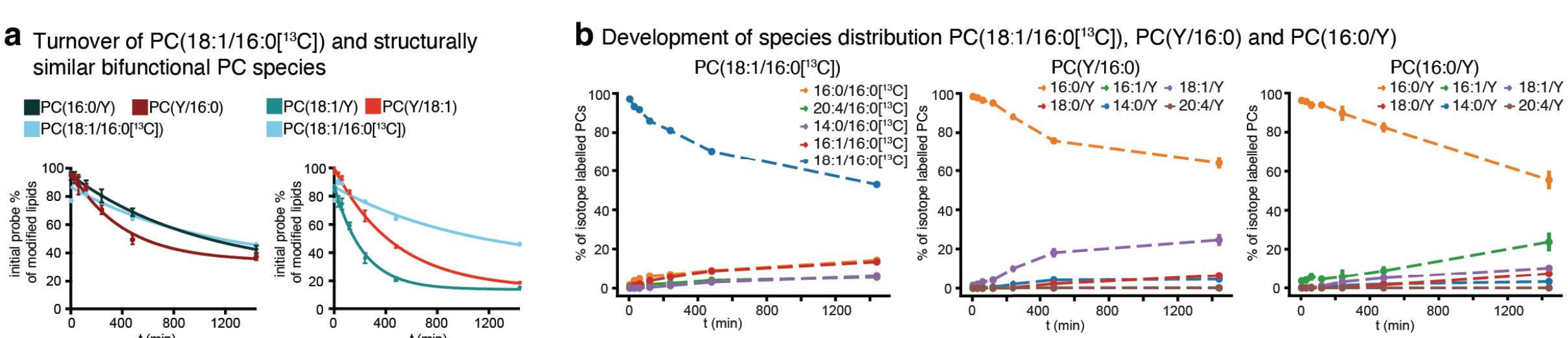

Reviewer Figure 2: Probe Behavior of bifunctional lipids. **a**. Comparison of bifunctional lipid metabolism with the native, isotope labelled, monounsaturated PC species PC(18:1/16:0[^{13}C]). **b**. Development of the PC species distribution of PC(18:1/16:0[^{13}C]) and palmitate-containing bifunctional PC species shows similar persistence of the original species in the labelled lipidome and similar PC product species forming. Mean and 95% CI of 3 biological repeats containing 2 technical replicates each are shown for **a, b**.

Native lipidome composition:

In our recent study (Iglesias-Artola et al., Nature 2025), we analyzed the effects of lipid treatment on the composition of the native lipidome. We did not observe any significant short- or long-term changes in response to lipid loading into the plasma membrane, both at the level of lipid classes and lipid species (Iglesias-Artola et al., Nature 2025, Extended Data Figures 1 and 5, exemplarily summarized in Reviewer Figure 3 below). These data indicate that there is no membrane remodeling at the level of lipid metabolism in response to bifunctional lipid incorporation.

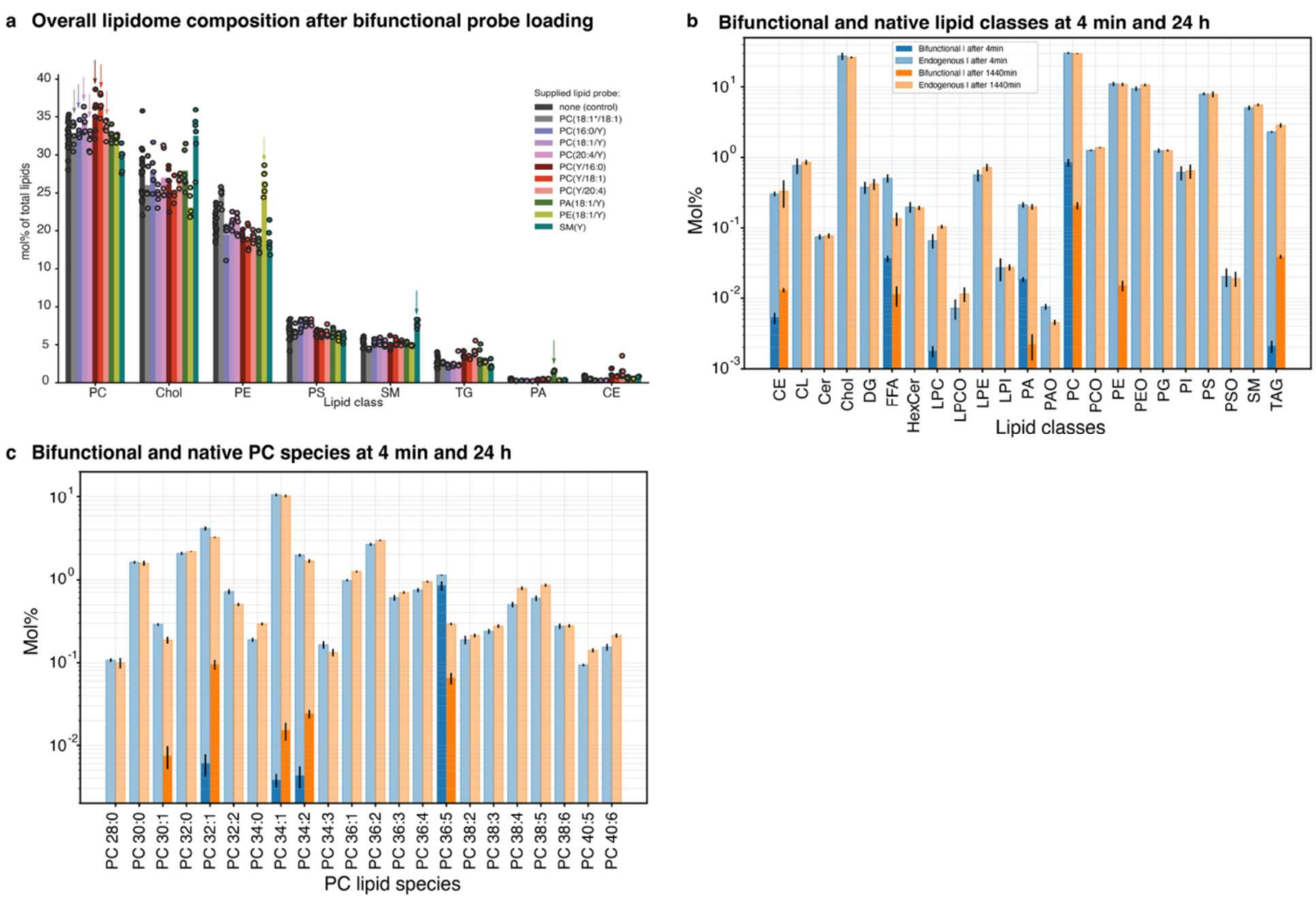

Reviewer Figure 3: The native lipidome is unaffected after bifunctional lipid loading**. a.** Comparison of lipidome composition directly (native and bifunctional lipids combined) after lipid loading bifunctional lipid probes (4 min timepoint) with control lipidome. Arrows indicate supplied lipid type. Bars show the mean of 3 biological repeats containing 2 technical replicates each. (Iglesias Artola et al., 2025, ED figure 1c) **b.** Mol% profile acquired at two time points (see inset for color coding) of 23 lipid classes (light bars), of which 7 classes comprise lipids with bifunctional lipid moieties (dark bars) produced from PC Y/20:4 (Iglesias Artola et al., 2025, ED figure 5b). **c.** PC profile covering 22 species with 5 species containing the bifunctional fatty acid. PCs bearing a bifunctional fatty acid (16:1) are annotated as endogenous lipids having the same number of carbons and double bonds in both FA moieties, albeit having different (+28.0061 Da) masses (Iglesias Artola et al., 2025, ED figure 5c).

Membrane trafficking functions

In our recent story on correlative light and electron microscopy of lipids (Lennartz et al. Nature Cell Biology, 28, 828-837, 2026, ED Figure 2, shown here as Reviewer Figure 4) we assessed the potential effects of lipid loading on the number of endocytic structures and cargo uptake. We performed the following experiment: We loaded U2OS cells with different lipids varying in fatty acid chain length and saturation that differ in their resting curvatures, including the bifunctional SM which is the primary probe used in this study as well as PC species with a wide range of different fatty acid compositions.
To determine whether these probes affect endocytic structures and endocytic rates, we quantified transferrin uptake by endocytosis following lipid loading, as well as the number of clathrin-coated and caveolar pits at the PM. As shown, transferrin uptake was unchanged under all lipid-loading conditions compared with the control (no lipid). Likewise, we did not detect significant differences in the number of clathrin-coated pits. A modest reduction (8 ± 2%) in the number of caveolae associated with the PM was observed upon loading with bifunctional SM. This suggests that there could be a small effect on endocytosis via caveolae, however, the magnitude of this effect is below the variance between cells. We conclude that incorporating bifunctional lipids using alpha-methyl-cyclodextrin mediated incorporation into the plasma membrane does not affect membrane trafficking events at the plasma membrane in a cell biologically relevant fashion.

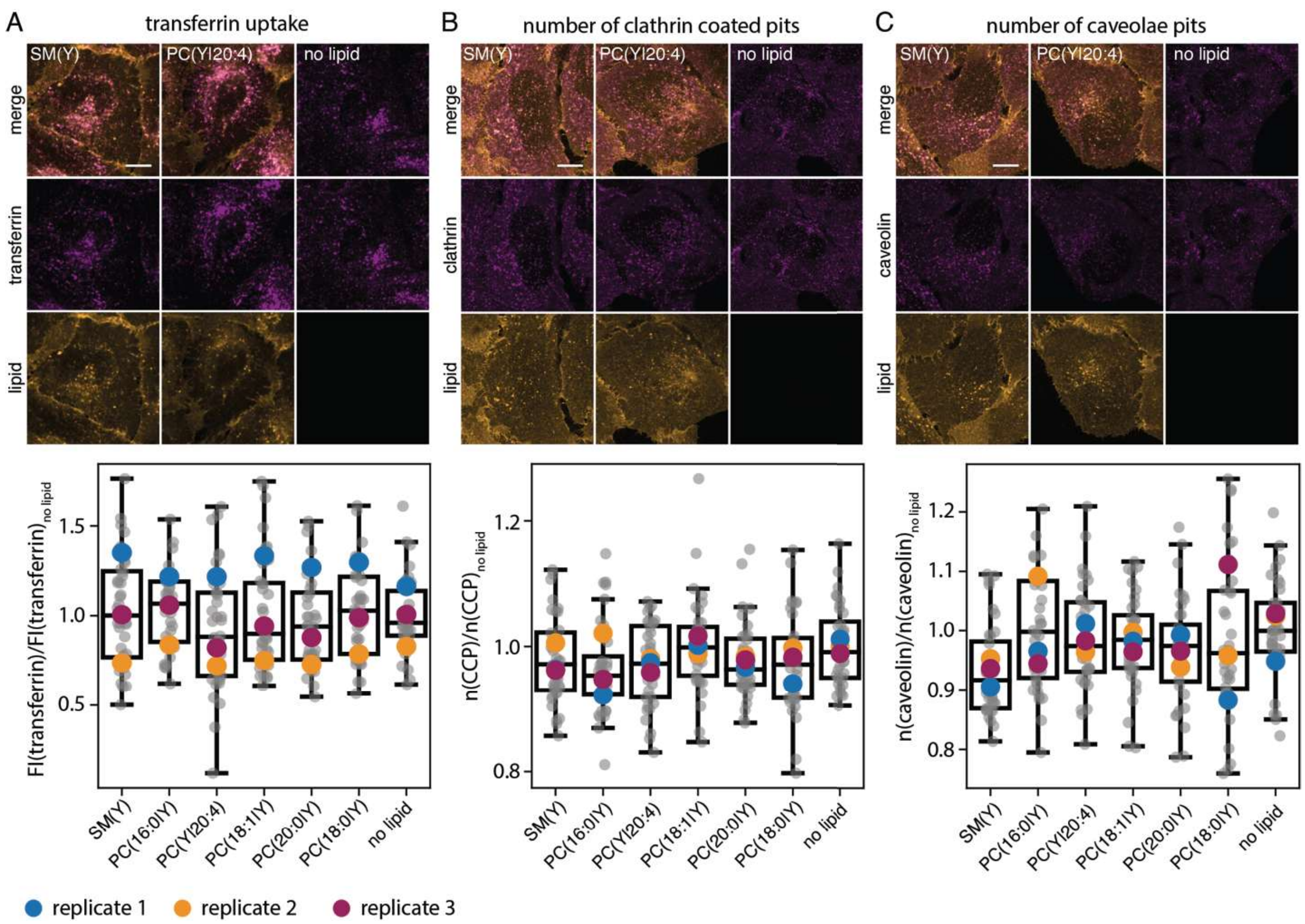

Reviewer Figure 4: Lipid loading does not perturb major endocytic membrane remodeling events. Several bifunctional lipids, including SM(Y) and PCs of different fatty acid composition, were loaded onto the PM of U2OS cells. Transferrin uptake, number of clathrin-coated pits, and caveolae number at the PM were quantified. Three independent replicates are color-coded as indicated. The boxplot indicates the median and interquartile range. The colored dots highlight the mean value of each replicate.

2. Can these probes effectively detect the distribution of lipids on endosomes?

Yes, they can. In fact, our latest paper reports a methodology for visualizing sub-organellar lipid distribution of bifunctional lipid probes using correlative light and electron microscopy imaging specifically on endosomal membrane compartments:

H. Mathilda Lennartz*, Suman Khan#, Kristin Böhlig#, Gunnar Fabig, Yannick Kieswald, Weihua Leng, Falk Elsner, Nadav Scher, Michaela Wilsch-Bräuninger, Ori Avinoam, and André Nadler*; Visualizing sub-organellar lipid distribution using correlative light and electron microscopy. *Nature Cell Biology*, 28, 828-837 (**2026**)

We also quantify lipid-species-specific endosomal content during lipid transport in a time-dependent manner in this manuscript (new main text figure 5, shown below), and in our initial lipid imaging paper:

Juan M. Iglesias-Artola, Kristin Böhlig, Kai Schuhmann, Katelyn C. Cook, H. Mathilda Lennartz, Milena Schuhmacher, Pavel Barahtjan, Cristina Jiménez López, Radek Šachl, Vannuruswamy Garikapati, Karina Pombo-Garcia, Annett Lohmann, Petra Riegerová, Martin Hof, Björn Drobot, Andrej Shevchenko, Alf Honigmann*, and André Nadler*; Quantitative imaging of lipid species-specific transport in living cells. *Nature*, 646, 474-482 (**2025**).

New main text Figure 5:

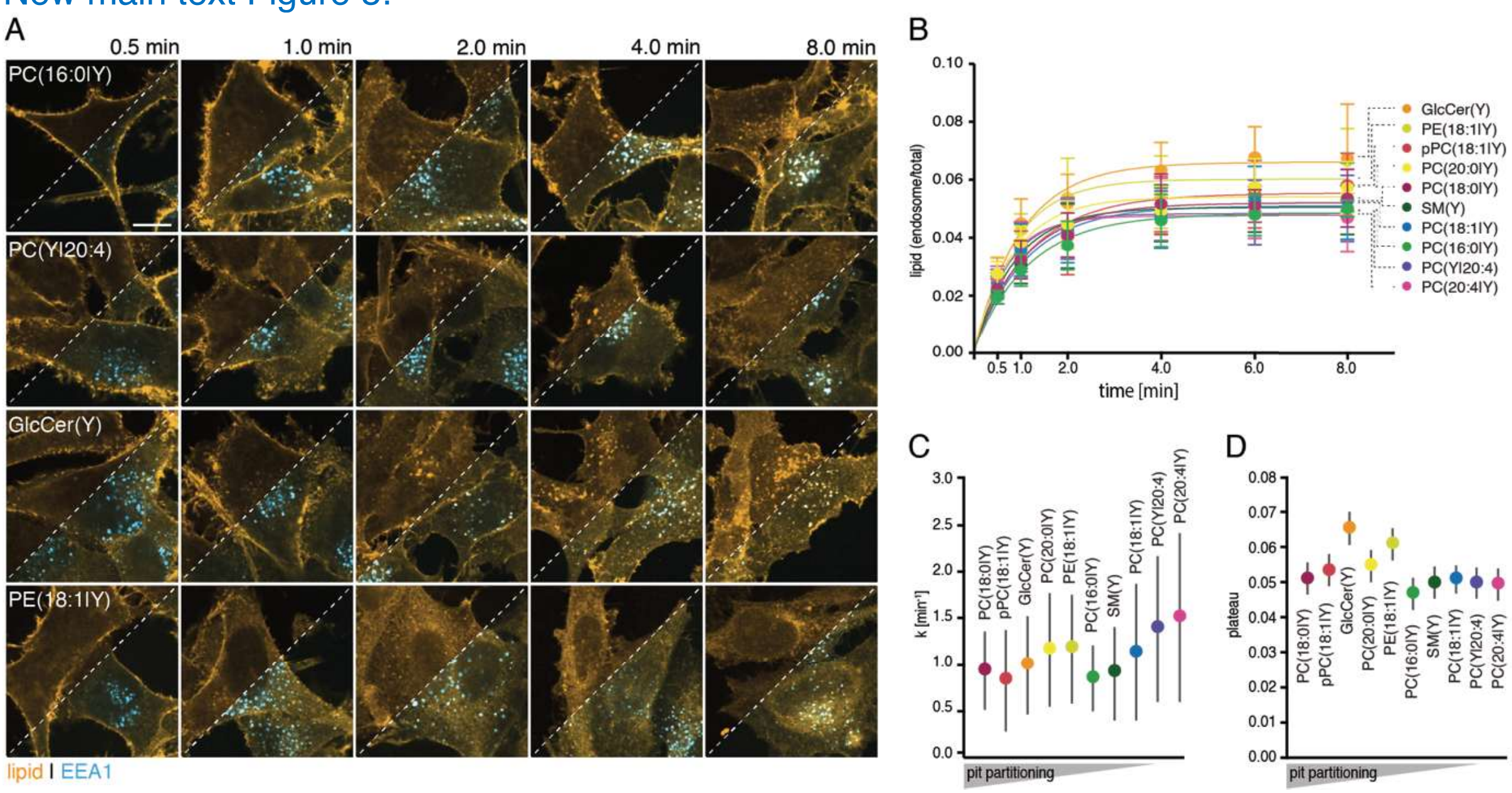

Figure 5: Lipid uptake into early endosomes reveals moderate sorting during endocytosis. A: Exemplary fluorescent images of SK-MEL2 wildtypes cells loaded with lipid for 0.5 min and chased for 0 min, 0.5 min, 1.5 min, 3.5 min, and 7.5 min are shown. The left half of each image shows the lipid channel only (orange), and the right half the overlay with the EEA1 co-stain (blue). Scale bar: 10μm. B: Relative fluorescent lipid signal in early endosomes over total lipid signal was quantified for six different time points and 10 lipids for 3 biological replicates. N(fluorescence image per timepoint per lipid): 36. Mean and standard deviation are shown. Uptake traces were fitted to a one-phase association model. The order of lipids from highest to lowest early endosomal amount at 8 min is shown to the right. C: The rate constants determined from the fit are plotted with the 95% confidence interval error are shown. Rate constants

are ordered from highest to lowest pit partitioning. D: The amplitudes of each fit are shown with the 95% confidence interval error indicated. Amplitude values are ordered from highest to lowest pit partitioning.

3. The study's mathematical model accounts for the intricate geometry and dynamic behavior of CCPs, suggesting that lipid asymmetry in the cell membrane is the key driver of lipid sorting in CCPs. Is it possible to further confirm this hypothesis with additional experimental evidence?

This is an important point that was also brought up by reviewer #1 and reviewer #3. We have expanded the discussion of this aspect, also in the light of new experiments that we have conducted. Briefly, we tried to perturb lipid asymmetry and measure the consequences for lipid partitioning into clathrin coated pits. To avoid long-term cellular adaptations upon genetic perturbations (for instance, TMEM30A KD causes broad remodeling of the cellular lipidome and proteome and results in a major change of plasma membrane biophysics), we induced plasma membrane scrambling using ionomycin generated calcium influx in SK-MEL cells (ED8A).Calcium activates a subset of endogenous plasma membrane resident scramblases, thus initiating scrambling without the need of genetic perturbations, and at fast time scales (seconds to minutes). Scrambling was monitored by PS exposure (ED 8B). Calcium-induced lipid scrambling lead to a mis-localization of clathrin, fast dissociation of dynamin-2 GFP, and an almost instant block of transferrin uptake (ED 8C - E). This data suggests that clathrin-coated pits disassemble upon plasma membrane scrambling, with a kinetic that appeared to be faster than the rate of PS externalization, indicating that there is no time-window during which a scrambled plasma membrane lipid composition and regular CCP organization can co-exist (ED8C-E). To understand whether CCP disassembly is indeed caused by plasma membrane scrambling and not the sudden influx of calcium, we induced calcium influx in HEK293 cells, that have been reported for their low endogenous scramblase activity [1,2]. In HEK293, while we do monitor calcium influx, we could not observe PS exposure to the extracellular leaflet, indicative of the lack of PM scrambling. In ionomycin - calcium treated HEK293 cells, clathrin localization appears unaltered compared to the untreated control. These data are included in the manuscript in new ED Figure 8. It appears – at least in our hands – that CME and lipid asymmetry are too closely linked to be functionally separated.

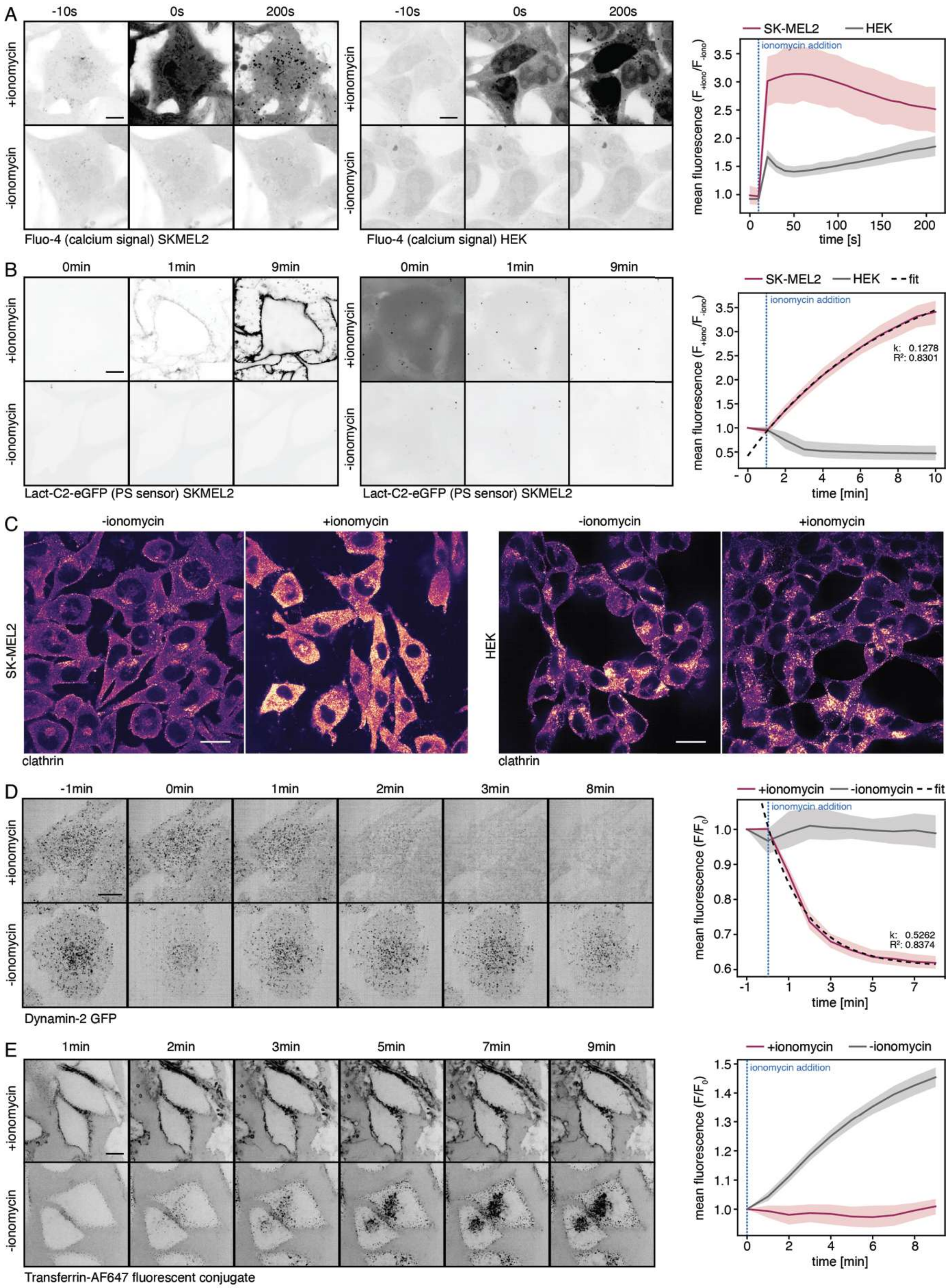

Extended Data 8: Clathrin-coated pits disassemble upon plasma membrane scrambling. SK-MEL2 or HEK293 cells were treated with 5 μM of the ionophore ionomycin in buffer containing 2.5 mM Ca^{2+}. **A**: Relative cytosolic calcium was monitored during life cell imaging for 200 s at 10 s intervals using the Fluo-4 Calcium Imaging Kit (Invitrogen),

signal shown in grey. The average Fluo-4 signal of SK-MEL2 (purple) or HEK293 (grey) cells is plotted against time. The signal is normalized to the mean signal of the control (no ionomycin) across time. The error of the normalized average fluorescent signal is shaded and given as the confidence interval (CI) of 95%. Three independent biological samples were analyzed for each condition, n(total fields of view): 18. **B**: Phosphatidylserine (PS) exposure to the extracellular leaflet of the plasma membrane is monitored as a proxy for plasma membrane scrambling. PS exposure is measured using the PS sensor Lact-C2-eGFP [3] over 10 min at 1 min intervals of SK-MEL2 (purple) or HEK293 (grey) cells. The GFP signal is normalized to the mean signal of the control (no ionomycin) across time. The error of the normalized average fluorescent signal is shaded and given as CI: 95%. Three independent samples were analyzed for each condition, n(total fields of view): 18. The SKMEL trace was fitted (black dotted line) using a simple exponential one phase association model. The rate constant k and R^2 are indicated. **C**: SK-MEL2 (left panel) or HEK293 (right panel) cells were treated with ionomycin for 5 min in buffer containing 2.5mM Ca^{2+} and fixed. Clathrin localization was assessed using immunostaining. Representative images of 3 independent biological replicates are shown, that yielded similar results. **D**: Dynamin-2 of a SK-MEL2 dynamin-2-GFP cell line localization was monitored over 10 min at 1 min intervals of cells treated with (purple) or without (grey) ionomycin at the basal plasma membrane. The mean GFP signal is normalized to the mean signal at time -1 min (relative to ionomycin addition). The error of the normalized average fluorescent signal is shaded and given as CI: 95%. Three independent samples were analyzed for each condition, n(total fields of view per condition): 18. The SKMEL trace was fitted (black dotted line) using a simple exponential one phase dissociation model. The rate constant k and R^2 are indicated. **E**: The uptake of a fluorescently tagged transferrin conjugate was monitored over 10 min at 1 min intervals of cells treated with (purple) or without (grey) ionomycin. The mean fluorescent signal is normalized to the mean signal at time -1 min (relative to ionomycin addition). The error of the normalized average fluorescent signal is shaded and given as CI: 95%. Three independent samples were analyzed for each condition, n(total fields of view): 30. Scale bars: 10μm. Scale bars of ROIs: 1μm.

The corresponding new section in the results now reads:

Despite the experimental challenges associated with perturbing lipid transbilayer composition in living cells, we attempted to investigate the proposed connection between lipid asymmetry and lipid partitioning into CCPs. Since genetic perturbations of the flippase machinery that establishes lipid asymmetry (TMEM30A) result in complex remodeling of the lipidome, proteome and metabolism that go far beyond a simple loss of lipid asymmetry[4,5], we opted for an acute perturbation. To this end, we induced lipid scrambling mediated by the Ca^{2+}-dependent scramblase TMEM16F by treating both SK-MEL2 cells, which express TMEM16F and HEK293 cells, which have low endogenous scramblase activity [1,2] with ionomycin in a calcium containing buffer. Both cell types responded with robust calcium influx (ED 8A), but phosphatidylserine (PS) externalization was observed only in SK-MEL2 cells (ED 8B). Thus, by comparing both cell lines we wanted to separate the effects of elevated Ca^{2+} levels from the scrambling of plasma membrane lipids. However, we found that upon ionomycin treatment clathrin was rapidly released from the plasma membrane in SK-MEL2 but not in HEK293 cells (ED 8C). Similarly, the plasma membrane bound pool of Dynamin-2-GFP was fully released 3 min after ionomycin addition. Indeed, late-stage CCPs disassembled with a ~4 times faster kinetic (rate constant $k_{(dynamin\ disassembly)} \pm$ CI95: 0.5262 ± 0.0691) compared to PS externalization ($k_{(PS\ assembly)} \pm$ CI95: 0.1278 ± 0.0564) (ED 8D). In line with this data, we found that ionomycin also fully blocks transferrin uptake in SK-MEL-2 cells (ED 8E). Thus, experimentally it was not possible to monitor lipid partitioning into CCPs after plasma membrane scrambling.
Together, our data indicates that the asymmetric lipid composition of the plasma membrane is a prerequisite for CME and that lipid asymmetry and CME are too closely linked to be functionally separated. This finding is in line with previous studies [6] and the reported role of lipids, such as $PI(4,5,)P_2$ and PS, to facilitate the binding of multiple proteins of the CME machinery [6,7].

Thus, despite our best efforts, we were not able to disentangle the effect of lipid steady-state asymmetry from a putative effect purely caused by biophysical properties of lipids by experimental means. It may very well be that this is a fundamental problem since lipid asymmetry requires the mobilization of lipids from the bulk bilayer by lipid translocases – and the transition states of the

corresponding reaction coordinates will depend on the biophysical properties of the substrate lipid species.
With regard to the conclusion of this manuscript, we have adapted the discussion to account for this uncertainty. Nevertheless, we do think that this is a case were Ockham's razor applies. The respective sections now read as follows:

In addition, we found a strong correlation between melting temperature and CCP partitioning, with high melting temperature lipids partitioning more strongly into CCPs. This is in contrast to the expected depletion of high melting temperature lipids into curved membranes, based on previous reports [8–11] and the biophysical properties of a curved membrane [12,13]. Thus, it is likely that the observed lipid partitioning trends are not explained by classical L_o/L_d phase separation, which would result in the opposite partitioning behavior. In this context, it is an intriguing observation that melting temperature and lipid asymmetry are positively correlated, at least for the glycerophospholipids (ED 10). We have previously observed similar trends for the rates of non-vesicular lipid transport and metabolism for different PC species [3]. The initial step in all three processes is the mobilization/extraction of a lipid from the bulk bilayer by a protein (lipid translocases, lipid transfer proteins, lipid metabolizing enzymes). It is a compelling speculation that lipid mobilization, which should depend heavily on lipid biophysical properties, could be the rate limiting step in all cases. If this were the case, lipid biophysical properties would be a key determinant of trans-bilayer, intra- and inter-organelle lipid distribution.

4. Are there variations in lipid distribution within the cell membranes of different cell types, such as phagocytic versus non-phagocytic cells? If such differences exist, could they impact the phagocytic capabilities of these cells?

To the best of our knowledge, this has not been comprehensively addressed in a comparative study that combines functional analysis of phagocytosis with quantitative, organelle-specific lipidomics measurements. We can only speculate that the plasma membrane lipidome is cell-type specific, see for instance very interesting recent work by the D'Angelo lab who find significantly different lipidomes across cell types and have formulated the lipotype hypothesis, which states that the cell-type specific lipidome is part of the cellular identity and closely linked to its function [21,22]. Thus, it would not be surprising if the effect that #reviewer 2 predicts does exist. However, actually testing for that using our methodology would be a completely new project. We hope that reviewer #2 can agree with us that this would be out of scope of the current manuscript.

Reviewer #3 (Remarks to the Author):
If and how the lipids are sorted by clarthrin-mediated endocytosis (CME) is an important biological question. In this paper, Lennartz et al. used super-resolution STED imaging of bifunctional lipid probes, in parallel with mathematical modeling, to compare the partitioning of 10 structurally diverse lipid species at clathrin-coated pits. Their quantitative analysis observes moderate but significant differences of pit partitioning among the 10 lipid species. By correlation analysis with known biophysical properties of lipids, the authors concluded that the moderate differences in pit partitioning are due to leaflet asymmetry of various lipids. Before this study, it was not possible to directly compare lipid partitioning, and pairing the experiments with geometric modeling allowed the authors to extract a weak biological signal. This work provided important direct quantitative evidence for the differential enrichment of several lipid groups at clarthrin-coated pits. Overall, the data presentation is clear and information-rich.

We thank reviewer #3 for the positive overall assessment and a number of suggestions that upon implementation have made our manuscript significantly stronger.

However, two important lipid groups on plasma membrane, phosphatidylserine and phosphatidylinositol, which are implicated in playing key roles during CME, are not examined and are not considered for the mechanism they proposed.

Reviewer #3 is correct – PIP2 and PS do play a functional role in CME. In part (besides, of course not having the corresponding probes available), this is the reason why we did not use them in this study. Delivering PIP2 and PS to the plasma membrane would very likely have functional consequences for CME – and the interpretation of the resulting partitioning data would likely also have to account for changes in CME rate and would not be directly comparable to lipid species that are not directly implicated in the core mechanism of CME. Disentangling these effects would be its own study that would have to start from developing new probe syntheses, and we hope that reviewer #3 can agree with us that this is out of scope for the present manuscript.

We do, however, acknowledge that this is an important point and have included paragraph in the discussion where we discuss the possibility of alternate mechanisms for lipids that are functionally involved in CME, which now reads as follows:

We propose that the main function of CME is to selectively transport proteins, while the associated lipid transport is largely unspecific. On the other hand, specific lipid transport is mediated by non-vesicular mechanisms. We note that our findings here do not preclude sorting of individual lipids during clathrin-independent endocytic processes, with caveolae-mediated endocytosis proposed as one possible lipid sorting mechanism [23], but these mechanisms are likely minor components in the total transport volume of lipids between the plasma membrane and other organelle membranes. We anticipate that our methodology for quantifying lipid partitioning into cellular membrane compartments of complex geometry will help to understand membrane compartmentalization, in particular with regard to lipid transport through the endomembrane system.

The conclusion that inner leaflet residence predicts lipid partitioning was drawn from an oversimplified analysis. In particular, this analysis assumes that all lipid species are primarily partitioned by the same mutually exclusive partitioning mechanism. The differential partitioning data is supported and interesting, but the underlying mechanism is not sufficiently explored.

We actually do not make any assumptions within the framework of our analysis. We estimate which partitioning coefficients are expected from purely due to membrane geometry – which in turn is based on experimental data. We were initially surprised by the small differences and then considered multiple possibilities for explaining the results – including but not limited to lipid asymmetry. Partitioning based on lo/ld phase separation as well as sorting by intrinsic curvature were also considered.

Major comments:

• The reference papers validating the method in the first half of the work are posted on bioRxiv, but a peer-reviewed version is not publicly available.

This has now changed. The two main stories are now published, and can be found here:

Juan M. Iglesias-Artola, Kristin Böhlig, Kai Schuhmann, Katelyn C. Cook, H. Mathilda Lennartz, Milena Schuhmacher, Pavel Barahtjan, Cristina Jiménez López, Radek Šachl, Vannuruswamy Garikapati, Karina Pombo-Garcia, Annett Lohmann, Petra Riegerová, Martin Hof, Björn Drobot, Andrej Shevchenko, Alf Honigmann*, and André Nadler*; Quantitative imaging of lipid species-specific transport in living cells. *Nature*, 646, 474-482 (**2025**).

H. Mathilda Lennartz*, Suman Khan#, Kristin Böhlig#, Gunnar Fabig, Yannick Kieswald, Weihua Leng, Falk Elsner, Nadav Scher, Michaela Wilsch-Bräuninger, Ori Avinoam, and André Nadler*; Visualizing sub-organellar lipid distribution using correlative light and electron microscopy. *Nature Cell Biology*, 28, 828-837 (**2026**)

The revised, peer-reviewed versions of these manuscripts also include additional validations of lipid imaging using bifunctional lipid probes, including a comparison of metabolic turnover with a stable-isotope labelled lipid as well as extensive biophysical characterization. The combination of all these datasets indicate that bifunctional lipids most closely resemble lipids that feature one additional double bond in the place of the diazirine group. For all probes that are newly reported here, we also now provide biophysical characterizations, see new ED Figure 1, show below:

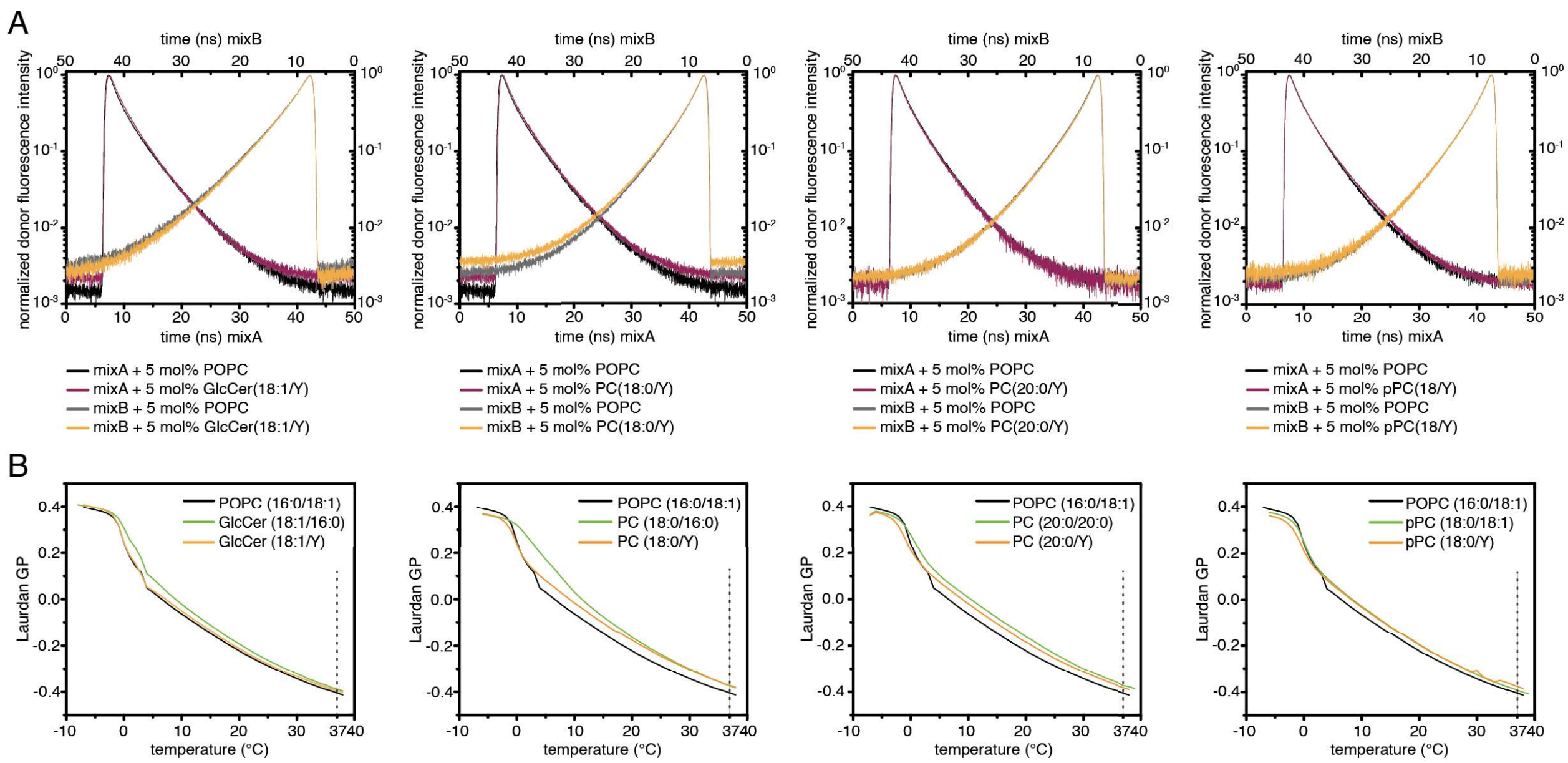

Extended Data1: Biophysical characterization of bifunctional lipid probes by comparison to native counterparts. A: Formation of ganglioside nanodomains leading to faster deexcitation of Bodipy-FL-GM_1 donors via FRET is unaffected by replacing the closest structurally related native lipid with the bifunctional variant for all probes. Lipids tested included GlcCer(18:1/Y), PC(18:0/Y), PC(20:0/Y), and pPC(18/Y). MixA: DOPC/Cholesterol/SM (65/25/10 in mol%) containing lipid nanodomains with the average radius of 9 nm and occupying 45% [14] of the membrane surface, mixB: DOPC/Cholesterol/SM (75/25/10 in mol%) + 4% GM1 with the nanodomains of the radius 99 nm and occupying 50% [15] of the membrane surface. Data was acquired for 1 replicate, n(vesicles per condition): at least 5. **B:** Laurdan temperature scans indicate that biophysical implications of introducing a bifunctional fatty acid are small. Equivalent FRET and Laurdan experiments for the additional lipids used in this study have been previously reported by Iglesias-Artola et al. [3]. Data was acquired for 1 replicate.

We have furthermore published a detailed experimental protocol, to make sure that all interested researchers can replicate our methodology and to explain applicability and limitations:

Martin Buitrago-Arango, Chia-Yi Chou, André Nadler*; Lipid imaging using bifunctional probes, *Methods in Enzymology*, 727, 17-35, (**2026**).

We have included a brief section in present manuscript summarizing the extensive control experiments and method validations done in the meantime, which now reads:

The lipid imaging approach relies on near-native bifunctional lipids, which are modified with a diazirine and alkyne moiety. The diazirine can be photochemically crosslinked to neighboring biomolecules, while the alkyne is used for labelling by click chemistry. These probes closely resemble native lipids with regard to their metabolism and biophysical properties. The combined effect of the modifications is approximately equivalent to the introduction of one additional double bond [3,17,18] (ED 1).

• The abstract states there is “time resolved data” but only late-stage CME sites are analyzed. Figures depicting time evolution of CME, or the modeling of the signal for early-stage CME sites set the reader up to expect time-resolved data.

We disagree with the notion that we are setting up the reader to expect time-resolved data. We did not state that there is “time resolved data” in the abstract. The only time in the entire manuscript where a related phrase is used is during the description of the experimental adaptation of the lipid imaging methodology in the introduction: “We adapted our methodology for super-resolution stimulated emission depletion (STED) microscopy and captured temporally resolved CCP maturation for a library of 10 different lipid species to investigate lipid enrichment in early and late-stage CCPs.” This is simply a description of the experiments that were carried out and our intent to analyse lipid partitioning into early- and late state pits.

Importantly, we did acquire the full datasets for late stage-pits, early stage-pits, and clathrin-only structures and these datasets are included in the originally submitted manuscript version, specifically in Extended Data Figures 6 and 9 (now ED 4 and 5). We then proceeded to model early-stage and late-stage CCPs and simulated the corresponding expected intensity distributions. The outcome of these simulations informed the choice to not go ahead with the calculation of absolute partitioning values for early-stage pits – out of an abundance of scientific caution, but not with an intent to mislead.

The primary reason for our choice not to calculate “absolute” partitioning values is that early-stage pits come in a wide variety of shapes and it is not clear whether the EM dataset which contains ~ 200 pits and informs the modelling is large enough to fully capture the structural variability of early-stage pits. It is, however, clear from the distribution of shapes in the EM dataset that we cannot assume a normal distribution for early-stage pits. This implies that the calculated surface area distributions for early stage-pits comes are much wider and heterogenous compared to the distributions of late-stage pits where the underlying structures are much more uniform. Since modelled surface area is the parameter which we use for converting “apparent” into “absolute” partitioning values, it has to be as precise as possible.

We therefore argue that both the experimental results and the modelling outcomes for early-stage pits provide critical context for our findings and should remain part of the manuscript. To avoid any confusion, we have rephrased the introductory sentence quoted above, which now reads:

“We adapted our methodology for super-resolution stimulated emission depletion (STED) microscopy and captured lipid intensities in CCPs of different maturation stages for a library of 10 different lipid species to investigate lipid enrichment in early and late-stage CCPs.”

We acknowledge that we should have done a better job to explain why we made the choice to base our analysis primarily on late-stage pits (as reviewer #1 also pointed out, the corresponding paragraph was not particularly clearly written) and we have adapted the text as follows to make our reasoning clearer:

A closer inspection of the modelled pit structures showed that late-stage pits are much more consistent in their geometry compared to the early stages (Figure 2F). The early-stage pits contain a mixture of two geometries (domes without necks and domes with necks), but do not capture any transition geometries. A similar effect was not observed for late-stage CCPs, which include pits of similar geometries, indicating a more faithful recapitulation of the late-stage pit structures by the model. Respectively, early-stage pits show a much broader distribution of apparent partitioning values compared to late-stage pits, resulting in a larger expected error for the geometry correction (ED 5). Additionally, lipid partitioning into late-stage pits is much more meaningful in the context of lipid sorting during CME, as they represent the step immediately

before the membrane fission event and thus more closely resemble the lipid composition of the CCVs being released into the cytosol. Therefore, while we here provide measurements for both early- and late-stage pits, we base our conclusions regarding the lipid selectivity of CME primarily on the late-stage CCP lipid partitioning data.

• Figure 4 legend title: "Sufficient to explain" is misleading, while the authors only demonstrate "consistent with a model of" or a correlation.

We changed the figure title as requested.

• The authors should apply geometric modeling to predict expected lipid enrichment for inner- and outer-leaflet resident lipids. Is the relative partitioning of the same order of magnitude as the differences in surface area the authors claim to explain the partitioning?

Indeed, we did exactly this in the originally submitted version. The values in Figure 4 are derived from estimate asymmetry values and pit geometries obtained from the literature, as suggested by reviewer #3. The values and the respective descriptions of the calculations can be found in Supplementary Data 2 and 4. The effect sizes are very similar – with a maximal difference in partitioning of 27 % between the most different lipids, which compares very well to the corresponding 23 % difference we find experimentally. Thus, the effect we find experimentally is of the same magnitude as one would expect from our proposed model. Of note, we compare values reported in the literature for native lipids with our bifunctional lipid probes. The actual values of the bifunctional lipid probes are most likely slightly and consistently shifted, which could explain this deviation. We highlight this notion now in the main text:

These values correspond to the (saturated) native counterparts of the lipid probes used here. The actual values of the bifunctional probes are likely shifted to slightly lower melting temperatures, because the diazirine group resembles one additional double bond (ED 1) [3].

• The conclusion of the second half of the paper, that inner/outer leaflet residence drives lipid partitioning, would be stronger if the authors introduced a biological perturbation that flips the lipid residence, and tests if it changes the partitioning.

We agree with reviewer #3 and have expanded the discussion of this aspect, also in the light of new experiments that we have conducted. Briefly, we tried to perturb lipid asymmetry and measure the consequences for lipid partitioning into clathrin coated pits. To avoid long-term cellular adaptations upon genetic perturbations (for instance, TMEM30A KD causes broad remodeling of the cellular lipidome and proteome and results in a major change of plasma membrane biophysics), we induced plasma membrane scrambling using ionomycin generated calcium influx in SK-MEL cells (ED8A). Calcium activates a subset of endogenous plasma membrane resident scramblases (primarily TMEM16F), thus initiating scrambling without the need of genetic perturbations, and at fast time scales (seconds to minutes). Scrambling was monitored by PS exposure (ED 8B). Calcium-induced lipid scrambling lead to a mis-localization of clathrin, fast dissociation of dynamin-2 GFP, and an almost instant block of transferrin uptake (ED 8C - E). This data suggests that clathrin-coated pits disassemble upon plasma membrane scrambling, with a kinetic that appeared to be faster than the rate of PS externalization, indicating that there is no time-window during which a scrambled plasma membrane lipid composition and regular CCP organization can co-exist (ED8C-E). To understand whether CCP disassembly is indeed caused by plasma membrane scrambling and not the sudden influx of calcium, we induced calcium influx in HEK293 cells, that have been reported for their low endogenous scramblase

activity [1,2]. In HEK293, while we do monitor calcium influx, we could not observe PS exposure to the extracellular leaflet, indicative of the lack of PM scrambling. In ionomycin - calcium treated HEK293 cells, clathrin localization appears unaltered compared to the untreated control. These data are included in the manuscript in new ED Figure 8. It appears – at least in our hands – that CME and lipid asymmetry are too closely linked to be functionally separated.

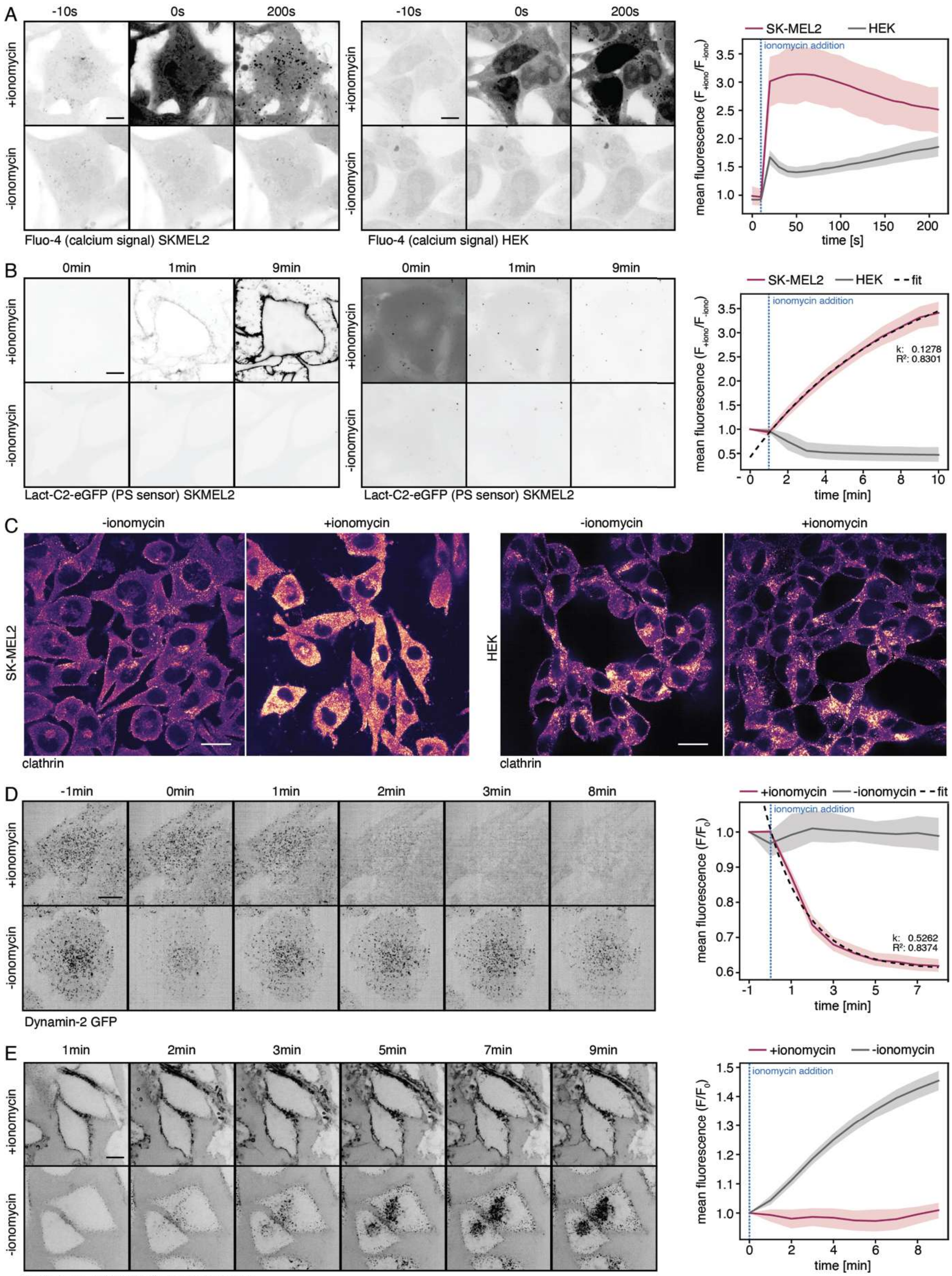

Extended Data 8: Clathrin-coated pits disassemble upon plasma membrane scrambling. SK-MEL2 or HEK293 cells were treated with 5 μM of the ionophore ionomycin in buffer containing 2.5 mM Ca^{2+}. **A**: Relative cytosolic calcium was monitored during life cell imaging for 200 s at 10 s intervals using the Fluo-4 Calcium Imaging Kit (Invitrogen),

signal shown in grey. The average Fluo-4 signal of SK-MEL2 (purple) or HEK293 (grey) cells is plotted against time. The signal is normalized to the mean signal of the control (no ionomycin) across time. The error of the normalized average fluorescent signal is shaded and given as the confidence interval (CI) of 95%. Three independent biological samples were analyzed for each condition, n(total fields of view): 18. **B**: Phosphatidylserine (PS) exposure to the extracellular leaflet of the plasma membrane is monitored as a proxy for plasma membrane scrambling. PS exposure is measured using the PS sensor Lact-C2-eGFP [3] over 10 min at 1 min intervals of SK-MEL2 (purple) or HEK293 (grey) cells. The GFP signal is normalized to the mean signal of the control (no ionomycin) across time. The error of the normalized average fluorescent signal is shaded and given as CI: 95%. Three independent samples were analyzed for each condition, n(total fields of view): 18. The SKMEL trace was fitted (black dotted line) using a simple exponential one phase association model. The rate constant k and R^2 are indicated. **C**: SK-MEL2 (left panel) or HEK293 (right panel) cells were treated with ionomycin for 5 min in buffer containing 2.5mM Ca^{2+} and fixed. Clathrin localization was assessed using immunostaining. Representative images of 3 independent biological replicates are shown, that yielded similar results. **D**: Dynamin-2 of a SK-MEL2 dynamin-2-GFP cell line localization was monitored over 10 min at 1 min intervals of cells treated with (purple) or without (grey) ionomycin at the basal plasma membrane. The mean GFP signal is normalized to the mean signal at time -1 min (relative to ionomycin addition). The error of the normalized average fluorescent signal is shaded and given as CI: 95%. Three independent samples were analyzed for each condition, n(total fields of view per condition): 18. The SKMEL trace was fitted (black dotted line) using a simple exponential one phase dissociation model. The rate constant k and R^2 are indicated. **E**: The uptake of a fluorescently tagged transferrin conjugate was monitored over 10 min at 1 min intervals of cells treated with (purple) or without (grey) ionomycin. The mean fluorescent signal is normalized to the mean signal at time -1 min (relative to ionomycin addition). The error of the normalized average fluorescent signal is shaded and given as CI: 95%. Three independent samples were analyzed for each condition, n(total fields of view): 30. Scale bars: 10μm. Scale bars of ROIs: 1μm.

The corresponding new section in the results now reads:

Lipid asymmetry is required for clathrin-mediated endocytosis

Despite the experimental challenges associated with perturbing lipid transbilayer composition in living cells, we attempted to investigate the proposed connection between lipid asymmetry and lipid partitioning into CCPs. Since genetic perturbations of the flippase machinery that establishes lipid asymmetry (TMEM30A) result in complex remodeling of the lipidome, proteome and metabolism that go far beyond a simple loss of lipid asymmetry[4,5], we opted for an acute perturbation. To this end, we induced lipid scrambling mediated by the Ca^{2+}-dependent scramblase TMEM16F by treating both SK-MEL2 cells, which express TMEM16F and HEK293 cells, which have low endogenous scramblase activity [1,2] with ionomycin in a calcium containing buffer. Both cell types responded with robust calcium influx (ED 8A), but phosphatidylserine (PS) externalization was observed only in SK-MEL2 cells (ED 8B). Thus, by comparing both cell lines we wanted to separate the effects of elevated Ca^{2+} levels from the scrambling of plasma membrane lipids. However, we found that upon ionomycin treatment clathrin was rapidly released from the plasma membrane in SK-MEL2 but not in HEK293 cells (ED 8C). Similarly, the plasma membrane bound pool of Dynamin-2-GFP was fully released 3 min after ionomycin addition. Indeed, late-stage CCPs disassembled with a ~4 times faster kinetic (rate constant $k_{(dynamin\ disassembly)} \pm$ CI95: 0.5262 $\pm$ 0.0691) compared to PS externalization ($k_{(PS\ assembly)} \pm$ CI95: 0.1278 $\pm$ 0.0564) (ED 8D). In line with this data, we found that ionomycin also fully blocks transferrin uptake in SK-MEL-2 cells (ED 8E). Thus, experimentally it was not possible to monitor lipid partitioning into CCPs after plasma membrane scrambling.

Together, our data indicates that the asymmetric lipid composition of the plasma membrane is a prerequisite for CME and that lipid asymmetry and CME are too closely linked to be functionally separated. This finding is in line with previous studies [6] and the reported role of lipids, such as PI(4,5,)P_2 and PS, to facilitate the binding of multiple proteins of the CME machinery [6,7].

With regard to the conclusion of this manuscript, we have adapted the discussion to account for this uncertainty. Nevertheless, we do think that this is a case were Ockham's razor applies. The respective sections now read as follows:

In addition, we found a strong correlation between melting temperature and CCP partitioning, with high melting temperature lipids partitioning more strongly into CCPs. This is in contrast to the expected depletion of high melting temperature lipids into curved membranes, based on previous reports [8–11] and the biophysical properties of a curved membrane [12,13]. Thus, it is likely that the observed lipid partitioning trends are not explained by classical L_o/L_d phase separation, which would result in the opposite partitioning behavior. In this context, it is an intriguing observation that melting temperature and lipid asymmetry are positively correlated, at least for the glycerophospholipids (ED 10). We have previously observed similar trends for the rates of non-vesicular lipid transport and metabolism for different PC species [3]. The initial step in all three processes is the mobilization/extraction of a lipid from the bulk bilayer by a protein (lipid translocases, lipid transfer proteins, lipid metabolizing enzymes). It is a compelling speculation that lipid mobilization, which should depend heavily on lipid biophysical properties, could be the rate limiting step in all cases. If this were the case, lipid biophysical properties would be a key determinant of trans-bilayer, intra- and inter-organelle lipid distribution.

• An important hypothesis that the authors discuss in the Discussion but do not test is that lipid-binding proteins may stabilize some lipid species at CME sites.

This is a good suggestion that provides a very distinct test for our model. Specifically, a differential amount receptor/ligand cargo could in principle influence the lipid composition within the CCP. We therefore measured the pit partitioning of 4 lipid species [PE(18:1/Y), PC(16:0/Y), PC(Y/20:4), SM(Y)] and 2 CCP localized transmembrane proteins: transferrin receptor (TfR) and LDL receptor (LDLR). We then analyzed whether a differential amount of TfR/ LDLR was correlated with a change in lipid composition within the pit. We found that the tested lipids largely show negligible to weak correlations with both receptors. Only the pit partitioning of SM(Y) correlates moderately with the pit partitioning of TfR, with a correlation coefficient of 0.343. These results indicate that at least constitutive protein cargo such as LDLR and TfR do not or only moderately affect lipid pit partitioning. It is possible that the positive correlation between SM(Y) and CCP proteins such as TfR increases the SM(Y) pit partitioning beyond what we would expect purely by the lipid asymmetry in the plasma membrane. Thus, these data are in line with our previous findings, further strengthening our argument that CME is mostly unselective with regard to lipid cargoes. One further important consideration in this context is stoichiometry. Lipids outnumber proteins ~ 50:1 in the plasma membrane. Thus, even if one considers the extreme case that every integral and peripheral membrane protein in a given CCP forms a high-affinity 1:1 lipid-protein complex, only 2% of the pit lipid content would be captured in these interactions. Thus, while the mechanism of pit enrichment by specific lipid-protein interactions would be a viable explanation for rarer individual lipid species, it is difficult to conceive how it could explain lipid sorting in bulk transport.

We have included our findings in the main text with a corresponding Extended Data figure. The respective sections now read as follows:
To understand which features could be responsible for the observed moderate yet significant differences in CCP partitioning, we first investigated whether the density of specific transmembrane CCP cargo (receptors) could affect lipid pit partitioning. Using 3-color STED imaging, we simultaneously measured the pit partitioning of a lipid (PC(16:0lY), PC(Yl 20:4), PE(18:1lY), or SM(Y)) and a trans-membrane receptor (Transferrin receptor (TfR) or LDL receptor

(LDLR)). Pit partitioning values of each lipid - receptor pair for late-stage CCPs were correlated using the Spearman's rank correlation (ED 7). Correlation analysis revealed negligible to moderate correlations for all pairs with correlation coefficients ranging from 0.041 to 0.343. These data show that moderate increases of lipid pit partitioning due to protein content can be expected for specific protein – lipid species pairs, as shown for SM(Y) and TfR with a moderate positive correlation (ρ: 0.343). However, overall, our findings indicate that the protein content of CCPs largely does not affect lipid partitioning for the receptors tested here, and does not explain the differences in lipid CCP partitioning.

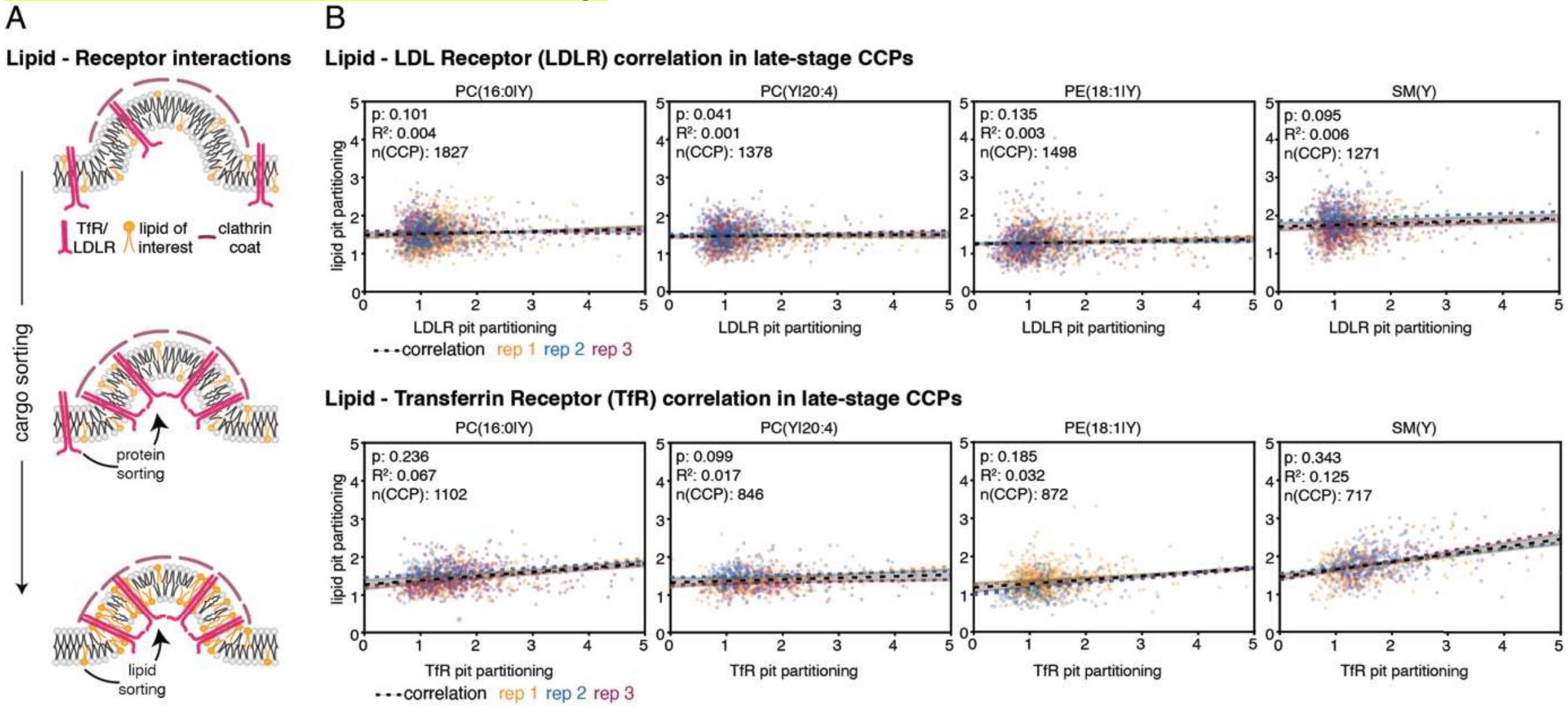

Extended Data 7: The receptor density in late-stage CCPs does not affect lipid pit partitioning. A: Cartoon of possible lipid sorting into CCPs by association with CCP-localized receptors. B: The pit partitioning of lipids (PC(16:0lY), PC(Yl 20:4), PE(18:1lY), or SM(Y)) and receptors (Transferrin receptor (TfR) or LDL receptor (LDLR)) were measured by 3-color STED imaging. Pit partitioning values for lipids and receptors are plotted per single late-stage CCP. Datapoints of each replicate are color-coded as indicated. The correlation was performed using the Spearman's rank correlation. The correlation coefficients (ρ), the coefficients of determination (R^2) and number of datapoints (n(CCP)) are indicated per lipid-receptor pair. The mean correlation (black dotted line) and standard deviation (shaded grey area) were determined from the correlations obtained per biological replicate. The experiment was performed in 3 biological replicates.

Minor comments:

• The Mund & Tchanz fixation protocol is cited by names, but does not match what is reported in the materials and methods.

Reviewer 3 is correct; there is a slight difference in the pH of the buffer (6.1 vs 7.2) and we used PFA rather than FA. However, the rest of the components are identical and as we so closely followed their protocol, we thought it appropriate to cite them. We have changed out formulation in the text to:

", and cells were fixed chemically using a protocol adapted from Mund and Tschanz et al. [24]"

Below the respective fixation conditions for reference:

Mund & Tchanz: “Cells were fixed as described previously (Li et al., 2018) using 3% (w/v) formaldehyde, 10 mM MES pH 6.1, 150 mM NaCl, 5 mM EGTA, 5 mM glucose, and 5 mM $MgCl_2$ for 20 min. “

Our fixation protocol: “Samples were fixed immediately after photoactivation for 20min at room temperature using a fixation buffer of 3% PFA, 10mM MES, 150mM NaCl, 5mM EGTA, 5mM d glucose, 5mM MgCl2, pH 7.2.”

• In general, there are several acronyms that are not defined (Ld, Lo) or are defined after use in the text (lipid names).

We now provide definitions at first use.

• Figure 1:

SK-MEL-2 are not typically round after being plated in the reported conditions. Cells shown in the Supplement exhibit the expected morphology. Choose a more representative image for this figure. Depicting how the exogenously added tagged lipids enter the inner leaflet in the cartoon would make the remainder of the paper easier to read.

We have included a different image in Figure 1 that better represents the typical morphology of SK-MEL-2 cells, and we have adapted the cartoon to include a lipid translocase.

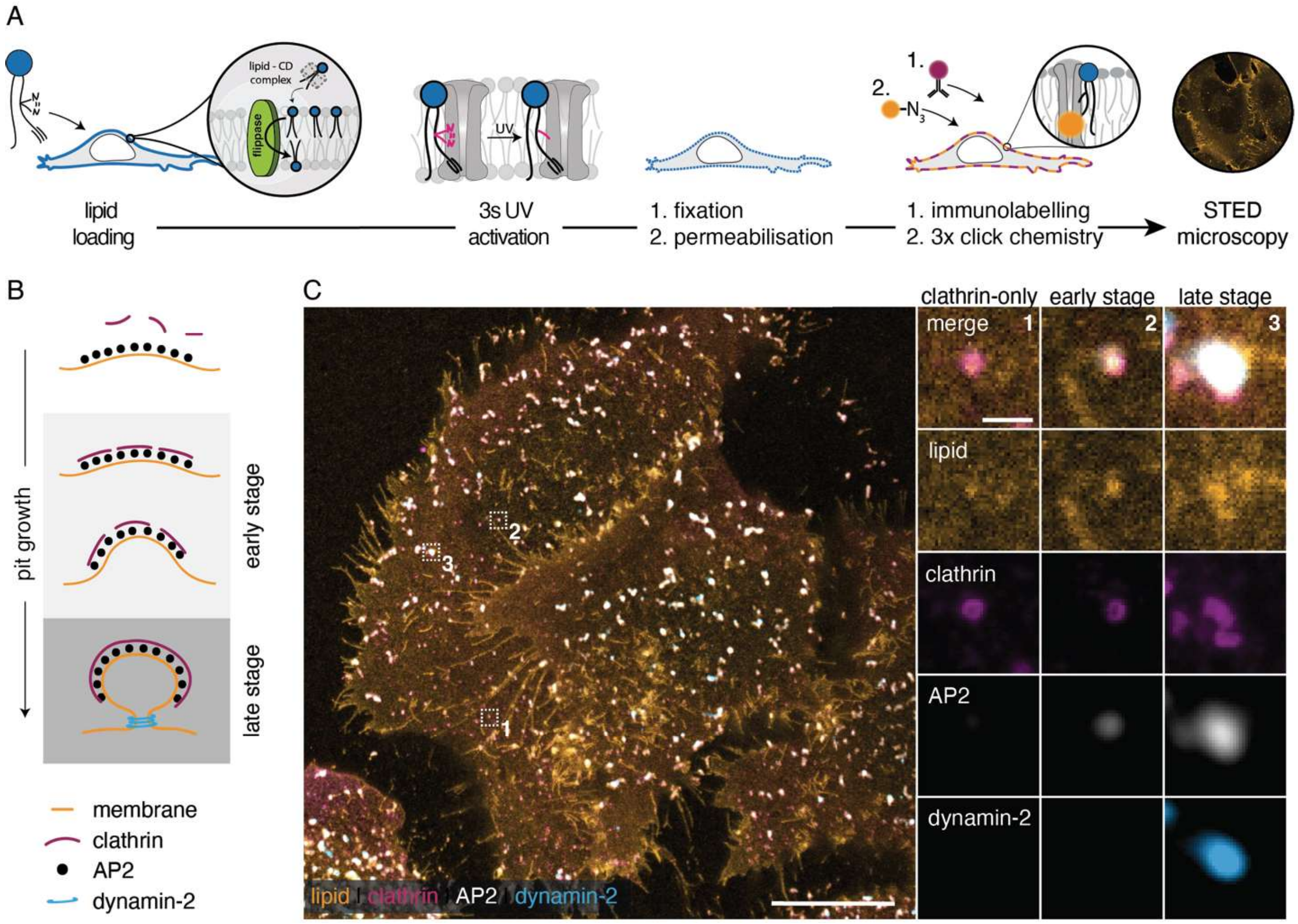

Figure 1: Super-resolution lipid imaging of lipid localization in clathrin-coated pits. A: Super-resolution lipid imaging workflow. Lipid probes were loaded into the plasma membrane of living cells using α-methylcyclodextrin. Photo-crosslinking to proteins with a 3s UV light pulse was followed immediately by chemical fixation. Samples were permeabilized, stained by immunolabelling and click chemistry, and imaged using 4-color confocal microscopy with the lipid and clathrin-channel super-resolved by STED. B: Stages of the growth of a CCP were classified using protein markers into clathrin-only structures, early-stage CCPs positive for AP2 and clathrin, and late-stage CCPs positive for AP2, clathrin, and dynamin-2. C: A representative STED image from 3 biological replicates of the basal membrane of a SK-MEL2 cell loaded with bifunctional PC(16:0lY) (orange) is shown, stably expressing dynamin-2-GFP (blue). Clathrin (purple) and AP2 (grey) were immunolabelled. Scale bars: 10μm (full image), 200nm (magnified areas).

Figure 1 B and Figure 1C subpanels mislead readers to expect time-resolved data.

The function of Figure 1B is to (1) provide an overview of the process of CME, which is necessary context for the manuscript and (2) to give the rationale for using the antibody markers that are used in the subsequent image analysis to classify pits. Figure 1C illustrates how pit structures were classified into the three categories “clathrin-only” “early-stage” and “late-stage”. This is exactly what we did in this manuscript and we need this figure panel as is to convey our pit classifications to the reader.

• Figure 2:
• Remove modeling of expected intensities for early-stage CME sites.

We chose to keep this in the manuscript, as it provides necessary context, as also discussed above in our answer to the “temporal resolution” point

• Fig2A: The outline of the CME site in the last panel changes color, and is confusing to the reader.

We have adapted the figure accordingly.

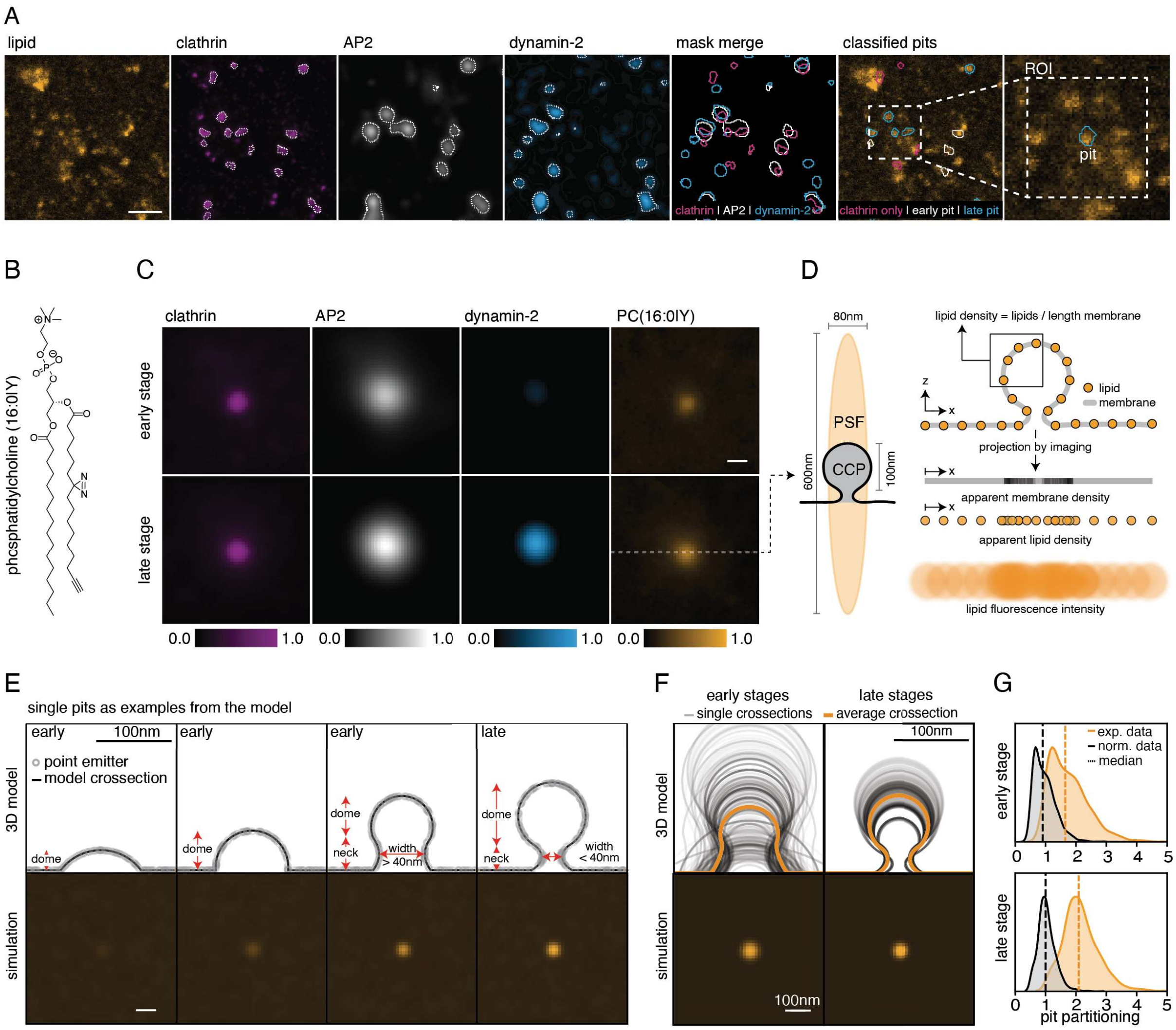

Figure 2: Quantification of lipid partitioning in clathrin-coated pits. A: Image analysis pipeline. The super-resolved clathrin signal (purple) was used to generate a segmentation mask (white dotted overlay). Pits were classified as clathrin only (purple), early-stage (white), or late-stage pits (blue), depending on their overlap with the AP2 and dynamin-2 masks generated from the respective channels. Apparent lipid partitioning was calculated by measuring the mean lipid intensities of individual pits and division by the mean lipid intensity of the surrounding plasma membrane (ROI). Scale bar: 1μm. B: Chemical structure of PC(16:0/Y). C: Average 4-color fluorescence images for early- and late-stage pits. Fluorescent images of CCPs were centered on the centroid of the clathrin mask before averaging. Scale bar: 100 nm. Averages were acquired from 3 biological replicates. N(early CCPs): 3990, n(late CCPs): 2801. D: A scheme is shown on the left for the dimensions of the experimentally determined point spread function of the microscope in relation to a late-stage CCP. On the right, the process of convolution during imaging is depicted. A pit cross-section with even point emitter distribution is projected into one dimension, resulting in higher apparent membrane densities and lipid densities at the region of the pit invagination. After blurring by the PSF of the microscope, the pit appears brighter compared to the surrounding flat membrane. Lipid density as a readout for lipid partitioning must be determined as the ratio of lipids over surface area. E: Top panels: Cross-sections of modeled CCPs. Bottom panels: Simulated STED for the displayed pit geometries. Scale bars: 100 nm. Representative simulations are shown

from a pool of 2000 simulations. F: Average cross-sections (black) of all simulated pits (grey) and averaged simulated images are shown. Scale bar 100 nm. Averages contain 1000 cross-sections for early and late stages each. G: The pit partitioning distributions of early and late-stage pits are shown for PC(16:0lY) before (orange, exp. data) and after (grey, norm. data), normalizing to the median of the model. The respective median of the data is shown as a dotted line.

- Figure 3: Consider moving Fig 3A to Fig1, so that the readers (1) understand the lipid naming scheme used in the paper text and (2) establishes the lipids used in the studies to establish expectations for the readers.

We would like to keep the current figure composition. The logic of the manuscript is to establish the methodology (Figure 1) and data analysis strategy first, then proceed to data analysis and modelling concepts (Figure 2) and after establishing the overall methodology to proceed with comparative datasets and interpretation in subsequent figures. While this may be a bit redundant for experts in membrane trafficking / lipid biology we do think that this structure is better suited for the broader readership of NCB.

References (Answer to the reviewers)

1. Yu, K. *et al.* Identification of a lipid scrambling domain in ANO6/TMEM16F. *eLife* 4, e06901 (2015).
2. Boccaccio, A. HEK293 Cell–Based Assay to Measure the Lipid Scrambling Activity of TMEM16 Family Members. in *Intracellular Lipid Transport: Methods and Protocols* 119–132 (Springer US, New York, NY, 2025).
3. Iglesias-Artola, J. M. *et al.* Quantitative imaging of lipid transport in mammalian cells. *Nature* 646, 474–482 (2025).
4. Barahtjan, P. *et al.* Disruption of Plasma Membrane Lipid Asymmetry Alters Cellular Energetics. *bioRxiv* 2025.04.27.650841 (2025) doi:10.1101/2025.04.27.650841.
5. Gurdap, C. O. *et al.* Cell surface remodeling caused by the loss of TMEM30A in immune cells. *bioRxiv* 2026.02.21.707187 (2026) doi:10.64898/2026.02.21.707187.
6. Zoncu, R. *et al.* Loss of endocytic clathrin-coated pits upon acute depletion of phosphatidylinositol 4,5-bisphosphate. *Proc. Natl. Acad. Sci.* 104, 3793–3798 (2007).
7. Godlee, C. & Kaksonen, M. From uncertain beginnings: Initiation mechanisms of clathrin-mediated endocytosis. *J. Cell Biol.* 203, 717–725 (2013).
8. Lamaze, C. *et al.* Interleukin 2 receptors and detergent-resistant membrane domains define a clathrin-independent endocytic pathway. *Mol. Cell* 7, 661–671 (2001).
9. Nichols, B. J. GM1-Containing Lipid Rafts Are Depleted within Clathrin-Coated Pits. *Curr. Biol.* 13, 686–690 (2003).
10. Sabharanjak, S., Sharma, P., Parton, R. G. & Mayor, S. GPI-Anchored Proteins Are Delivered to Recycling Endosomes via a Distinct cdc42-Regulated, Clathrin-Independent Pinocytic Pathway. *Dev. Cell* 2, 411–423 (2002).
11. Lorent, J. H. *et al.* Structural determinants and functional consequences of protein affinity for membrane rafts. *Nat. Commun.* 8, 1219 (2017).
12. Callan-Jones, A., Sorre, B. & Bassereau, P. Curvature-Driven Lipid Sorting in Biomembranes. *Cold Spring Harb. Perspect. Biol.* 3, a004648 (2011).
13. Sorre, B. *et al.* Curvature-driven lipid sorting needs proximity to a demixing point and is aided by proteins. *Proc. Natl. Acad. Sci.* 106, 5622–5626 (2009).
14. Koukalová, A. *et al.* Lipid Driven Nanodomains in Giant Lipid Vesicles are Fluid and Disordered. *Sci. Rep.* 7, 5460 (2017).
15. Davidović, D. *et al.* Which Moiety Drives Gangliosides to Form Nanodomains? *J. Phys. Chem. Lett.* 14, 5791–5797 (2023).
16. Iglesias-Artola, J. M. *et al.* Quantitative imaging of species-specific lipid transport in mammalian cells. *bioRxiv* 2024.05.14.594078 (2024) doi:10.1101/2024.05.14.594078.
17. Lennartz, H. M. *et al.* Visualizing suborganellar lipid distribution using correlative light and electron microscopy. *Nat. Cell Biol.* 28, 828–837 (2026).
18. Kirschbaum, C. *et al.* Bifunctional Lipid–Protein Cross-linking Efficiency and Reaction Products. *J. Am. Chem. Soc.* 148, 15349–15353 (2026).
19. Taylor, M. J., Perrais, D. & Merrifield, C. J. A High Precision Survey of the Molecular Dynamics of Mammalian Clathrin-Mediated Endocytosis. *PLOS Biol.* 9, e1000604 (2011).
20. Roux, A. *et al.* Membrane curvature controls dynamin polymerization. *Proc. Natl. Acad. Sci.* 107, 4141–4146 (2010).
21. D'Angelo, G. & La Manno, G. The lipotype hypothesis. *Nat. Rev. Mol. Cell Biol.* 24, 1–2 (2023).
22. Capolupo, L. *et al.* Sphingolipids control dermal fibroblast heterogeneity. *Science* 376, eabh1623.
23. Parton, R. G., Kozlov, M. M. & Lundmark, R. A lipid-centric view of endocytosis by caveolae. *Nat. Cell Biol.* (2026).

24. Mund, M. *et al.* Clathrin coats partially preassemble and subsequently bend during endocytosis. *J. Cell Biol.* 222, e202206038 (2023).

Quantification of lipid sorting during clathrin-mediated endocytosis

Corresponding Authors: Prof. Dr. Alf Honigmann, Dr. André Nadler

General remarks:
We thank all reviewers for their comments, which we address in full in our manuscript.
Below are the full reviewer comments and our answers in light blue.

Reviewer #1 (Remarks to the Author):

I commend the authors on their (typically) rigorous and thorough revision. The new experiments add quite a lot to the manuscript, addressing the main caveat (probe behavior) directly and quantitatively. I was supportive of this story originally and now am convinced that it should be published without delay. Congrats to the authors on important and rigorous work!

We thank reviewer #1 for the support and the constructive feedback during the first revision that made our manuscript much stronger.

Reviewer #2 (Remarks to the Author):

I appreciate the revision efforts, which clearly addressed my previous comments.

We thank reviewer #2 for the support and the constructive feedback during the first revision that made our manuscript much stronger.

Reviewer #3 (Remarks to the Author):

We thank the authors for their thorough response and for dedicating significant effort to addressing our previous comments. The additional experimental data and clarifications have markedly strengthened the manuscript. However, there remain a few final points that we believe require minor text adjustments and clarification prior to publication.

We thank reviewer (s?) #3 for their thorough during the first revision round and their support. We address the remaining minor points by textual changes

1. PIP2 and PS trafficking point: Because lipids like PIP2 and PS template the initiation of CME sites, it remains highly possible that CME actively regulates CME site plasma membrane distribution. These specific species were not explicitly tested in this study. However, the manuscript text frequently refers to "lipids" in a general sense. To avoid confusing the reader, it is crucial to explicitly state the caveat that the specific probes evaluated here may not reflect the trafficking behavior of these key regulatory lipids.

The authors modified the text to read: "We note that our findings here do not preclude sorting of individual lipids during clathrin-independent endocytic processes, with caveolae-mediated endocytosis proposed as one possible lipid sorting mechanism 23, but these mechanisms are likely minor components in the total transport volume of lipids between the plasma membrane and other organelle membranes."

We feel a more precise and appropriate clarification for the reader would be: "While our results indicate that clathrin-mediated endocytosis exhibits only limited selectivity for cytoplasmic leaflet lipids, our analysis was not intended to comprehensively assess all lipid species. In particular, we excluded PIP2 and PS because they are well-established regulators of CME, and their inclusion would confound our ability to test whether the CME machinery itself drives the sorting of PIP2 and PS. Consequently, we cannot exclude the possibility that CME selectively regulates the distribution of these specific lipid species."

We include the suggested text by reviewer #3 in the text in a slightly reworded fashion to account for better flow of the text. We note that we keep the original statement with regard to different endocytic routes as this provides necessary context for the reader. The entire section now reads: We note that our findings here do not preclude sorting of individual lipids during clathrin-independent endocytic processes, with caveolae-mediated endocytosis proposed as one possible lipid sorting mechanism [55], but these mechanisms are likely minor components in the total transport volume of lipids between the plasma membrane and other organelle membranes. We furthermore note that our analysis covered a subset of all lipid species present in the plasma

membrane. In particular, we excluded PIP2 and PS because they are well-established regulators of CME, and their inclusion would confound our ability to test whether the CME machinery itself drives the sorting of PIP2 and PS. Consequently, we cannot exclude the possibility that CME selectively regulates the distribution of these specific lipid classes.

2. Geometric modeling point: We were expecting estimates of the expected differences in maximal lipid numbers observed in the inner vs outer leaflets for a select number of relevant lipids, and apologize for the lack of specificity in the request. While it would have been nice to see, we are content with this topic being omitted from the manuscript, given the strength of the existing modeling.

We apologize for the misunderstanding.

3. Ionomycin biological perturbation: We thank the authors for their diligent attempt to address our hypothesis using acute membrane scrambling. Although the rapid disassembly of clathrin-coated pits (CCPs) prevented a direct test of the original partitioning hypothesis, documenting this structural dependence provides a valuable and interesting record for the endocytosis literature.

We agree with the assessment of reviewer #3.

4. Receptor density experiment: We appreciate the rigorous effort that went into executing this new cargo density correlation analysis. To better match the definitive nature of the data, we recommend adjusting the title of Extended Data 7 to read: "Extended Data 7: The receptor density in late-stage CCPs does not predict lipid pit partitioning. "

Agreed. We have changed the title of the ED figure as requested.

5. Finally, there are a few typographical errors in the text that must be corrected during final production: For example: "Dymanin2" should be corrected to "Dynamin2" in the Acknowledgments; "Life cell imaging" should be "live cell imaging" in Extended Data 8; and "diplamitoylphosphatidylcholine" should be "dipalmitoylphosphatidylcholine" in Reference 38.

The errors were corrected.
